# The genera *Chrysilla*and *Phintelloides*revisited with the description of a new species (Araneae, Salticidae) using digital specimen DOIs and nanopublications

**DOI:** 10.3897/BDJ.12.e129438

**Published:** 2024-09-03

**Authors:** Christa L. Deeleman-Reinhold, Wouter Addink, Jeremy A. Miller

**Affiliations:** 1 Naturalis Biodiversity Center, Leiden, Netherlands Naturalis Biodiversity Center Leiden Netherlands; 2 Sparrenlaan 8, 4641 GA, Ossendrecht, Netherlands Sparrenlaan 8, 4641 GA Ossendrecht Netherlands; 3 Distributed System of Scientific Collections - DiSSCo, Leiden, Netherlands Distributed System of Scientific Collections - DiSSCo Leiden Netherlands; 4 Plazi, Bern, Switzerland Plazi Bern Switzerland

**Keywords:** Biodiversity informatics, canopy fogging, copulatory mechanics, discoloration, specimen preservation, tropical Asian jumping spiders

## Abstract

**Background:**

Two Southeast Asian spider collections: that of Frances and John Murphy, now in the Manchester University Museum and the Deeleman collection, now at the Naturalis Biodiversity Center in Leiden constituted the basis of this analysis of *Chrysilla* Thorell, 1887 and related genera. The latter collection also includes many thousands of spiders obtained by canopy fogging for an ecological project in Borneo by A. Floren.

**New information:**

Some incongruences within the genera of the tribe Chrysillini are disentangled. The transfer of *C.jesudasi* Caleb & Mathai, 2014 from *Chrysilla* as type species of *Phintelloides* Kanesharatnam & Benjamin, 2019, based on analysis of molecular data is validated by morphology. An interesting new species known only from the forest canopy in Borneo, *Phintelloidesscandens* sp. nov, is described based on both male and female specimens. Distinguishing chrysilline genera is mostly based on traditional somatic characters, e.g., habitus, carapace and abdomen patterns, mouthparts, and genital organs. The utility of two character systems for distinguishing chrysilline genera is highlighted: 1) the presence of a flexible, articulating embolic tegular branch (etb) in combination with the conformation of the characteristic construction of the epigyne in *Chrysilla* and *Phintelloides*; 2) presence of red colour on carapace and abdomen of live males and females, in combination with abundant blue/violet/white iridescent scales such as in *Chrysilla* and *Siler*. The red colour usually gets lost in alcohol, hampering species identification of alcohol material. The genera *Chrysilla* and *Phintelloides* are redefined. Specimens of the heretofore unknown female of *Chrysilla* *deelemani* Prószyński & Deeleman-Reinhold, 2010 are described. The male and female of *Chrysillalauta* and male of *C.volupe* are redescribed. The genus *Chrysilla* is diagnosed and discriminated from *Phintella*Bösenberg & Strand, 1906, *Siler*Simon, 1889*,* *Phintelloides* Kanesharatnam & Benjamin, 2019 and *Proszynskia* Kanesharatnam & Benjamin, 2019*.* The structure of the female genital organ of *Phintelloidesflavumi* Kanesharatnam & Benjamin, 2019 is scrutinized and the generic placement of *Phintelloides* is discussed. Males and females of one of the most variable species*, Phintelloidesversicolor* (C. L. Koch, 1846) are redescribed. *Phintelloidesmunita* (Bösenberg & Strand, 1906) is removed from synonymy with *P.versicolor. Phintellaleucaspis* Simon 1903 (male, Sumatra) is synonymized with *P.versicolor*.

Biodiversity data are increasingly reliant on digital infrastructure. By linking physical specimens to digital representations of their associated data, we can lower barriers to information flow. Here we demonstrate a workflow whereby persistent identifiers (PIDs) in the form of DOIs issued by DataCite are assigned to specimens. Recognized taxa are identified by their catalog of life identifier, or by registration in ZooBank where no catalog of life identifier is available. We demonstrate the use of nanopublications, creating a series of machine readable, scientifically meaningful assertions regarding the provenance and identification of cited specimens. All human agents associated with the specimen data are linked to a persistent identifier issued by either ORCiD or Wikidata.

## Introduction

Jumping spiders (family Salticidae) attract attention as a highly diverse taxon (>6600 species described across >680 genera, World Spider Catalog 2024) featuring colorful, day active, visually oriented species; this is especially true of the tropics. In some, such as the members of the tribe Chrysillini (Maddison 2015: 247), live specimens attract attention with sparkling colours: silvery white, violet, blue, green and red, often borne on iridescent scales and appressed setae on the integument of the carapace, abdomen and male palps. Unfortunately, colours may change or disappear altogether in alcohol preserved specimens. This can make preserved specimens and live animals of the same species appear so different that they are challenging to recognize as conspecifics. Compounding this taxonomic challenge, males and females are often dissimilar in habitus making it difficult to match sexes; a relatively low proportion of species are known from both sexes. It is not unusual to find species of Chrysillini that have had males and females described as separate species.

It is well known that in the tropics, fauna and flora are generally more diverse than in colder climates. The tropical rainforest, the most species-rich terrestrial habitat in the world, hosts the highest number of unknown, undescribed arthropod species. The forests of tropical Asia are among the world’s tallest, characterized by emergent trees such as *Dipterocarpus*. Such trees may grow up to a height of 40-80 meters. Perhaps 99% of the species known from tropical forests have been collected from the lower 2 meters. Although we lack a rigorous estimate of the degree to which the canopy fauna is distinct from that of the lowest stratum, collections from forest canopy are a rich source for novel discovery. This publication is the latest in a series based on the unique and remarkable collection of more than 10,000 spiders collected by A. Floren during a long-term ecological project on Borneo (van Dorp 2020). This collection includes an unknown number of remarkable species that are quite unlike relatives from the understorey (Floren and Deeleman-Reinhold 2005), or are geographically distant from their closest known relatives (Deeleman-Reinhold et al. 2016). It is clear from the many new discoveries derived from the modest samples available that these forests harbour a profusion of undiscovered species. In the face of intensifying anthropogenic environmental change, we hope that fundamental research on the biodiversity of this critical region can be supported. Contemporary practices in international taxonomic research emphasize data mobilization as a mechanism for maximizing value of biodiversity data. This means lowering technological and social barriers to sharing, aggregating, and applying data flexibly to serve a broad spectrum of stakeholders, and providing data resources and inspiration to future generations of scientists.

The genera of Chrysillini that have been selected for this study belong to the core group of genera, characterized by the presence of a tegular bump on the male pedipalp (Fig. 1a, Maddison 2015: 247). A phylogenetic study of the Chrysillini explored in part the evolution of their conspicuous coloration (Kanesharatnam and Benjamin 2019). The most eye-catching genera in the group, such as *Chrysilla*, *Siler, Cosmophasis,* and *Orsima*, exhibit iridescent colours (such as red, green, blue and violet) on setae and scales on the cephalothorax, abdomen and male palps. Others, such as *Phintella, Phintelloides,* and *Proszynskia*, express more modest colours (such as black, white, brown and yellow). Photos of live spiders (Fig. 2) can be found in the field guides of Borneo and Singapore (Koh and Bay 2019, Koh et al. 2022, Koh and Ming 2013), taxonomic publications (e.g., Caleb et al. 2018, Yamasaki et al. 2018), and online databases (Metzner 1996-2020, Prószyński 2016). It is clear that some genera in this group have been ill defined in the past as witnessed by the frequent transfer of species between genera. In particular, the genus *Chrysilla* has often been misinterpreted. For much of the history of *Chrysilla*, no species were known from both sexes. Female *Chrysilla* species have an epigyne with a characteristic structure. *Chrysilla* are usually sexually dimorphic; *C.acerosa* Wang & Zhang, 2012 is an exception. Kanesharatnam and Benjamin (2019) recently established the genus *Phintelloides* for a set of new species from Sri Lanka, revealing a remarkable radiation. *Phintelloides* species are united by putative synapomorphies, such as specific black and white patterns on the carapace, the shape of the tegulum in the male palp, and the path of the copulatory ducts of the epigyne. 

Colour pattern can be useful for species identification in such flamboyant spiders. Unfortunately, there is an inconvenient discrepancy between colours in live or freshly preserved specimens, and specimens that have been preserved in alcohol for some time. In most cases, we found that long preserved specimens (for example, >20 years) had lost nearly all colour. Red is among the colours most prone to disappearing in alcohol. Label data sometimes record color notes. Red areas visible on photographs of live animals may appear in preserved specimens as bare, pale brown, usually without any setae, hairs or scales at all.

Pigment colouration can be supplemented by regions with tiny iridescent multicolored scales and flattened setae. Several of the alcohol preserved specimens we examined were swimming in clouds of floating colourless scales of various size. Some iridescent scales bearing blue, violet, or green can be retraced on the teguments of carapace and abdomen but the colours were not always the same as that on photos*;* in both live and alcohol specimens, colours may change when shifting direction of viewing or change of position of light source. Iridescent blue and violet usually are associated with round scales, the size of these scales may differ on different parts of the body.

### Copulatory mechanics in *Chrysilla*and *Phintelloides*

The male pedipalp of *Chrysilla*and *Phintelloides*features an atypical configuration. The tegulum is cleft into pro- and retrolateral parts: the prolateral branch we call embolar tegular branch (etb), with the embolus proper (ep) sitting on top; the base of the etb is attached dorsally, hidden by the larger retrolateral part (rt) containing the U-shaped spermduct-loop (sdl). The etb is long and flexible, freely movable, articulated at the base with the proximal tegular lobe (pt). This can be ascertained by manual examination with fine forceps and needle. The structure of the epigyne is likewise unusual, having copulatory ducts distally diverging as a bird-neck-shaped curve (bnc) with often inflated walls (possibly glands?) directed outwards, ending as an open bird’s beak (here called atrium, a); it lacks a distinct copulatory opening. Also, the pockets in the posterior ridge of the epigyne (pp) are rigid and probably play a role in anchoring the proximal part of the palp. 

**Figure 5.**
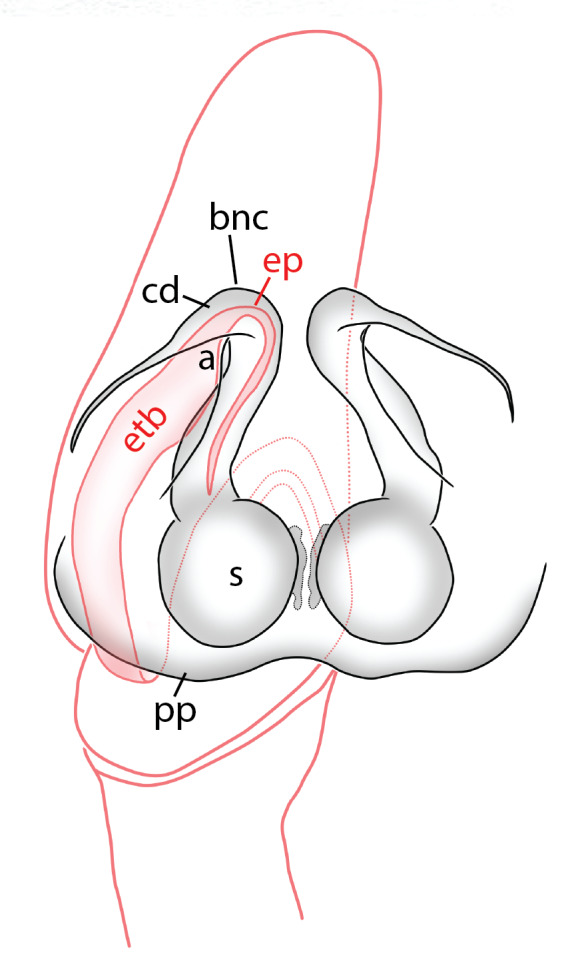
*Chrysillalauta* Thorell, 1887, schematic illustrations showing hypothetical interaction between male and female genitalia **a** atrium **bnc** bird’s neck curve **cd** copulatory duct **ep** embolus proper **etb** embolar tegular branch **pp** posterior pockets **s** spermatheca

In search for the copulatory opening, CLD-R separated a palp and a cleared epigyne from specimens of *Phintelloidesscandens* sp. nov. and of *Chrysillalauta* (Figs 5, 16) and an epigyne of *Phintelloidesflavumi* (Fig. 11) and manipulated them, measuring the lengths and widths testing if these would allow a transfer of sperm into the spermatheca by introducing the tegulum and embolus inside the vulva (Figs 5, 16; tegulum in yellow, etb in red). In each species, the length of the embolus proper proved to match the length of the copulatory duct. 

In females of *P.scandens* viewed from the ventral aspect, two round adjacent excavations are seen on in the left and the right part of the epigyne (Figs 11c, d, 16). The outer one, the atrium (Fig. 16: a), confined between the outer (ora) and the inner (mra) atrium rim, encompasses the copulatory opening (co) and during copulation hosts the etb. The inner excavation is separated from the atrium by the median rim (mra) and is confined by the proximal wall of the bird-neck (bnc). It is blind and matches the shape and size of the retrolateral part of the tegulum (rt). The total length of the tegulum just about equals the distance between the pockets in the posterior ridge in the epigyne (pp) and the inner excavation. The shape of the distal part of the etb matches the shape of the atrium and its length equals the length of the ora and mra so that the embolus proper (ep) can be pushed into the copulatory opening. This supports the idea that during copulation the tegulum is lengthwise pinched between the posterior pocket and the inner excavation and provides a grip for the stability of the male palp while pushing the etb into the atrium and the embolus into the copulatory ducts.

In *P.flavumi*, as can be seen in the vulva oriented in oblique ventro-lateral view (Fig. 11c, d), the atrium is clearly seen in the shape of a funnel which would be suitable to lodge the etb, so that the embolus can be pushed through the copulatory opening (co). Behind it the inner (lower) bend of the bird-neck-curve can be seen, providing a rounded excavation, suitable to anchor the tegulum tip.

In *C.lauta* the hypothetical functioning during copulation is also visualized in ventral view (Fig. 5). The inner excavation is obscured by the bird neck curve which is inclined ventrally. A large atrium is seen between the outer and the inner atrium rim leading to the copulatory opening. By contrast with *P.scandens*, in *C.lauta* the total length of the tegulum exceeds the distance between the posterior pocket and the atrial cavity. However, the proximal part of the tegulum in *C.lauta* is considerably swollen ventrally (Fig. 1b, d); an attempt to visualize the possible mating positions is made in Fig. 5, where the tegular bulge (tbu) is anchored against the posterior ridge of the epigyneal pocket (pp); the etb (red) is inserted into the atrium, the embolus (ep) inside the copulatory duct, the retrolateral part of the tegulum (yellow) distally resting on the ventral surface of the epigyne; the contours of the palp positioned on the ventral surface of the epigyne is presented as red dotted line. A similar construction in male and female genital organs is found in the genus *Bristowia*Reimoser, 1934 (Szüts 2004).

## Materials and methods

A stereomicroscope Zeiss Stemi SV11, ocular 10 x objective zoom 0.8 - 6.6 x, with Muiji halogen microscope lamp and Schott glass fiber optic lighting was used for examination and photography. Drawings were made with Zeiss drawing tube with drawing pens MICRON 1,0, 3,0 5,0 and 7,0 mm, lead pencils H, B and 2B and H, 3B and 8B cretacolor on special drawing paper. Photographs were made with a DS-R:1 digital camera driven by NIS Elements software with composite extended focus images generated using Helicon Focus 7 (Kozub et al. 2000). Additional photographs were made using a Nikon J5 digital camera. Measurements were made by using an ocular micrometer and reported in millimeters. Body lengths exclude protruding eye lenses and spinnerets; leg length measured on the dorsal side. Epigynes were detached for examination and temporarily immerged in clove oil for clearing.

The following abbreviations are used in the text and figures:

- a - atrium
- AME - anterior median eyes
- beb – base of embolar tegular branch
- bnc – bird’s neck curve
- cc - cymbium cap, distance between distal edge of alveolus and top of cymbium
- cd - copulatory duct or insemination duct
- CM – Collection Murphy, in MMUE
- co - copulatory opening
- ep – embolus proper
- etb - prolateral embolar tegular branch
- fd - fertilisation duct
- ibc - inner bend of bird's-neck shaped curve
- MMUE – Manchester Museum, University of Manchester, U.K. (Arzuza Buelvas 2018)
- mra - median rim of atrium
- ora - outer rim of atrium
- p – projection of prolateral embolar tegular branch (etb) beyond retrolateral lobe of tegulum (rt), exluding embolus proper (ep)
- pt – proximal tegular lobe
- pp - posterior pockets
- RMNH – Naturalis Biodiversity Center, Leiden, NL, formerly Rijksmuseum van Natuurlijke Historie
- rt – retrolateral part of tegulum
- rta - retrolateral tibial apophysis
- s - spermatheca
- sdl - sperm duct loop
- tbu - tegular bump

Total length is measured exclusive of AME lenses and spinnerets. Leg measurements are presented thus: total (femur – patella – tibia – metatarsus – tarsus). All measurements in milimetres.

Hairs are thin filiform, erect or appressed; setae are elongate acuminate, flattened, sometimes appressed and iridescent; scales are round, thin and iridescent.

### Use of identifiers

Digital Object Identifiers (DOIs) are globally unique, resolvable and persistent unique identifiers that are in widespread use for citing publications. In the realm of biodiversity informatics, they have been adopted to identify and electronically link multiple classes of data objects within taxonomic publications. Journal publishers like Pensoft as well as the biodiversity data group Plazi have developed workflows for biodiversity publications which issue DOIs for elements within the publication, such as figures, taxonomic treatments, and supplementary data (Penev et al. 2009, Fawcett et al. 2022, Miller et al. 2015). Taxonomic treatments are the content within a taxonomic publication concerned with one particular taxon, such as a species description including its diagnosis, figures, specimens, and other data (Agosti and Egloff 2009, Miller et al. 2015). The paper you are reading now contains 10 treatments, two generic treatments and 8 species treatments. The EU research project BiCIKL is an European Union Horizon 2020 biodiversity informatics infrastructure project that is innovating in the area of data mobilisation by means of persistent unique identifiers (Penev et al. 2022).

In this contribution to spider taxonomy, we demonstrate an innovation in the mobilisation of biodiversity data. All specimen records cited herein have been assigned a digital object identifier (DOI; Table 1) that points to a digital representation of the specimen, a digital specimen, which can become a digital extended specimen by digitally linking it to relevant ecological, environmental, and related data from numerous domains (Hardisty et al. 2022). This contribution from the BiCIKL project proposes an electronic extension of a physical object archived in a natural history collection and digitized through its institutional collections database (Addink et al. 2023).

The digital specimen is a mutable and versioned FAIR Digital Object (FDO) that is machine actionable through inclusion of a data type definition, a machine readable description of its data structure and allowed operations which is included in the metadata of the DOI record. Machine actionablilty allows systems to act upon the data by for example adding annotations with new information. Digital Specimen DOIs include, in contrast with most other DOIs, more metadata in the DOI record than only the URL to which it should redirect. This can be seen by specifying the noredirect parameter with the DOI, a feature of the Handle system on which DOIs are build, for example: https://doi.org/10.3535/1CE-SXA-2BC?noredirect. This allows the retrieval of some metadata describing the object without having to retrieve the full data object. It allows machines to quickly navigate billions of objects but can also be used in applications to provide a user with extra information about the object referenced by a DOI before going to its HTML landingpage, like the type of specimen, its name or its catalog number. Also, these DOIs implement multiple redirects (another feature of the Handle system): one for machines pointing to a JSON version of the data and one for humans pointing to a HTML landingpage. This can be further extended with for example a redirect directly to the bit-sequence of the object, which is useful for specimen images. In other words: a machine can decide to either retrieve the full digital media object including metadata and annotations, or directly retrieve the binary image file. The first digital specimen DOIs in existence have been created for this publication through DataCite by making use of FDO infrastructure developed by DiSSCo.

Another innovation we demonstrate in this publication is the use of nanopublications, which are supported by the Pensoft Arpha journal system. A nanopublication is a scientifically meaningful assertion about something, that can be uniquely identified and attributed to its author, its original source (provenance) and citation record (publication info). A nanopublication can directly link a scientific paper to the specimens it discusses together with provenance information, like: "This digital specimen DOI is discussed in this article DOI, published on [date] by [author]". This makes it very easy to digitally track which specimens are cited in which publication and by whom. 

In addition, all human agents associated with these specimens (for example, collectors) have been linked through a machine readable persistent unique identifier (Table 2). Where possible, we found existing identifiers such as ORCiD or Wikidata. Where no such pre-existing identifier could be found, we created records in Wikidata. 

Taken together, the use of machine readable persistent unique identifiers, constructed according to FAIR principles to facilitate the widest possible exchange of data, facilitate the linking of biodiversity data elements that are both logical and flexible. Biodiversity data are a challenge for several reasons, but they include both magnitude (occurrence records for all species across space and time), and the multiple forms of physical objects and electronic data that contribute to this sphere of knowledge. In an era of biodiversity crisis, the importance of an effective infrastructure to facilitate the storage and recall of these data and linked objects is coming into clear focus.

## Taxon treatments

### Chrysilla

Thorell, 1887

0D9462C8-DAB5-5752-BA64-36EA50D1D855

https://www.checklistbank.org/dataset/288943/taxon/62LN8

World Spider Catalog: urn:lsid:nmbe.ch:spidergen:02890
Chrysilla Thorell, 1887 - Thorell 1887
Chrysilla lauta Thorell, 1887Thorell 1887: 378.

#### Description

Middle-sized (body length 3.2–7.2 mm) unidentate, sexually dimorphic spiders. Carapace profile sloping down directly behind the eyes in a straight line. Chelicerae in males simple, elongated and sometimes divergent, in females parallel. In males, leg I dark and longer than the others, other legs pale, in females all legs pale and leg I proportional; both sexes with some black rings on leg IV. Spination of legs: femur I-IV with 1-1-1d, tibia I and II with 2-2-2 v or 2–2-2-1 v, metatarsus I and II with 2-2 v in both sexes; in *C.lauta* ventral spines on tibia I and II very strong, in other *Chrysilla species* front legs usually not so strongly armed. Abdomen in males about 1½ – 2 times longer than carapace, in female shorter and more rounded. The dorsal pattern is variable between species.

#### Diagnosis

*Chrysilla* can be distinguished from other chrysillines by the following set of characters: **1)** – body colour: live specimens are conspicuously coloured in patterns of white, black, red, iridescent blue or green; in specimens kept in alcohol, the red colour rapidly disappears. Similar colours are also found in *Siler*Simon, 1889; **2)** – clypeus: *Chrysilla* *lauta* Thorell, 1887, *C.volupe* (Karsch, 1879), *C.deelemani* Prószyński & Deeleman-Reinhold, 2010, and *Proszynskia* Kanesharatnam & Benjamin, 2019 lack a bunch of long white setae and have only dark metallic scales on the clypeus (in life), whereas a white bunch on the clypeus is characteristic for *Phintelloides.* However, the description of *Chrysillaacerosa* Wang & Zhang, 2012 is provided with numerous colour photos, one of which clearly shows bundles of white flattened setae in front of the AME; **3) **– thorax margins: in* Chrysill*a and *Siler* *semiglaucus,* both sexes have the thorax sides lined by a narrow strip of iridescent scales (Kanesharatnam and Benjamin 2019, fig. 21A; Yamasaki et al. 2018, fig. 11), whereas *Phintelloides* and *Phintella* have a wide band of white flattened setae along the margin of the thorax in both sexes; **4)** – abdomen: in all known *Chrysilla* species, males have a long, cylindrical abdomen, about three times longer than wide and clearly narrower than the carapace, sometimes with a dorsal scutum covered with colourful iridescent scales. In the field, the species can be recognized by their colour pattern. In females, the abdomen is notably shorter. By contrast, the male abdomen in *Phintella* and *Phintelloides* is shorter and more rounded, occasionally with something like a scutum; **5) **– cymbium length: in *Chrysilla*and *Phintelloides*the slender palp has a long cymbium cap (cc) with parallel sides, measuring more than half the bulbus length. This contrasts with *Phintella*, which has a cc of less than half the bulbus length, and *Siler*, which has a short cc that protrudes only barely beyond the tip of the embolus; **6) **– embolus: in* Chrysilla* the embolus proper (ep) is thin and filiform as in *Phintelloides*; in *Phintella* and *Proszynskia* the ep is short and sclerotized, rigid and conical or acuminate, never filiform; in *Siler* the embolus is conical; **7) **– embolar tegular branch (etb) is present in males of *Chrysilla* and in *Phintelloidesscandens* and most probably also in the *Phintelloides* species from India and Sri Lanka; it is long, slender and flexible; the tegulum is like a fingerless glove with movable thumb; in *Phintella*, *Proszynskia* and *Siler*, the etb is absent, t­he embolus-bearing part not separate, the tegulum is rigid like a trowel **8) **– tegular lobe (pl) and tegular bump: in *Chrysilla* and *Phintelloides* the lobe is broad and rounded (Fig. 1), or shallow as in *P.scandens* (Fig. 14a, b, c, d, e); in *Phintella* and *Siler*it is triangular/funnel-shaped; in *Proszynkia* it is expanded and broadly rounded; all species have a bump on the tegulum, traditionally present in all chrysillines, usually in the middle or in the proximal half of the tegulum; **9) **– palpal colour: in *Chrysilla*, palp segments are contrasting, with different segments exhibiting different combinations of dark and white. The dark segments look iridescent blue or black in photos of live specimens; this seems to be a­ reliable character for species identification. In *Phintella* and *Phintelloide*s, palps are uniformly coloured, or all pale with dark cymbium; in *Silersemiglaucus* specimens, the femur, patella and tibia have various shades of buff or grey (in life probably blue or green), the cymbium is pure white as in *Chrysilla*;** 10) **– epigynal structure: *Chrysilla* and *Phintelloides* have a similar structure, deviating from all related genera: the lateral copulatory opening is vaguely defined (except in *C.deelemani*), the entrance section to the copulatory ducts is a large atrium, usually funnel-shaped like an opened birds beak, leading through a conspicuous, U-turn section with swollen parietal walls (bnc) (not swollen in *C.deelemani* and *C.scandens*) in transition to the vertical section of the copulatory ducts (cd) which are tubular and rigid. In *Phintella*, the copulatory opening is normally marked with a rigid ring, usually but not always positioned anteriorly (Kanesharatnam and Benjamin 2019: figs 33F, 36C). In *Chrysilla*, the middle, U-shaped section is often described as a birds’ neck (bnc), with a beak (atrium); spermathecae are situated near the posterior edge of the epigyne, they are round or reniform as in *Phintella* and relatively small; in *Phintella* the ducts are straight or curved and of various lengths; in *Siler* the copulatory opening is hidden in an anterior hood, the copulatory ducts are very short. The posterior epigynal margin is chitinized and provided with a pair of shallow pockets in most chrysilline genera.

#### Distribution

Seven *Chrysilla* species have been recorded from South and Southeast Asia with specimen records from the following countries: Sri Lanka, India, Pakistan, Nepal, Bhutan, Myanmar, Thailand, Vietnam, Malaysia, Singapore, Taiwan, Indonesia and southwest China. In addition, two species are recorded from tropical Africa, and one from Australia.

#### Taxon discussion

*Chrysilla* species are sexually dimorphic, and preserved specimens appear substantially different compared to living animals. This led to much confusion about the identity of the genus. It was more than a century after the first description of *Chrysilla* that males and females were associated (Caleb et al. 2018, Wang and Zhang 2012). Live animals of the different sexes exhibit different patterns and colours in both carapace and abdomen (Fig. 2; Caleb et al. 2018, Koh et al. 2022, Wang and Zhang 2012). Ten species are currently catalogued (World Spider Catalog 2024); with the description herein of the previously unknown female of *C.deelemani* Prószyński & Deeleman-Reinhold, 2010, five *Chrysilla* species are known from only one sex. *Chrysilla* *doriae* Thorell, 1890 (male, Sumatra), has a palp which is typical for *Phintella* species and the species probably is a synonym (CDR personal observation of holotype). The holotype of *Chrysilla delicata *Thorell 1892 (female, Sumatra; not Myanmar, contra World Spider Catalog 2024) was very recognisably illustrated (Prószyński 1984: 69), together with the palp of a syntopic male "*Iciusglaucochira* Thorell, 1890." Later, the male *Phintellaconradi* Prószyński and Deeleman-Reinhold 2012 was described from another (but likely conspecific) male specimen from Sumatra. Recently, Kanesharatnam and Benjamin (2019) established *Chrysillajesudasi* Caleb & Mathai, 2014 as the type species of the new genus *Phintelloides*.

*Chrysilla*in many ways resembles and has been repeatedly confused with *Phintella* Strand, 1906 (Żabka 1985). A series of phylogenetic analyses of chrysilline salticids found *Phintella* and *Phintelloides* to be closely related, possibly in a clade with *Proszynskia* and *Icius*; *Chrysilla* is somewhat distantly related from these genera, and more closely related to *Siler* (Kanesharatnam and Benjamin 2019). Conflict within the Kanesharatnam and Benjamin (2019) study derives from analytical permutations of morphological and DNA sequence data under parsimony and likelihood optimality criteria. The absence of conspicuous bright colours makes species of *Phintelloides* look superficially like *Phintella* species. Nevertheless, the morphological and functional copulatory characters are substantially similar in *Chrysilla* and *Phintelloides*, and distinct from those in genera such as *Phintella*and *Proszynskia.*

Our diagnosis can be expressed in simple words: genus *Chrysilla* and *Phintelloides* share their reproductive engine (copulatory organs) but are enveloped in a different coat; black, white and yellow setae in *Phintelloides*, red body colour in life and iridescent scales with black and white in *Chrysilla*. Involving more chrysilline genera: the “*Chrysilla* coat” is more widespread and also is characteristic for other chrysilline genera, such as *Siler*, *Cosmophasis* and *Orsima*, whereas the specialised “*Chrysilla* engine” is shared between *Chrysilla* and *Phintelloides*, but remarkably is also present in the genus *Bristowia,* a genus Maddison (2015) provisionally placed in the *Hasariini*.

### Chrysilla lauta

Thorell, 1887

8F06576C-F835-5EA0-BC67-BE0E95FBD9C6

https://www.checklistbank.org/dataset/288943/taxon/5YKJJ

World Spider Catalog: urn:lsid:nmbe.ch:spidersp:032753
*Chrysillalauta* Thorell, 1887 - Thorell 1887: 378 (spider catalogue of Roewer 1955 erroneously cites p. 387) (m), type locality: Bhamo, Myanmar; Prószyński 1976: 154, fig. 237 (m); Prószyński 1983a: 44, figs 4-6 (m); Żabka 1985: 210, figs 81-82 (m) Vietnam (synonymy with *Cosmophasislongiventris*); Koh 1989: 103 (m, photo) Singapore; Song and Chai 1991: 14, fig. 2 (m) Hainan; Song et al. 1999: 507, fig. 290N-O (m) Hainan, Myanmar, Vietnam; *Prószyński and Deeleman-Reinhold 2010*: figs 36-37 (m); Koh and Ming 2013: 180 (m, photo); Prószyński 2018: fig. 5G (m); Yamasaki et al. 2018: 27, figs 2-24 (mf) Taiwan; Kanesharatnam and Benjamin 2019: 46, figs. 19A-E, 20A, B (mf) Sri Lanka; Koh and Bay 2019: 216 (m, photo); Peng 2020: 75 fig. 35a-b (m); Koh et al. 2022: 347 (mf) Singapore.
*Cosmophasislongiventris* Simon, 1903 - Simon 1903a: 732 (m) Sri Lanka (Ceylon), Philippines.

#### Materials

- **Type status:** Other material. **Occurrence:** catalogNumber: MMUE G7572.5441; occurrenceRemarks: labeled “blue and red”; recordedBy: F. & J. A. Murphy; individualCount: 1; sex: male; lifeStage: adult; otherCatalogNumbers: https://doi.org/10.3535/G0G-G7D-N5J; occurrenceID: 52F7782C-1297-59F2-95B3-032DD4842E7A; **Taxon:** scientificName: Chrysillalauta; **Location:** country: Singapore; locality: Kent Ridge; verbatimCoordinates: 1°17’N 103°47’E; decimalLatitude: 1.2833333333333; decimalLongitude: 103.78333333333; **Event:** eventDate: 1986-07-06; habitat: garden; **Record Level:** institutionID: https://ror.org/027m9bs27; institutionCode: MMUE; basisOfRecord: PreservedSpecimen
- **Type status:** Other material. **Occurrence:** catalogNumber: MMUE G7572.6430; recordNumber: DSC 6285-6591; recordedBy: F. & J. A. Murphy; individualCount: 1; sex: male; lifeStage: adult; otherCatalogNumbers: https://doi.org/10.3535/PER-LNE-HEW; occurrenceID: EA93CB0B-5889-5F48-A7F4-8DF6D466C5A9; **Taxon:** scientificName: Chrysillalauta; **Location:** country: Singapore; locality: Pulau Ubin; verbatimCoordinates: 1°25’N 103°57’E; decimalLatitude: 1.4166666666667; decimalLongitude: 103.95; **Event:** eventDate: 1991-01-27; habitat: roadside; **Record Level:** institutionID: https://ror.org/027m9bs27; institutionCode: MMUE; basisOfRecord: PreservedSpecimen
- **Type status:** Other material. **Occurrence:** catalogNumber: MMUE G7572.6440; recordNumber: DSC 3727-36; recordedBy: F. & J. A. Murphy; individualCount: 1; sex: male; lifeStage: adult; otherCatalogNumbers: https://doi.org/10.3535/HS2-8W8-F23; occurrenceID: 5C4F929A-3E03-5EE2-8D8A-513EBF473EA3; **Taxon:** scientificName: Chrysillalauta; **Location:** country: Malaysia; stateProvince: Selangor; locality: Banting; verbatimElevation: 100 m; verbatimCoordinates: 2°48’04”N 101°30’46”E; decimalLatitude: 2.8011111111111; decimalLongitude: 101.51277777778; **Event:** eventDate: 1982-12-17; **Record Level:** institutionID: https://ror.org/027m9bs27; institutionCode: MMUE; basisOfRecord: PreservedSpecimen
- **Type status:** Other material. **Occurrence:** recordNumber: CM 19182; recordedBy: F. & J. A. Murphy; individualCount: 1; sex: female; lifeStage: adult; otherCatalogNumbers: https://doi.org/10.3535/SGZ-EFZ-VRK; occurrenceID: 8DF23168-B93F-546A-AE23-EF2321DE0C54; **Taxon:** scientificName: Chrysillalauta; **Location:** country: Malaysia; stateProvince: Pahang; locality: Genting; verbatimCoordinates: 3°24’N 101°46’E; decimalLatitude: 3.4; decimalLongitude: 101.76666666667; **Event:** eventDate: 1990-12-08; **Record Level:** institutionID: https://ror.org/027m9bs27; institutionCode: MMUE; basisOfRecord: PreservedSpecimen

#### Description

**Male.** Total length from Singapore 7.1 mm and 4.2 mm, from Banting 4.3 mm. For differences with *C.volupe*, see under that species. Colouring of carapace, abdomen and palps see chapter “coloration in various chrysilline species”. Carapace with all red areas in live specimens bare and lacking setae and scales, remains of small iridescent particles visible on the anterior transverse bars. Chelicerae slanting, divergent in the large male, parallel in both smaller males (Fig. 3b). Legs I longest, dark with white contrasting band apically on tibia, other legs pale, femur I dorsally with some long thin white setae in alcohol, violet in life, leg IV with some dark rings. Abdomen long, thin and shiny and gradually tapering. In all 3 available males, dorsum black and shiny, central band with round iridescent scales uninterrupted all through, at 2/3 of the length with a bell-shaped area with small round white iridescent scales; sides with a pair of narrow strips bearing small round green reflecting scales; venter pale brown, a median pale band bordered by a pair of dark bands. Coxae dorsally with white flattened setae. Palps (Fig. 1), tibia and cymbium pale, strongly contrasting with dark femur and patella (blue in life). Proximal part of tegulum with ventral bulge (Fig. 1a: tb) and a tegular bump (Fig. 1a: tbu) as in *C.volupe*. Embolar tegular branch (Fig. 1a: etb) narrow and elongate, flexible, proximally articulating at basal, hidden part of tegulum (cf. Fig. 1c: beb), partly running alongside tegulum and projecting distally beyond retrolateral part of tegulum containing the sperm duct loop over distance p (Fig. 1a: p); embolus filiform, slightly flexed at base, length same as p.

Measurements (Singapore: Kent Ridge). Body length 7.10. Carapace 2.70 long, 1.80 wide*,* 1.15 high. Abdomen 4.30 long, 1.20 wide*.* Leg I 7.70 (2.50 [0.70 wide] – 1.10 – 1.90 – 1.50 – 0.70), leg II 5.20 (1.60 [0.45 wide] – 0.80 – 1.20 – 1.00 – 0.60) leg III 5.00 (1.50 – 0.70 – 1.00 – 1.30 – 0.50) leg IV 6.60 (1.80– 0.70 – 1.60 – 1.70 – 0.80). Palp 0.9 – 0.5 – 0.5 – 0.7 width cymbium 0.3.

**Female **(Genting). Colour photos of live females (Yamasaki et al. 2018: fig. 16, Koh et al. 2022: 347) show parts of carapace covered with white setae and abdomen with pattern of red, white, black and iridescent greenish spots with considerable variation between specimens. In the only female specimen available to us (Fig. 3c), iridescent scales as seen on carapace of males are lacking and green colour is lost. Chelicerae with parallel sides, promargin with one distal tooth, retromargin with two distal teeth. Legs all pale. Abdomen with few iridescent scales suggesting a vague dorsal pattern, lateral patch consisting of reddish procumbent hair, posteriorly a pattern of dark areas covered with simple black setae as in males, these are visible in several reversed V- arranged bars on posterior half of abdomen; venter as in males. Epigyne similar to that in *Phintelloidesjesudasi*, except spermathecae not touching and halfway twist in copulatory ducts as in *P.jesudasi,* absent in *C.lauta*. 

Measurements. Body length 5.0. Carapace 1.90 long, 1.30 wide*,* 0.95 high. Abdomen 3.00 long, 2.00 wide*, *0.80 high. Measurements of legs: I 4.05 [1.40 (0.40 wide]– 0.60 – 1,00 - 0.65 – 0.40) leg II 3.00 (1.00 [0.30 wide]– 0.50 – 0.70 – 0.50 -0.30), leg III 3.30 (0.90 – 0.50 – 0.70 – 0.50 – 0.70) leg IV 4.20 (1.20 – 0.50 – 1.00 – 0.90– 0.60).

#### Distribution

**Figure 6.**
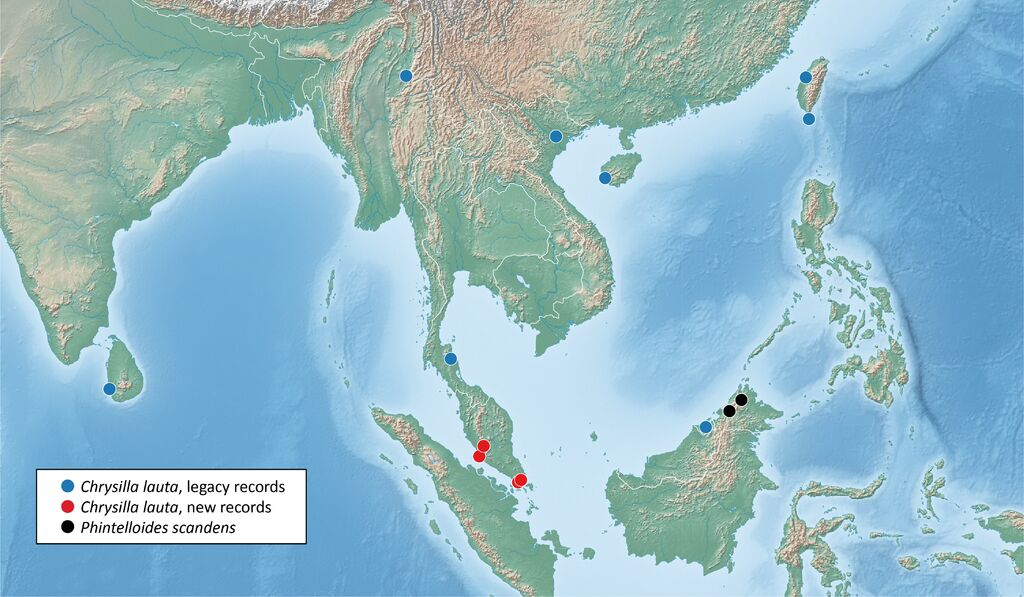
Map showing occurrence records for selected species. *Chrysillalauta* Thorell, 1887: blue circle for previously published records, red circle for new records; *Phintelloidesscandens* sp. nov.: black circles.

The species has been cited from Myanmar, Vietnam, Singapore, Thailand, Borneo, Taiwan, China and Sri Lanka (Fig. 6). The type locality Bhamo, Myanmar is situated close to the western border of southern Yunnan in a large mountain massive/complex across the Burmese - Chinese border, about 60 km from the Xishuangbanna tropical Botanical garden. This area has been extensively explored for spiders in the first decade of the 21th century in primary and various kinds of disturbed forest. Unfortunately, no *Chrysilla* species have been reported from that project.

#### Ecology

In forests and gardens, usually by beating/sweeping shrub and trees, from lowland up to 600 m.

#### Notes

The contrasting colouring of the male palps: dark femur and patella, pale tibia and cymbium was already mentioned by Thorell, 1887 in the description of the type specimen from Bhamo in northeastern Myanmar. It is consistently present in the material studied for the present paper.

### Chrysilla volupe

(Karsch, 1879)

1AFC737F-365F-59AD-82A9-C4BE43F6FDA2

https://www.checklistbank.org/dataset/288943/taxon/5YKJD

World Spider Catalog: urn:lsid:nmbe.ch:spidersp:035559
*Attusvolupe* Karsch, 1879 - Karsch 1879: 552 (m) Sri Lanka (Ceylon).
*Chrysilla*sp. - Prószyński 1984: 19 (m) Bhutan (according to Żabka 1988: 465).
*Silersemiglaucus* (Simon, 1901) - Prószyński 1985: 73, figs 16-17 (f, misidentified according to Caleb et al. 2018: 144) Sri Lanka (Ceylon).
*Phintellavolupe* (Karsch, 1879) - Żabka 1988: 465, figs 122-125 (m); Caleb and Mathai 2014: 64, figs 15-23 (m) India.
*Chrysillavolupe* (Karsch, 1879) - Caleb 2016: 271, India; Thumar and Dholakia 2018: 2, figs 1-6 (m) India; Caleb et al. 2018: 144, figs 1-25 (mf) India; Kanesharatnam and Benjamin 2019: 49, figs 20C-F, 21A-E, 22A-D (mf) Sri Lanka; Magar et al. 2020: 4, figs 4-6 (mf) Nepal.

#### Materials

- **Type status:** Other material. **Occurrence:** catalogNumber: MMUE 7572.6434; recordNumber: CM 15916; recordedBy: F. & J. A. Murphy; individualCount: 1; sex: male; lifeStage: adult; otherCatalogNumbers: https://doi.org/10.3535/67X-9R9-YCM; occurrenceID: 01C98BCA-9D5C-5E2A-B63A-6417E1E0562B; **Taxon:** scientificName: Chrysillavolupe; **Location:** country: Sri Lanka; locality: Peradeniya, Leersia; verbatimElevation: 500 m; verbatimCoordinates: 7°16’01”N 80°35'44”E; decimalLatitude: 7.2669444444444; decimalLongitude: 80.595555555556; **Event:** eventDate: 1986-11-25; **Record Level:** institutionID: https://ror.org/027m9bs27; institutionCode: MMUE; basisOfRecord: PreservedSpecimen
- **Type status:** Other material. **Occurrence:** catalogNumber: RMNH.ARA.18249; recordedBy: P. R. & C. L. Deeleman; individualCount: 1; sex: male; lifeStage: adult; otherCatalogNumbers: https://doi.org/10.3535/6H9-R1R-330; occurrenceID: 1F561749-E7FA-5747-AA7A-FF9401CC24C8; **Taxon:** scientificName: Chrysillavolupe; **Location:** country: Sri Lanka; locality: Kataragama Peak (Tissamaharama); verbatimCoordinates: 6°23’35”N 81°20’17”E; decimalLatitude: 26.393055555556; decimalLongitude: 81.338055555556; **Event:** eventDate: 1981-08-18; habitat: dry bush litter; **Record Level:** institutionID: https://ror.org/0566bfb96; institutionCode: RMNH; basisOfRecord: PreservedSpecimen
- **Type status:** Other material. **Occurrence:** catalogNumber: RMNH.ARA.18259; recordedBy: P. R. & C. L. Deeleman; individualCount: 1; sex: female; lifeStage: adult; otherCatalogNumbers: https://doi.org/10.3535/WL8-0R1-42B; occurrenceID: C37E9FA4-7FA4-59B4-BF7A-7F5A705DC44B; **Taxon:** scientificName: Chrysillavolupe; **Location:** country: Sri Lanka; locality: Kataragama Peak (Tissamaharama); verbatimCoordinates: 6°23’35”N 81°20’17”E; decimalLatitude: 26.393055555556; decimalLongitude: 81.338055555556; **Event:** eventDate: 1981-08-18; habitat: dry bush litter; **Record Level:** institutionID: https://ror.org/0566bfb96; institutionCode: RMNH; basisOfRecord: PreservedSpecimen

#### Description

Additions to the description of the male (Leersia). Abdomen in alcohol with middle band slightly paler than lateral areas, as in *lauta* ornamented with some gold reflecting scales and anteriorly areas with black setae; venter as in *lauta*. All legs dark, tarsi mostly light. Male palp (Fig. 7): femur, patella and tibia and basal half of cymbium brown (blue in life), distal half of cymbium white. Measurements. Body length 3.40 (smaller then described specimens from India), carapace length 1.40, width 1.00, height 0.60. Abdomen length 2.00 width 0.55. Palp femur 0.60, patella 0.20, tibia 0.15, cymbium length 0,60, width 0.20. Chelicerae not diverging. Leg I 3.70 (1.10 [width 0.35] – 0.40 – 0.80 - 1.10 – 0.30), legs II lost, leg III 2.60 (0.70 – 0.40 - 0.50 – 0.60 – 0.40), leg IV 3.30 (1.00 - 0.30 – 0.70 – 0.90 – 0.40).

Female (Tissamaharama). No abdominal pattern distinguishable in preserved specimen (Fig. 8c; Kanesharatnam and Benjamin 2019: fig 22A). Live animals with mottled black, red, and iridescent blue (Kanesharatnam and Benjamin 2019: fig 22A; Caleb et al. 2018: figs 2, 4, 6, 8, 10, 12). Copulatory ducts longer than in *C.lauta*, length 1½ x diameter of spermatheca, in anterior half running adjacent and parallel to each other (Kanesharatnam and Benjamin 2019: fig. 20E, F); in *C.lauta* ducts length not much more than 1 diameter of spermathecae and curved over whole length (Fig. 4b, c, d).

#### Diagnosis

This species is similar to *C*. *lauta*. The carapace as in *C. lauta, *the dorsal abdomen pattern is distinctive, in life with an anterior iridescent green band followed by a wide M-shaped band in red, behind which another red band with green in between, distally an iridescent black/violet tail (Fig. 2, Caleb et al. 2018: figs 1-12); this pattern may be preserved or lost in alcohol. Male palp with several features that can be used for identification. In *C.volupe* (Fig. 7)*,* the palpal tibia and basal part of cymbium are darkish, (blue in life), white in *C.lauta*; the dorsal margin of rta is more slender and smoothly curved, in *C.lauta* it is somewhat wider and dorsally slightly undulating. This latter key character agrees with drawings of a palp of the type specimen of *C.volupe* from Sri Lanka by Żabka 1988 (figs 122, 124), but not when comparing with Prószyński's palp drawing of “*Chrysilla*” sp. from Bhutan (Prószyński 1984: 19), which was interpreted as this species by Żabka 1988: 466). In female *C. lauta* all legs are uniform pale, in female *C.volupe* legs are pale with a few black rings on leg IV.

#### Distribution

Sri Lanka, India, Bhutan, Nepal. Caleb and Mathai 2014 (p. 64) erroneously cite Burma among the distribution records (Caleb et al. 2018). In addition, the online biodiversity monitoring community iNaturalist.org has research grade records from Bangladesh and Myanmar. 

#### Ecology

Foliage and dry leaf litter.

#### Biology

Male *C.volupe* spiders have been seen moving their palps up and down continuously and waving their long thin abdomen in circles up in the air, exhibiting large white light-reflecting spots.

### Chrysilla deelemani

Prószyński & Deeleman-Reinhold, 2010

BC93DFED-4D0D-5AD6-B773-973AB0FE44A9

https://www.checklistbank.org/dataset/288943/taxon/5YKK7

World Spider Catalog: urn:lsid:nmbe.ch:spidersp:043594
*Chrysilladeelemani* Prószyński & Deeleman-Reinhold 2010 - Prószyński and Deeleman-Reinhold 2010: 159, figs 30-35 (m) Indonesia.

#### Materials

- **Type status:** Holotype. **Occurrence:** catalogNumber: RMNH.ARA.18264; recordedBy: S. Djojosudharmo; individualCount: 1; sex: male; lifeStage: adult; otherCatalogNumbers: https://doi.org/10.3535/VYQ-YW1-AGE; occurrenceID: F26693C8-F659-5D7C-BDCB-A4CBDDEB6EEB; **Taxon:** scientificName: Chrysilladeelemani; **Location:** island: Lesser Sunda Islands; country: Indonesia; locality: Lombok Island, Kuta; verbatimCoordinates: 8°52’S 116°17’E; decimalLatitude: -7.1333333333333; decimalLongitude: 116.28333333333; **Event:** eventDate: 1990-01-08/09; habitat: secondary forest, from foliage; **Record Level:** institutionID: https://ror.org/0566bfb96; institutionCode: RMNH; basisOfRecord: PreservedSpecimen
- **Type status:** Other material. **Occurrence:** catalogNumber: RMNH.ARA.18265; recordedBy: S. Djojosudharmo; individualCount: 3; sex: female; lifeStage: adult; otherCatalogNumbers: https://doi.org/10.3535/Z2J-WMP-FDH; occurrenceID: FA85005B-DC87-537F-A347-F1C0ADB2F377; **Taxon:** scientificName: Chrysilladeelemani; **Location:** island: Lesser Sunda Islands; country: Indonesia; locality: Lombok Island, Kuta; verbatimCoordinates: 8°52’S 116°17’E; decimalLatitude: -7.1333333333333; decimalLongitude: 116.28333333333; **Event:** eventDate: 1990-01-08/09; habitat: secondary forest, from foliage; **Record Level:** institutionID: https://ror.org/0566bfb96; institutionCode: RMNH; basisOfRecord: PreservedSpecimen

#### Description

**Male.** No photo exists of a live specimen. The ornamentation of the abdomen with bands and stripes of coloured scales in alcohol is basically similar as in the other *Chrysilla*. The species can be diagnosed by the long thin abdomen with dark dorsum with thin pale undulating strip in the middle, somewhat similar as in *lauta* and also present in females (Fig. 8d; Prószyński and Deeleman-Reinhold 2010: fig. 31), and the broad distally rounded tegulum*.* Carapace all brown, whole ocular area and rear part of thorax with small round bluish iridescent scales in both male and females, across anterior eye row and around AME a transverse band of white flattened setae and a patch of similar setae behind AME; white moustache b­elo­w the AME lacking in both male and female, marginal strip on thorax thin, marked with iridescent scales. Chelicerae in male slightly diverging distally. Leg I dark, leg II – III pale, leg IV pale with dark metatarsus. Abdomen thin and elongate, shiny and dark with a brown area on sort of dorsal scutum; central dorsal band and a pair of narrow lateral strips covered with green-violet iridescent scales, as in *Chrysillalauta.* Venter covered all over with greenish scales. Palp white except dark base of femur, origin of ep from etb obliquely prolateral (Fig. 9a). 

**Female.** Total lengths 3.20 - 3.40 mm. Dorsal pattern on carapace (Fig. 8d) with white flattened setae and tiny round green iridescent dots, predominantly on rear slope of thorax. Legs I-IV uniform pale. Abdomen brown with interrupted pale central band and a pair of parallel lateral strips (Fig. 8d) covered with elongate golden iridescent setae, rest of dorsum with dispersed white flattened setae; venter with short white hair. Epigyne (Fig. 9d) with slender copulatory ducts distally curved ventralwards showing copulatory openings; atrium short and wide, crescent-shaped, parallel to the anterior epigyneal margin.

**Measurements.** Male carapace 1.80 long, 1. 20 wide, abdomen 2.7 long, 0.95 wide. Legs lost. Palp femur 0.85, patella 0.24, tibia 0.26, cymbium 0.70, width cymbium. 0.20. Female, total body length 3.20, carapace 1.30 long, 1.0 wide, 0.6 high. Abdomen 1.5 long, 1.0 wide, epigyne 0.25 wide 0.25 high. Legs: femur I 0.8, femur II 0.7, femur III, 0.7 femur IV 0.9.

### Phintelloides

Kanesharatnam & Benjamin, 2019

C4D9ADA9-8DE8-508C-AA23-8AB34BFE5346

https://www.checklistbank.org/dataset/288943/taxon/6NMB

World Spider Catalog: urn:lsid:nmbe.ch:spidergen:04527
Phintelloides Kanesharatnam & Benjamin, 2019 - Kanesharatnam and Benjamin 2019: 20.
Chrysilla jesudasi (Caleb & Mathai, 2014)Kanesharatnam and Benjamin 2019: 20.

#### Diagnosis

Kanesharatnam and Benjamin 2019 (p. 22) diagnose the genus as follows: male with white tuft of flattened setae on the clypeus; white diamond-shaped mark behind the eye field; prosoma with pale yellow/white transverse band behind AME; abdomen with blackish or brownish grey, longitudinal median band bordered by pale yellow bands (or devoid of markings). In addition, they noted the presence of a comparatively long embolus in males; the apical portion of the bulbus with a lamellar process (although this is absent in some including *P.jesudasi*, *P.brunne*, and *P.scandens*, CLD); and the bird’s-neck-shaped diverging curves at anterior margin of epigynum. Furthermore, Phintelloides have white belt markings on the lateral prosoma, the leg I slightly robust in males, and male pedipalps featuring a small posterior lobe and a long RTA with a bent tip. Females have black patches on the eye field and surrounding PME, behind PLE, and on the posterior slope of the prosoma. In the female genitalia, CO oriented laterally outwards; CD medium-to-very long and bent or twisted; spermatheca pyriform or spherical.

We consider the above diagnosis difficult to interpret from a defining point of view. Several of the listed character states are not compared to that in related genera and some are not valid for all species. Diagnostic somatic characters for the genera involved can be found above in the diagnosis section for the genus *Chrysilla*, where different states of 10 main cognitive characters between *Chrysilla* and related genera are summarized. Here we restrict ourselves to adding a few aspects we consider useful. In female *Phintelloides* species, carapace pattern allegedly is distinctive viz. black and white pattern with 3 pairs of black eye spots (Fig. 10a, c). Indeed this character is found in females of *P.jesudasi*, *P.arborea* and *P.flavumi* but not in *P.brunne*, *P.flavoviri* or *P.orbisa, *or the species described below, *P.scandens* sp. nov. A similar pattern is also found in females of *Phintellapiatensis* Barrion and Litsinger 1995, and it possible that this species fits in the genus *Phintelloides.* In males, the basal part of etb seems to be attached dorsally and is hidden underneath the tegulum; this can only be ascertained by probing the palp manually. Epigyna with a bird’s neck curve (bnc) are, unlike any other salticid genus except *Chrysilla,* suggestive of a specialised copulatory system.

A “white moustache” turns up seemingly at random in various chrysilline genera and is inconvenient as a tool when identifying genera. In *Phintelloides* *scandens* sp. nov. males it is lacking (Fig. 15a), although females have a tuft of white setae in front of the AME (Fig. 15b).

*Phintelloidesversicolor* and *P.munita* are morphologically at the edge of the genus because the copulatory organs deviate from all other species by the following characters: the tegulum is undivided and distally bulgy and rigid, the prolateral margin is concave in ventral view; the tegular proximal lobe (pl) is broad and round, the filiform embolus is shorter than that in all known species of *Phintelloides* and at the base curved over 90°. Females of *>versicolor* and *munita* are distinct from other related species by the pair of characteristic black curled marks on white background on the rear part of the carapace (Fig. 17b, e). Furthermore, these females are quite distinct from those of *Phintelloides* and *Phintella* by the straight copulatory ducts directed anteriorly and, the absence of the bird’s-neck-shaped curves the absence of the atrium; the opening is connected to a single pair of transverse, horizontal hood-like folds running parallel to the anterior edge of the epigyne.

### Phintelloides flavumi

Kanesharatnam & Benjamin, 2019

0144CA91-EFD8-5A96-9CBA-BAA99FC79FA0

https://www.checklistbank.org/dataset/288943/taxon/76YQL

World Spider Catalog: urn:lsid:nmbe.ch:spidersp:051095
*Phintelloidesflavumi* Kanesharatnam & Benjamin, 2019 - Kanesharatnam and Benjamin 2019: 35, figs 3, 10A-D, 14A-F, 15A-E, 16A-D (mf) Sri Lanka.

#### Materials

- **Type status:** Other material. **Occurrence:** catalogNumber: RMNH.ARA.18250; recordedBy: P. R. & C. L. Deeleman; individualCount: 1; sex: female; lifeStage: adult; otherCatalogNumbers: https://doi.org/10.3535/B59-03B-FWV; occurrenceID: 3E2FBFE3-4F1C-54E0-AC09-B662B82EFF44; **Taxon:** scientificName: Phintelloidesflavumi; **Location:** country: Sri Lanka; locality: Rathnapura; verbatimCoordinates: 6°42'N 80°23'E; decimalLatitude: 6.7; decimalLongitude: 80.383333333333; **Event:** eventDate: 1981-08-22/23; habitat: forest below tennis club; **Record Level:** institutionID: https://ror.org/0566bfb96; institutionCode: RMNH; basisOfRecord: PreservedSpecimen

#### Description

**Additions to the description. Female.** The total length of our female is similar to that given for the type specimen, however the legs in our specimen are 50% shorter than the legs measured in the type specimen; the posterior legs are longer than the anterior legs unlike the type material. The underside of the abdomen has small iridescent scales, like *scandens*. Fig. 11a, c shows the vulva, cleared in clove oil, viewed from ventral and ventrolateral. The bird’s neck-shaped curve tilts in ventral direction. The funnel-shaped openings (Fig. 11c) are the atrium (a), the copulatory opening is at the bottom of the funnel. As in *Chrysilla*. the atrium is delineated by an upper and a lower projection of the duct wall suggesting an opened birds’ beak. In male *P.flavumi*, according the description the embolar tegular branch is shorter than the adjacent tegulum; this would agree with the concept that during copulation, the bird’s beak (the atrium) lodges the tip of the etb and swallows the embolus through the copulatory opening whereas the more anteriorly situated cavity on the inner birds’ neck curve (Fig. 11d: ibc) is spatially correctly situated to receive the tegular tip and at the same time fix the base on the posterior pocket (see sketch of *P.scandens, Fig. 1c*). Measurements. Total length 4.50, Carapace 2.0 long, 1.65 wide, 1.00 high. Abdomen 2.50 long. Legs: I 3.75 (1.15– 0.65 – 0.85 - 0.60 – 0.50), leg II 3.70 (1.15– 0.60 – 0.90 – 0.55 -0.50), leg III 4.30 (1.40 – 0.60 – 0.90 – 1.00 – 0.40), Leg IV 4.30 (1.50 – 0.55 – 0.85 – 0.90 – 0.50), palp 0.70 – 0.60 – 0.30 - 0.35. Epigyne 0.25 wide, 0.30 long.

### Phintelloides jesudasi

(Caleb & Mathai, 2014)

6B87D13E-9298-5CD3-9BC3-F93AD1C82780

https://www.checklistbank.org/dataset/288943/taxon/76Z2L

World Spider Catalog: urn:lsid:nmbe.ch:spidersp:047200
*Chrysillajesudasi* Caleb & Mathai, 2014 - Caleb and Mathai 2014: 63, figs 1-14 (mf) India.
*Phintelloidesjesudasi* (Caleb & Mathai, 2014) - Kanesharatnam and Benjamin 2019: 41, figs 3, 6E-H, 17A-E, 18A-D (mf) Sri Lanka; Caleb 2020: 15739, figs 17E-G, 29B (mf) India.

#### Materials

- **Type status:** Other material. **Occurrence:** catalogNumber: RMNH.ARA.18258; recordedBy: P. R. & C. L. Deeleman; individualCount: 1; sex: female; lifeStage: adult; otherCatalogNumbers: https://doi.org/10.3535/SVV-BR5-KGE; occurrenceID: EB5E12DA-2878-544A-BE8C-3B5C783FD0CE; **Taxon:** scientificName: Phintelloidesjesudasi; **Location:** country: Sri Lanka; locality: Tissamaharama, Kataragama Peak; verbatimCoordinates: 6°42'N 80°23'E; decimalLatitude: 6.3930555555556; decimalLongitude: 81.338055555556; **Event:** eventDate: 1981-08-18; habitat: dry bush litter; **Record Level:** institutionID: https://ror.org/0566bfb96; institutionCode: RMNH; basisOfRecord: PreservedSpecimen

#### Taxon discussion

Originally described by Caleb and Mathai (2014) as a species of *Chrysilla*, Kanesharatnam and Benjamin (2019) established the genus *Phintelloides*for this and a few simliar species with *jesudasi* as the type species. Phylogenetic analysis based on both morphological and molecular data generally supported the monophyly of this group, with the exception that *Phintelloidesversicolor* did not always cluster with the rest of the genus (see treatment of *Phintelloidesversicolor*). 

### Phintelloides scandens

Deeleman-Reinhold, Addink & Miller sp. nov.

FAE69C8F-621D-5BAC-9506-64383E8F0113

urn:lsid:zoobank.org:act:34E30429-650A-413C-A6B3-6313C14C5F5B

#### Materials

- **Type status:** Holotype. **Occurrence:** catalogNumber: RMNH.ARA.18251; recordedBy: A. Floren; individualCount: 1; sex: male; lifeStage: adult; otherCatalogNumbers: https://doi.org/10.3535/5SG-PLB-MHT; occurrenceID: CE2C0B1A-7A8D-5384-9A1F-59AC87EE29EA; **Taxon:** scientificName: Phintelloidesscandens; **Location:** island: Borneo; country: Malaysia; stateProvince: Sabah; locality: Mt. Kinabalu N. P., Sorinsim; verbatimElevation: 500-700 m; verbatimCoordinates: 6°5'N 116°50'E; decimalLatitude: 6.0833333333333; decimalLongitude: 116.83333333333; **Event:** samplingProtocol: fogging canopy Vitex pinnata (Verbenacae); eventDate: 1997-03-05/14; habitat: 40 year old secondary forest; fieldNotes: (Loc 57); **Record Level:** institutionID: https://ror.org/0566bfb96; institutionCode: RMNH; basisOfRecord: PreservedSpecimen
- **Type status:** Paratype. **Occurrence:** catalogNumber: RMNH.ARA.18252; recordedBy: A. Floren; individualCount: 1; sex: female; lifeStage: adult; otherCatalogNumbers: https://doi.org/10.3535/85R-G3E-4M0; occurrenceID: 2177F899-9F22-50D9-867C-50C57D8C6544; **Taxon:** scientificName: Phintelloidesscandens; **Location:** island: Borneo; country: Malaysia; stateProvince: Sabah; locality: Mt. Kinabalu N. P., Sorinsim; verbatimElevation: 500-700 m; verbatimCoordinates: 6°5'N 116°50'E; decimalLatitude: 6.0833333333333; decimalLongitude: 116.83333333333; **Event:** samplingProtocol: canopy fogging tree 8 Vitex pinnata (Verb.); eventDate: 1997-03-10; habitat: 15 year old secondary forest; fieldNotes: (Loc 46), refog 1 after 8 days; **Record Level:** institutionID: https://ror.org/0566bfb96; institutionCode: RMNH; basisOfRecord: PreservedSpecimen
- **Type status:** Paratype. **Occurrence:** catalogNumber: RMNH.ARA.18253; recordedBy: A. Floren; individualCount: 1; sex: female; lifeStage: adult; otherCatalogNumbers: https://doi.org/10.3535/7WH-VHP-M1K; occurrenceID: 1128E2B4-04D3-55A3-BB15-62B1C402FEA2; **Taxon:** scientificName: Phintelloidesscandens; **Location:** island: Borneo; country: Malaysia; stateProvince: Sabah; locality: Mt. Kinabalu N. P., Sorinsim; verbatimElevation: 500-700 m; verbatimCoordinates: 6°5'N 116°50'E; decimalLatitude: 6.0833333333333; decimalLongitude: 116.83333333333; **Event:** samplingProtocol: canopy fogging Vitex pinnata (Verb.); eventDate: 1997-02-26; habitat: 15 year old secondary forest; fieldNotes: (Loc 38, tree code Vp267); **Record Level:** institutionID: https://ror.org/0566bfb96; institutionCode: RMNH; basisOfRecord: PreservedSpecimen
- **Type status:** Other material. **Occurrence:** catalogNumber: RMNH.ARA.18254; recordedBy: A. Floren; individualCount: 1; sex: male; lifeStage: adult; otherCatalogNumbers: https://doi.org/10.3535/1CE-SXA-2BC; occurrenceID: 59A912D4-409B-50F8-B622-3F4579A0536F; **Taxon:** scientificName: Phintelloidesscandens; **Location:** island: Borneo; country: Malaysia; stateProvince: Sabah; locality: Crocker Range, near Keningau; verbatimCoordinates: 5°26’N 116°08’E; decimalLatitude: 5.4333333333333; decimalLongitude: 116.13333333333; **Event:** samplingProtocol: fogging canopy Melanopis (Euphorbiaceae); eventDate: 2001-02-19; habitat: 10 year old isolated secondary forest; fieldNotes: (CRI.9, tree code Me305, DSC 2286-88); **Record Level:** institutionID: https://ror.org/0566bfb96; institutionCode: RMNH; basisOfRecord: PreservedSpecimen
- **Type status:** Other material. **Occurrence:** catalogNumber: RMNH.ARA.18255; recordedBy: A. Floren; individualCount: 1; sex: male; lifeStage: adult; otherCatalogNumbers: https://doi.org/10.3535/0RA-FVV-2DL; occurrenceID: 2E93698A-6195-532B-B995-C54B919B114F; **Taxon:** scientificName: Phintelloidesscandens; **Location:** island: Borneo; country: Malaysia; stateProvince: Sabah; locality: Crocker Range, near Keningau; verbatimCoordinates: 5°26’N 116°08’E; decimalLatitude: 5.4333333333333; decimalLongitude: 116.13333333333; **Event:** samplingProtocol: fogging canopy Melanopis (Euphorbiaceae); eventDate: 2001-02-18; habitat: 20 year old isolated secondary forest; fieldNotes: (CRII.4, tree code Me310); **Record Level:** institutionID: https://ror.org/0566bfb96; institutionCode: RMNH; basisOfRecord: PreservedSpecimen
- **Type status:** Other material. **Occurrence:** catalogNumber: RMNH.ARA.18256; recordedBy: A. Floren; individualCount: 1; sex: male; lifeStage: adult; otherCatalogNumbers: https://doi.org/10.3535/SZV-FJV-MRM; occurrenceID: EDE8AAB1-77E1-5BC6-914F-ACBAF994BA57; **Taxon:** scientificName: Phintelloidesscandens; **Location:** island: Borneo; country: Malaysia; stateProvince: Sabah; locality: Crocker Range, near Keningau; verbatimCoordinates: 5°26’N 116°08’E; decimalLatitude: 5.4333333333333; decimalLongitude: 116.13333333333; **Event:** samplingProtocol: fogging canopy Melanopis (Euphorbiaceae); eventDate: 2001-02-18; habitat: 20 year old isolated secondary forest; fieldNotes: (CR II.3, DSC 1142-52, tree code Me309); **Record Level:** institutionID: https://ror.org/0566bfb96; institutionCode: RMNH; basisOfRecord: PreservedSpecimen
- **Type status:** Other material. **Occurrence:** catalogNumber: RMNH.ARA.18257; recordedBy: A. Floren; individualCount: 1; sex: female; lifeStage: adult; otherCatalogNumbers: https://doi.org/10.3535/FEE-JQY-GA4; occurrenceID: 768B340C-2DCC-5A68-928E-685939849112; **Taxon:** scientificName: Phintelloidesscandens; **Location:** island: Borneo; country: Malaysia; stateProvince: Sabah; locality: Crocker Range, near Keningau; verbatimCoordinates: 5°26’N 116°08’E; decimalLatitude: 5.4333333333333; decimalLongitude: 116.13333333333; **Event:** samplingProtocol: fogging canopy Melanopis (Euphorbiaceae); eventDate: 2001-02-18; habitat: 20 year old isolated secondary forest; fieldNotes: (CRII.5, DSC 1178-1185, treecode Me311); **Record Level:** institutionID: https://ror.org/0566bfb96; institutionCode: RMNH; basisOfRecord: PreservedSpecimen

#### Description

MALE. Total length males 4.30 - 5.40 mm. Holotype*: *carapace dark brown with white tuft between and behind AME eyes and black protruding setae hooding AM eyes (Fig. 15a), area between and behind PME and between AME and ALE covered with short white setae, intermitted with protruding brown setae; in posterior half of thorax centre a pale narrow longitudinal stripe (Fig. 12a) instead of a diamond-shaped white area in other known species. Posterior margin of thorax with appressed black setae, opposite front edge of abdomen bearing brushes of dark erect hair in the middle, white on sides as in females (Fig. 12a, c), reduced or lost in some specimens. Chelicerae long, divergent (Fig. 12b), medially slanting. Maxillae distally widened with angular tip and a toothlike sub-tip. Leg I with dark femur, tibia and metatarsus dark with one or more pale rings; legs II-IV pale with narrow dark rings around the joints and femora with prolateral dark lengthwise stripes, tibia I and II with 2 pairs of ventral spines, metatarsi I and II with one pair at base (Fig. 12a). Abdomen gradually tapering, dorsally with wide buff coloured central band (colour in live specimens unknown), covered with very small scales, on either side a wide lateral band covered with white scales (Fig. 12a); venter covered with big round to triangular buff to brown scales and flanked by a pale area with some sparce very small iridescent scales (Fig. 12b). Pedipalp with base of palpal femur dark, rest light, patella and tibia white, cymbium dorsally dark except base and tip (Fig. 14b). Tip of etb extends slightly beyond tip of sperm duct loop (Fig. 14a, c). Embolus relatively short (e.g., compared to *P.jesudasi, Kanesharatnam and Benjamin 2019*: fig. 18A). 

Measurements. Total length 4.80. Carapace 2.20 long, 1.50 wide, 1.10 high, chelicerae 0.8 long, abdomen 2.50 long, 1.10 wide*. *Legs: I 4.90 (1.50 long [0.40 wide]* – *0.70 – 1.20 – 0.90 – 0.60, leg II 3.80 (1.10 [0.30 wide] – 0.50 – 0.80 – 1.00 – 0.40) leg III 4.00 (1.20 [0.30 wide] – 0.50 – 0.80 – 0.90 – 0.60 leg IV 4.40 (1.40 [0.35 wide)]– 0.50 – 0.90 – 1.10 – 0.50. Palp -0.7 – 0.3 – 0.25 – 0.6, width cymbium 0.25*.*

**Figure 16.**
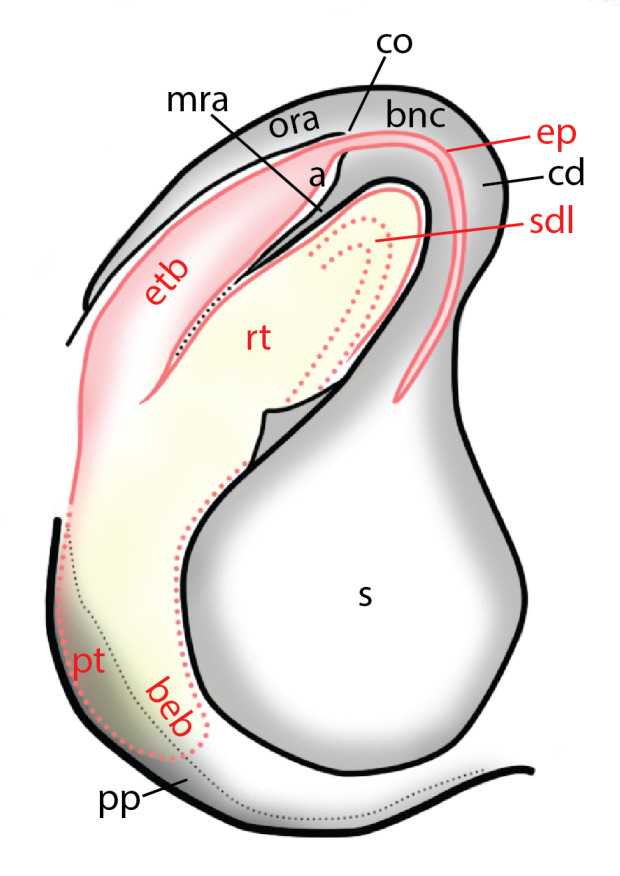
*Phintelloidesscandens* sp. nov., schematic illustrations showing hypothetical interaction between male and female genitalia** a** atrium **beb** base of embolar tegular branch **bnc** bird’s neck curve **cd** copulatory duct **co** copulatory opening **ep** embolus proper **etb** embolar tegular branch **mra** median rim of atrium **ora** outer rim of atrium **pp** posterior pockets **pt **proximal lobe of tegulum **rt **retrolateral lobe of tegulum **s** spermatheca **sdl **sperm duct loop

FEMALE. Paratype. Carapace paler than that of male, head pale, lacking white area in eye region, but with bunch of white setae on clypeus. Anterior eyes surrounded with white hair, a white moustache is present below the front eyes (Fig. 13c). present*. *Femur I with a prolateral and retrolateral dark longitudinal stripe, a prolateral one on leg II and III and none on femur IV (Fig. 13a). All legs with black rings at base and tip of the patella and tibia. Abdomen pale, dispersed with brownish scales, mostly on the sides (Fig. 13d). Venter uniform pale, very small iridescent scales (Fig. 13b). Palps uniform pale. Epigyne (Fig. 13) similar to *C.lauta* and *C.volupe*, spermathecae considerably larger, touching, “bird’s neck” short and thin and lacking a twist; outer margin (ora) of bird’s beak reaching down till 2/3 of spermathecae*. *In dorsal view, upturned fertilization ducts pointing to each other with acute tip behind are visible behind spermathecae (Fig. 13d). For hypothetical illustration of copulatory mechanics, see Fig. 16.

Measurements. Paratype. Total length 4.50. Carapace 1.90 long, 1.30 wide, 1.10 high, abdomen 2.75 long, 1.72 wide. 1.42 high*.* Legs*: *I 2.90 (0.90 [0.35 wide]*– *0.50 – 0.60 - 0.50 – 0.40*)* leg II 2.55 (0.80 ([0.25 wide] *–* 0.35 – 0.55* –* 0.45 – 0.40), leg III 3.00 (0.95 – 0.40 – 0.60 – 0.70 – 0.35) leg IV 3.80 (1.15 – 0.50 – 0.75 – 0.90 – 0.50)*.*

#### Diagnosis

Males of *P.scandens* differ from most *Phintelloides* species by the absence of a white tuft on the clypeus in front of the AME in males (Fig. 15a). The thorax in males has a narrow white V-shaped strip in the middle instead of a diamond-shaped white area in other species. The female carapace (Fig. 12c) has markings similar to the male and unlike the characteristic pattern of *P.jesudasi*. The male abdomen (Fig. 12a), has a wide dark median band on the abdomen between a pair of yellow lateral bands as in *P.jesudasi* and in *P.versicolor*. The female abdomen pattern is the reverse of that in males (Fig. 12c). The structure of the male and female reproductive organs are generally similar to those of *C.lauta* and *P.jesudasi. *The male can be distinguished by differences in the colour of palpal segments, and by the form of the etb, which is longer and more slender than in *P.jesudasi* and fairly uniform in width, running alongside the tegulum over most of its length (Fig. 14c); the embolus has the same length as that in *jesudasi*, the proximal tegular lobe is smaller. The female wears a narrow bunch of white setae on the clypeus (Fig. 15b); epigyne lacks the widening of the bird’s-neck, the atrium is smaller and narrow, the ora (outer rim) is clearly longer; the spermathecae are round and larger than in any other species of the genus, with a diameter equal to the length of the ducts (Fig. 13).

#### Etymology

From the Latin word *scandere*, to climb, referring to the fact that all known specimens were collected by fogging tree canopies.

#### Distribution

Known from two locations in Sabah Province, northern Borneo. In the Kinabalu area, recorded from secondary forests near Sorinsim adjacent to primary forest. At Keningau, in isolated disturbed young secondary forest patches 10 and 20 years old.

### Phintelloides versicolor

(C. L. Koch, 1846)

ED448511-2CFA-5EB5-9F2B-97C0F1B469D1

https://www.checklistbank.org/dataset/288943/taxon/76Z2N

World Spider Catalog: urn:lsid:nmbe.ch:spidersp:035557
*Plexippusversicolor* C. L. Koch, 1846 - Koch 1846: vol. 13: 103, fig. 1165 (m) Bintan [Bintang] Island, Indonesia.
*Attusversicolor* (C. L. Koch, 1846) - Walckenaer 1847: 426.
*Maeviapicta* C. L. Koch, 1848 - Koch 1848: vol 14: 72, fig. 1328 (f; juv m according to Thorell 1891) Bintan [Bintang] Island, Indonesia.
*Chrysillaversicolor* (C. L. Koch, 1846) - Thorell 1891: 117 (mf; synonymy with *Meviapicta*) Indonesia (Bintang, Sumatra), Malaysia (Pinang), Singapore; Workman and Workman 1894: 10, pl. 10 (mf) Indonesia (Pinang, Sumatra, Bintang), Singapore; Simon 1901: 544; Żabka 1985: 211, figs 83-96 (mf) Vietnam.
*Telamonialeucaspis* Simon, 1903 - Simon 1903b: 307 (m) Sumatra.** syn. nov.**; Prószyński 1978: 336, fig. 11 (m).
*Phintellaleucaspis* (Simon, 1903) - Bohdanowicz and Prószyński 1987: 112, figs 214-215 (m).
*Phintellaversicolor* (C. L. Koch, 1846) - Prószyński 1987: 152, 161 (in part).
*Phintelloidesversicolor* (C. L. Koch, 1846) - Kanesharatnam and Benjamin 2019: 22.

#### Materials

- **Type status:** Other material. **Occurrence:** recordedBy: W. Corley; individualCount: 2; sex: male; lifeStage: adult; otherCatalogNumbers: https://doi.org/10.3535/C69-M7K-VWC; occurrenceID: 451E13DE-7042-5932-A295-646E928CEC1F; **Taxon:** scientificName: Phintelloidesversicolor; **Location:** country: Malaysia; stateProvince: Selangor; locality: Banting; verbatimElevation: 100 m; verbatimCoordinates: 2°48’04”N 101°30’46”E; decimalLatitude: 2.8011111111111; decimalLongitude: 10.512777777778; **Event:** eventDate: 1983-01-28; fieldNotes: CM 21848, DSC 6302-6327; **Record Level:** institutionID: https://ror.org/027m9bs27; institutionCode: MMUE; basisOfRecord: PreservedSpecimen
- **Type status:** Other material. **Occurrence:** catalogNumber: MMUE G7572.6413; recordedBy: F. & J. A. Murphy; individualCount: 1; sex: female; lifeStage: adult; otherCatalogNumbers: https://doi.org/10.3535/3NW-1BX-8BK; occurrenceID: A0A5233F-918E-5755-AB82-D3EE60952018; **Taxon:** scientificName: Phintelloidesversicolor; **Location:** country: Singapore; locality: Lim Chu Kang; verbatimCoordinates: 1°26’N 103° 43’E; decimalLatitude: 1.4333333333333; decimalLongitude: 103.71666666667; **Event:** eventDate: 1991-01-28/29; fieldNotes: CM19264; **Record Level:** institutionID: https://ror.org/027m9bs27; institutionCode: MMUE; basisOfRecord: PreservedSpecimen
- **Type status:** Other material. **Occurrence:** catalogNumber: RMNH.ARA.18260; recordedBy: P. R. & C. L. Deeleman; individualCount: 1; sex: female; lifeStage: adult; otherCatalogNumbers: https://doi.org/10.3535/M42-Z4P-DRD; occurrenceID: DE44414C-2104-59CD-9533-5324F9DABE02; **Taxon:** scientificName: Phintelloidesversicolor; **Location:** country: Thailand; stateProvince: Kanchanaburi Province; locality: Erawan waterfalls N. P.; verbatimCoordinates: 14°22’N 99°08’E; decimalLatitude: 14.366666666667; decimalLongitude: 99.133333333333; **Event:** eventDate: 1987-11; **Record Level:** institutionID: https://ror.org/0566bfb96; institutionCode: RMNH; basisOfRecord: PreservedSpecimen
- **Type status:** Other material. **Occurrence:** catalogNumber: RMNH.ARA.18261; recordedBy: P. R. & C. L. Deeleman; individualCount: 1; sex: male; lifeStage: adult; otherCatalogNumbers: https://doi.org/10.3535/5MR-J6N-26M; occurrenceID: 41A8211A-6F67-5ED3-9CA4-3F4C6ECADB9D; **Taxon:** scientificName: Phintelloidesversicolor; **Location:** country: Thailand; stateProvince: Prachuap Khiri Kan Province; locality: Sam Roi Yot National Park; verbatimCoordinates: 12°14’N 99°56’E; decimalLatitude: 12.233333333333; decimalLongitude: 99.933333333333; **Event:** eventDate: 1988-12-31; habitat: forest on limestone; **Record Level:** institutionID: https://ror.org/0566bfb96; institutionCode: RMNH; basisOfRecord: PreservedSpecimen
- **Type status:** Other material. **Occurrence:** catalogNumber: RMNH.ARA.18262; recordedBy: P. Schwendinger; individualCount: 1; sex: male; lifeStage: adult; otherCatalogNumbers: https://doi.org/10.3535/Q6C-91C-BS5; occurrenceID: 5EEB6839-2EC9-54A3-96FA-70DE59BC8142; **Taxon:** scientificName: Phintelloidesversicolor; **Location:** country: Thailand; locality: Chiang Mai; verbatimElevation: 300 m; verbatimCoordinates: 18°47’N 98°57’E; decimalLatitude: 18.783333333333; decimalLongitude: 98.95; **Event:** eventDate: 1987-07-01; **Record Level:** institutionID: https://ror.org/0566bfb96; institutionCode: RMNH; basisOfRecord: PreservedSpecimen

#### Description

Both male and female with flattened white hair on clypeus, in males just a small moustache below AME (Fig. 17c), in females with frontal strip of thick white flattened setae over whole carapace width (Fig. 17d); anterior eye region with patch covered with white setae, thorax with wide broad submarginal band with dark edge (Fig. 17a), in live specimens black with 2 white central patches and several small ones (Koh et al. 2022: 437). In alcohol tiny greenish iridescent pits on head in male and female. In males, legs I dark, with a light ring on tibia, metatarsus and tarsus, other legs pale; in females, legs and palps pale (all these features are also mentioned in the original description of *leucaspis* by Simon 1903b, here synonymized with *versicolor*). Abdomen dorsally with elongate black and white scales, side all white, venter in both sexes partly covered with white appressed flattened setae. Male palp pigmented on trochanter and base of femur, rest white; female palps all white. Epigyne of female (from Thailand) with slender, almost straight ducts (Fig. 18c, e).

Measurements. Total length: males Banting 6.30 and 4.40, males (Sam Roi Yot N. P.) 4.70 in mm, , Chiang Mai 5.00. Male Sam Roi Yot: total length 4.70, carapace 2.30 long, 1.80 wide 1.30 high, abdomen 2.30 long, 1.20 wide; palp 0.80 – 0.35 – 0.30 - 0.60, width cymbium 0.23.

#### Diagnosis

The abdomen in males is easily recognizable by the dark central band flanked by a pair of lateral white bands (yellow in life; Fig. 17a, Koh et al. 2022: 437), in reverse to that in most *Chrysilla* and *Phintella* species and similar to *Phintelloidesscandens*; this is a reliable character also valid in material preserved in alcohol. This feature apparently is expressed in the latin name: reversal of pale and dark. The shape of the white central area on the thorax is variable in shape and width (compare Fig. 17a from Thailand with Koh et al. 2022: 437 from Singapore). Just like in representatives of *Chrysilla* and *Phintelloides*, the embolus is filiform and relatively short, straight and then slightly curved and bent near the base at an angle of 90° with the retrolateral distal edge of the tegulum (Fig. 19a). For a difference in tegulum see diagnosis of *munita.* Females differ from males by the different carapace, having a pair of black semi-rings on a light background on the posterior part of the thorax; they differ in abdomen pattern which is dorsally pale with irregular cinnamon-brown blotches and a central white band (Fig. 17b; Koh et al. 2022: 437). Epigyne (Fig. 18a, c, e): the copulatory duct is uniform in diameter, parallel, at the anterior end the ring-like copulatory opening in a 90° inward bend, the outer edge is prolonged as a fold or rim; the left and righthand folds are directed mesally, relatively short, the tips crossing. See *P.munita* for differences with that species. 

#### Distribution

Sumatra, Bintang Island, Singapore, Malaysia, Thailand, Brunei, Vietnam.

#### Taxon discussion

The previous generic assignment to *Phintella* of this species is doubtful, as the embolus does not conform to the definition of that genus (see for example Żabka 1985: fig. 403), nor of *Phintelloides* (see for example Kanesharatnam and Benjamin 2019: fig. 18A). But despite the generic ambiguity, *Phintelloidesversicolor* is difficult to distinguish from its close relative *Phintelloidesminuta, *particularly based on the male copulatory organ. *Phintelloidesminuta* was removed from synonymy with *P.versicolor* without argumentation (Prószyński 2016; https://salticidae.pl/salticidae.php?adres=specimen.php?id=12129), and in lieu of justification this has not been adopted by World Spider Catalog (2024). However, we agree with the validity of both species and shall try to provide the missing arguments. 

This species complex is a taxonomic snake in the grass. In the various papers listed, the identity of this species is full of contradictions. Prószyński (2017), in his pragmatic classification, used this species as the representative of the genus *Phintella*; this is misleading*.* The World Spider Catalog (2024) cites 62 taxonomic treatments of *P.versicolor*. The species including its synonyms has been placed in 11 different genera over nearly 175 years. Recently, it was assigned to *Phintelloide*s (Kanesharatnam and Benjamin 2019, p. 22). The genus assignment of *versicolor* through 130 years has commuted between *Chrysilla* or *Phintella* by authors with authority (Simon, Zabka, Song, Prószyński) which suggests that the species fits in neither of them satisfactorily. 

Koch’s description of the male from a small islant between Singapore and Sumatra is mostly an enumeration of colours of the various body parts: black, white, and yellow, and the central abdominal band rusty red, which also fits our specimens. He mentioned that the female is unknown, but described one two years later as *Maeviapicta* from the same locality. Then, starting in the 1970s, records attributed to this poorly known tropical species stated to appear from Japanese localities on the latitude of southern Europe (Yaginuma 1977, Prószyński 1973). Prószyński (1973) was the first to provide detailed drawings of the male’s genital organs, in a paper on salticid type specimens from Japan present in the Berlin Museum. He stated that he found the male type specimen of Koch’s *“Plexippus” versicolor* from Bintang Island as well as male and female specimens labeled *Hasariusversicolor* Koch from Japan (the latter name combination does not appear elsewhere in the taxononmic literature; World Spider Catalog 2024). In the description, Prószyński (1973) focused on the colouration and the abdomen pattern and apparently decided for some reason that the specimens from Sumatra and Japan are conspecific. No female was available from Bintang. Genitals, male and female, he drew from Japanese specimens only (Prószyński 1973, figs 1-7).

Twelve years later, in the magnificent work by Żabka (1985) on Salticidae from Vietnam appear excellent drawings of a male palp by Prószyński of the alleged holotype of *versicolor* (Żabka 1985, figs 91, 92) side by side with Zabka’s drawings of *versicolor* from the same specimen, but apparently opposite pedipalp; Żabka 1985, figs 88-90) along with a specimen from Vietnam (Żabka 1985, figs 83-86). Prószyński’s 1973 identification of *versicolor* from Japan was followed by Yaginuma (1977) and since then a number of authors cited, re-described and illustrated males and females of *versicolo*r from various material from Japan and China. It has to be admitted that the morphology of palps from the Malay and Japanese specimens is very similar, and warrants further comparative study. However, as is the case also in certain other chrysilloid genera, it is the females that express their identity more clearly than do males by differences in structure in the epigyne. The drawings of the epigyne from Vietnam (Żabka 1985, figs 93-95) differ consistently from those from the Japanese specimens, and better agree with that from specimens we collected in Malaysia and Thailand, representing *versicolor*. In Bohdanowicz and Prószyński (1987), the latter author presented illustrations of the palp of *Phintellaleucaspis* (Simon) from Sumatra (figs 214, 215), which looks identical to drawings of tropical Southeast Asian *versicolor *specimens and apparently *leucaspis* is a new synonym of *versicolor.* Although Japanese specimens according to drawings of palpal structure can hardly be distinguished from that of specimens from Bintang Island, Sumatra, Malaysia, Thailand and Vietnam, the epigynes drawn from Japan, China and Hong Kong (e.g., Fig. 18b, d, f; Zhu and Zhang 2011, fig. 362a, b; Prószyński 1973, figs 6, 7) are incompatible with female specimens from the Malay Region, which have not been figured in detail previously (Fig. 18a, c, e). The population represented in Japan and China cannot be maintained in *versicolor*; the oldest name available is *munitus* Boesenberg & Strand 1906, which name we propose to remove from synonymy.

#### Notes

The World Spider Catalog (2024) erroneously lists *Maeviapicta* as *Maeviapicta* C. L. Koch 1846: 72; it should be C. L. Koch 1848: 72 (Brignoli 1985).

### Phintelloides minuta

(Bösenberg & Strand, 1906)

A4638846-5917-53DC-A786-E3864FB52537

zoobank.org/NomenclaturalActs/34E30429-650A-413C-A6B3-6313C14C5F5B

**Removed from synonymy with** ***Phintelloidesversicolor***
*Jotusmunitus* Bösenberg & Strand, 1906 - Bösenberg and Strand 1906: 334, pl. 14 fig. 374 (f) Japan; (m, plate 14 fig. 392, see *Phintellalinea* (Karsch, 1879), Prószyński 1987: 161); Yaginuma 1955: 14; Yaginuma 1960: 107, fig. 89.5 (f) Japan; Lee 1966: 55, fig. 28d-f (f) Taiwan; Yaginuma 1971: 107, fig. 89.5 (f) Japan; Yin and Wang 1979: 32, fig. 13A-E; Hu 1984: 370, fig. 386.1-6.
*Chira albiocciput *Bösenberg & Strand, 1906 - Bösenberg and Strand 1906: 366, pl. 13, fig. 311 (m) Japan.
*Aelurillusdimorphus* Dönitz & Strand, in Bösenberg and Strand 1906: 398, pl. 9, figs 125-126 (mf) Japan; Saitō 1959: 147. fig 203a-e.
*Jotusmunitus chinesicus *Strand, 1907 - Strand 1907: 569 (f) China.
*Dexippusdavidi* Schenkel, 1963 - Schenkel 1963: 446, fig. 255a-e (m) China.
*Dexippustschekiangensis* Schenkel, 1963 - Schenkel 1963: 449, fig. 256a-e (f) China.
*Chrysillaversicolor* (C. L. Koch, 1846) - Prószyński 1973: 98, figs 1-7 (mf) Japan; Yaginuma 1977: 398, Japan (synonymy with *Jotusmunitus*); Song 1982: 102 (synonymy with *Dexippusdavidi*).
*Icius munitus* (Bösenberg & Strand, 1906) - Wesołowska 1981a: 59, figs 34-36 (f) North Korea.
*Icius tschekiangensis* (Schenkel, 1963) - Wesołowska 1981b: 135, figs 27-30 (f) China.
*Phintella davidi* (Schenkel, 1963) - Prószyński 1983b: 6.
*Phintella tschekiangensis* (Schenkel, 1963) -*Prószyński 1983b*: 7.
*Phintellaversicolor* (C. L. Koch, 1846) - *Prószyński 1983a*: 44, fig. 3 (m) Japan; Prószyński 1983b: 6; : 231, f. 129.1 (mf); Yaginuma 1986: 161; Song 1987: 288 (mf); Maddison 1987: 103, figs 7-8; Prószyński 1987: 152, 161 (in part); Bohdanowicz and Prószyński 1987: 113, figs 210-213, 216-221 (mf) (partim); Matsumoto 1989: 125, fig. 1E, J (m); Chikuni 1989: 149, fig. 12 (mf); Feng 1990: 202, fig. 177.1-4; Chen and Gao 1990: 191, fig. 243a-b (mf); Chen and Zhang 1991: 290, fig 303 (mf); Peng et al. 1993: 162, figs 569-576 (mf); Zhao 1993: 411, fig. 212a-c (mf); Zhao 1995: 1128, fig. 553a-c (mf); Maddison 1996: 330, fig. 17 (m); Song et al. 1997: 1738, fig. 50a-c (f); Song et al. 1999: 539, figs 308O-P, 309F-G, 328E-F (mf); Hu 2001: 403, fig. 256.1-3 (m; correction, see notes below); Namkung 2002: 616, fig. 43.60a-c (mf) South Korea; Cho and Kim 2002: 120, figs 58, 163-164, 272-273 (mf); Namkung 2003: 620, fig. 43.60 (mf); Ono et al. 2009: 572, figs 140-143 (mf); Zhu and Zhang 2011: 497, fig. 362A-D (mf); Yin et al. 2012: 1429, fig. 779a-h (mf); Kim and Lee 2014: 113, fig. 81A-E (mf); Prószyński 2017: 15, figs 4B, 5C-D (mf); Prószyński 2018: 26, fig. 5H (mf); Peng 2020: 308, fig. 221a-h; Chen et al. 2021: 295, fig. 6A-C.
*Phintellapaminta* Barrion, Barrion-Dupo & Heong, in Barrion et al. 2013: 25, fig. 27A-C (f).
*Phintelloidesversicolor* (C. L. Koch, 1846) - Lin et al. 2023: 513 (synonymy with *Phintellapaminta*).

#### Materials

- **Type status:** Other material. **Occurrence:** catalogNumber: MMUE G7572.6412; recordedBy: F. & J. A. Murphy; individualCount: 1; sex: female; lifeStage: adult; otherCatalogNumbers: https://doi.org/10.3535/MDR-6FG-49E; occurrenceID: 12EAA5CD-517E-599F-98D5-C0A3F615F096; **Taxon:** scientificName: Phintelloidesminuta; **Location:** country: Hong Kong; locality: Mai Po mangrove; verbatimCoordinates: 22°29’N 114°02’E; decimalLatitude: 22.483333333333; decimalLongitude: 114.03333333333; **Event:** eventDate: 1988-02-27; fieldNumber: CM15605; fieldNotes: DSC 5976-5983; **Record Level:** institutionID: https://ror.org/027m9bs27; institutionCode: MMUE; basisOfRecord: PreservedSpecimen

#### Diagnosis

Tentatively, judging from drawings of Prószyński and Zabka, the rta seems less slender at the base in *munita* than in* versicolor*; furthermore, there could be a difference in shape of the retrolateral lobe of the tegulum, which arises retrolaterally alongside the down-turned branch of the U-bend of the sperm duct, versus from the distalmost tip of the U in *versicolor; *also the embolus is slightly stouter and bent directly at the base*. *The single female examined has a carapace ornamentation similar to that in *versicolor*; the abdomen wears ventrally numerous elongate iridescent setae. In live specimens, differences in carapace and abdomen decoration pattern probably do exist, including local variations. The epigyne is distinctive: copulatory ducts are stouter than in *versicolo*r (Fig. 18c, e) and diverging (Fig. 18d, f), the transverse folds running from the outer edge of the copulatory openings continue as an uninterrupted fold or bar all across the anterior edge of the epigyne (Fig. 18d, f). We have not been able to examine a male of this species.

#### Distribution

Japan, China, Hong Kong, Vietnam, North Korea, South Korea.

#### Notes

The World Spider Catalog (2024) erroneously indicates that the treatment of this species in Hu (2001) is based on the female; it is in fact based on the male.

## Supplementary Material

XML Treatment for Chrysilla

XML Treatment for Chrysilla lauta

XML Treatment for Chrysilla volupe

XML Treatment for Chrysilla deelemani

XML Treatment for Phintelloides

XML Treatment for Phintelloides flavumi

XML Treatment for Phintelloides jesudasi

XML Treatment for Phintelloides scandens

XML Treatment for Phintelloides versicolor

XML Treatment for Phintelloides minuta

## Figures and Tables

**Table:** 

Nanopublication	Creator	Date
https://w3id.org/kpxl/pensoft/bdj/np/RADCQw2GTQ6neJ7ahujuTTrUDfkFpQQIAt_tmvEwkBdwk	Jeremy Miller	03-09-2024 07:27:47
https://w3id.org/kpxl/pensoft/bdj/np/RAcxVlY7nnETh6yAfaFrSB8Z2p1rUnlzJVle83x7BPGtQ	Jeremy Miller	03-09-2024 07:31:24
https://w3id.org/kpxl/pensoft/bdj/np/RAKL3bScU3hWbi8D1J5ylR8pHvjDHJRhQ6pH3iopw7_nI	Jeremy Miller	03-09-2024 07:33:21
https://w3id.org/kpxl/pensoft/bdj/np/RAdAhuDmXh3rinBffEpE-t1-ZwfMZ5UUTZwpO_936qy7A	Jeremy Miller	03-09-2024 09:15:47
https://w3id.org/kpxl/pensoft/bdj/np/RAaVPpGkFqNtrG_CRJzLW4-E1A5R3i6C-Vs_w986BCU0Y	Jeremy Miller	03-09-2024 07:39:18
https://w3id.org/kpxl/pensoft/bdj/np/RAMDblQmTnloCt9X4DzpL1tIm0k3Esvf0h0JERmn2-BeA	Jeremy Miller	03-09-2024 07:41:16
https://w3id.org/kpxl/pensoft/bdj/np/RA09hShbZtF5u9wYyokmZT2bXHlOLMxTAJjjux2XmSWWw	Jeremy Miller	03-09-2024 07:44:18
https://w3id.org/kpxl/pensoft/bdj/np/RAp7dPJ6ZCU8WY0XTv31KVeSR99gMDGaYjh2exyoZIS-8	Jeremy Miller	03-09-2024 07:46:58
https://w3id.org/kpxl/pensoft/bdj/np/RAWrJCNXa2PXpZ9s_-0YolXJXFI6NA6QdheaVReFnU7oc	Jeremy Miller	03-09-2024 07:48:54
https://w3id.org/kpxl/pensoft/bdj/np/RAY2l9Fr74pss6Ryu4cIWvEqVIuFBcaSLVNXZ4rq_fCZQ	Jeremy Miller	03-09-2024 07:51:00
https://w3id.org/kpxl/pensoft/bdj/np/RAMz_plRds4hHYXz0iPSyOMHySr8uxaT08O73ewb8H5PQ	Jeremy Miller	03-09-2024 07:53:04
https://w3id.org/kpxl/pensoft/bdj/np/RARyotcpBJJ-sNBDAflyEsObIcFh40WoNtB4nP8h0GUm0	Jeremy Miller	03-09-2024 07:55:06
https://w3id.org/kpxl/pensoft/bdj/np/RA0KFO4XvMOoUc-DJDJnbqqNHw8eYSSxyzQwceaqHcOyw	Jeremy Miller	03-09-2024 07:57:08
https://w3id.org/kpxl/pensoft/bdj/np/RAaVOMwiD9OCg1pu7dez9mIR1IJvXAb_ARvFsQxojwMwI	Jeremy Miller	03-09-2024 07:59:42
https://w3id.org/kpxl/pensoft/bdj/np/RAJmaVNQ7V9vZMaw3tNqoq7y4vZIhFreEhcDp7rtZxbeo	Jeremy Miller	03-09-2024 08:01:28
https://w3id.org/kpxl/pensoft/bdj/np/RAWQdMpuq0sJqc88mqs4oAGGShEZfX-i7AgoL0luZJ7VM	Jeremy Miller	03-09-2024 08:03:22
https://w3id.org/kpxl/pensoft/bdj/np/RA_BNcD8wc_zk_Qv4CDF1s408C300qFvyUGnYZM2n-uSk	Jeremy Miller	03-09-2024 08:05:10
https://w3id.org/kpxl/pensoft/bdj/np/RAnuE8alNpM59WiNuDYb-3StjyLkKBk8YD276CJGk1J3Q	Jeremy Miller	03-09-2024 08:07:08
https://w3id.org/kpxl/pensoft/bdj/np/RA8jERliEmc4C9roxr81xU0Rw4Fjsb6PEc7UKihPvI-K0	Jeremy Miller	03-09-2024 08:17:42
https://w3id.org/kpxl/pensoft/bdj/np/RA6Ter8zNsOdUYoDz4uXA5mcnKEPFivYoC3rzSlesmbOg	Jeremy Miller	03-09-2024 08:19:38
https://w3id.org/kpxl/pensoft/bdj/np/RAJfbsbKpr3l6XqYA35q8gUxd6gcu-gHO5_7UJ5Tox-Kk	Jeremy Miller	03-09-2024 08:21:09
https://w3id.org/kpxl/pensoft/bdj/np/RA2CPm-r90Wxwt6kIHA2jB1bahn1PHJOhT5axYt2jr5i8	Jeremy Miller	03-09-2024 08:22:53
https://w3id.org/kpxl/pensoft/bdj/np/RAqG0CnQz0uleZoNXx_x7iqzpkrlimN2AqVAFBZ2EqjYo	Jeremy Miller	03-09-2024 08:24:26
https://w3id.org/kpxl/pensoft/bdj/np/RAchxP0Z5YmbcTm9Sjf9B6s4ZoQ5rfv_XBNcKG61x1-E4	Jeremy Miller	03-09-2024 08:28:02
https://w3id.org/kpxl/pensoft/bdj/np/RAiyoZxuqroBIFp-Yb7d287U9DFE91U8xjBj_-20-Vcaw	Jeremy Miller	03-09-2024 08:29:54
https://w3id.org/kpxl/pensoft/bdj/np/RAOmgAo2GsgM1dMln5K6_CuJU4Getx7FUspyrR-FZsCKk	Jeremy Miller	03-09-2024 08:31:53
https://w3id.org/kpxl/pensoft/bdj/np/RAoNbIVzr1BdUwDCKmKCmC4ZBcE0tYQvQWbCce6eJ-nY0	Jeremy Miller	03-09-2024 08:34:09
https://w3id.org/kpxl/pensoft/bdj/np/RAbUBnrgEx7im12V39klqLD1BaUJ3oMIwGhNvssp5rYYQ	Jeremy Miller	03-09-2024 08:35:51
https://w3id.org/kpxl/pensoft/bdj/np/RA0HML1IN_kVMGeIZMAF3OA23ZoLX5FdY8kvWkamgTQAM	Jeremy Miller	03-09-2024 08:37:25
https://w3id.org/kpxl/pensoft/bdj/np/RAelQt4dXL5X-w132Nu2ayp-rlFLWw8UkOC5F-gVCO7hc	Jeremy Miller	03-09-2024 08:39:22
https://w3id.org/kpxl/pensoft/bdj/np/RAM7QwOnH20W6bgFcA1ZI4eZ2W0bjFpWxhml8IwlZ3xZA	Jeremy Miller	03-09-2024 08:41:07
https://w3id.org/kpxl/pensoft/bdj/np/RAvpRnVy7PL1j_irhV-_RQQ1VK_ZApBjDuHA1oe-8AYW4	Jeremy Miller	03-09-2024 08:42:33
https://w3id.org/kpxl/pensoft/bdj/np/RAvqWxnJn3kEcNOYWisDj1OzVKkxFivDawofvSekhMhdE	Jeremy Miller	03-09-2024 08:44:16
https://w3id.org/kpxl/pensoft/bdj/np/RAXzzQ6BbA1QlAUmYKRuAWXJV_3K7DPcaM-8Jf6bQuvlM	Jeremy Miller	03-09-2024 08:45:49
https://w3id.org/kpxl/pensoft/bdj/np/RAZz1UsfZxYZI4ghpum4wNPg6idsurBN7uGDvXogUSPTo	Jeremy Miller	03-09-2024 08:47:36
https://w3id.org/kpxl/pensoft/bdj/np/RAczrJfTcHYfpUY6Jx9D2buN-9iEa4ji8I_m0OOIY0Cuw	Jeremy Miller	03-09-2024 08:49:16
https://w3id.org/kpxl/pensoft/bdj/np/RAczrJfTcHYfpUY6Jx9D2buN-9iEa4ji8I_m0OOIY0Cuw	Jeremy Miller	03-09-2024 08:49:16
https://w3id.org/kpxl/pensoft/bdj/np/RAQ8td8QmYMVXtmXedT2oXIZMIQF8bSLB4W0jQd1UXOnQ	Jeremy Miller	03-09-2024 08:52:59
https://w3id.org/kpxl/pensoft/bdj/np/RAy-K7ltX7zJfvMZa_VsRd8Yprbs8N1XyfRwAT7WUIGYQ	Jeremy Miller	03-09-2024 08:54:22
https://w3id.org/kpxl/pensoft/bdj/np/RAxMzS1UXicPn3bBGAs1p3pBYoLpaXgcZJTw2wFwIaXiQ	Jeremy Miller	03-09-2024 08:55:51
https://w3id.org/kpxl/pensoft/bdj/np/RAcdUMO5WvsFq2z7NabKJ8Ec6okJcvYoa_NH4eTGjDRgQ	Jeremy Miller	03-09-2024 08:57:24
https://w3id.org/kpxl/pensoft/bdj/np/RAwjTOock3z04LLjqVDI-B8_ljOVdnZ2sW0Uy3YsLbxjM	Jeremy Miller	03-09-2024 08:59:12
https://w3id.org/kpxl/pensoft/bdj/np/RA27BIVpKwGMM8Bq2vq2s_TT7webZoWvvFo4ura5OVBRw	Jeremy Miller	03-09-2024 09:01:22
https://w3id.org/kpxl/pensoft/bdj/np/RAGmdqKLfh6v--ijVzg0x_S_UUMj5mD3kZOxtPs_24oZg	Jeremy Miller	03-09-2024 09:02:50
https://w3id.org/kpxl/pensoft/bdj/np/RA0VrGBWxK-UQSfvmMwo1obl9K50OZJna4Rh5Ll1NGwEg	Jeremy Miller	03-09-2024 09:04:14
https://w3id.org/kpxl/pensoft/bdj/np/RAo1zrOCBu33MPk9aD8yXOg3gJHKj23wyy34Tx2Yh_7xI	Jeremy Miller	03-09-2024 09:06:45
https://w3id.org/kpxl/pensoft/bdj/np/RAnFSyaKl9wAF4YLbXfSCXOHaL6HPUeq4iAnTEyhLo-G0	Jeremy Miller	03-09-2024 09:09:34
https://w3id.org/kpxl/pensoft/bdj/np/RAMzQ8gVdAQZFlqEb2bvScczISxwaGV4AgLi1B8_fwoRM	Jeremy Miller	03-09-2024 09:11:10

**Figure 1a.**
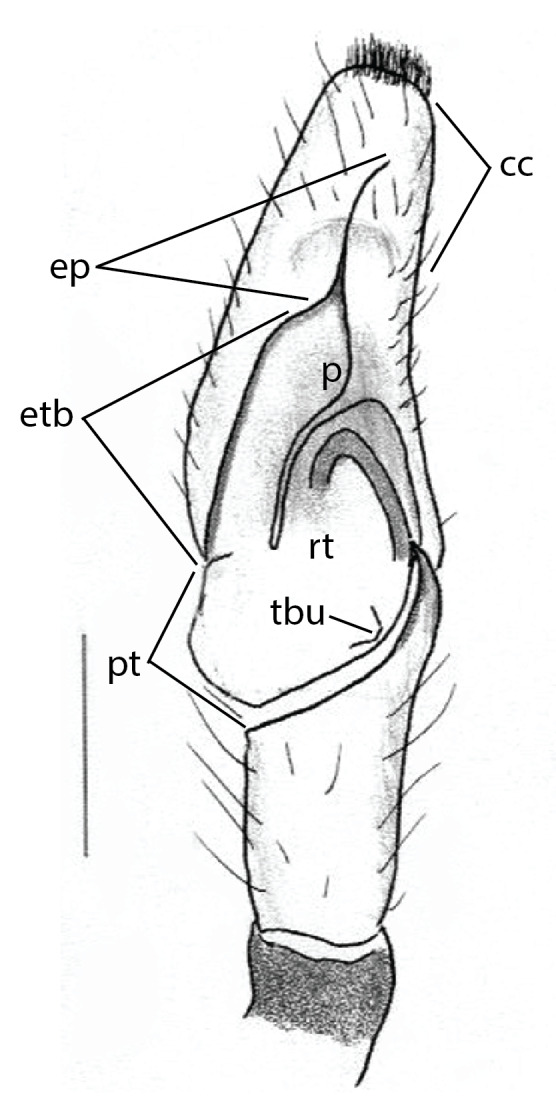
*Chrysillalauta* Thorell, 1887, left male pedipalp, ventral view **cc** cymbium cap **ep** embolus proper **etb** embolar tegular branch **p** distal projection of embolar tegular branch beyond retrolateral lobe of tegulum excluding embolus proper **pt **proximal lobe of tegulum **rt **retrolateral lobe of tegulum **tbu** tegular bump. Scale bar: 0.3 mm

**Figure 1b.**
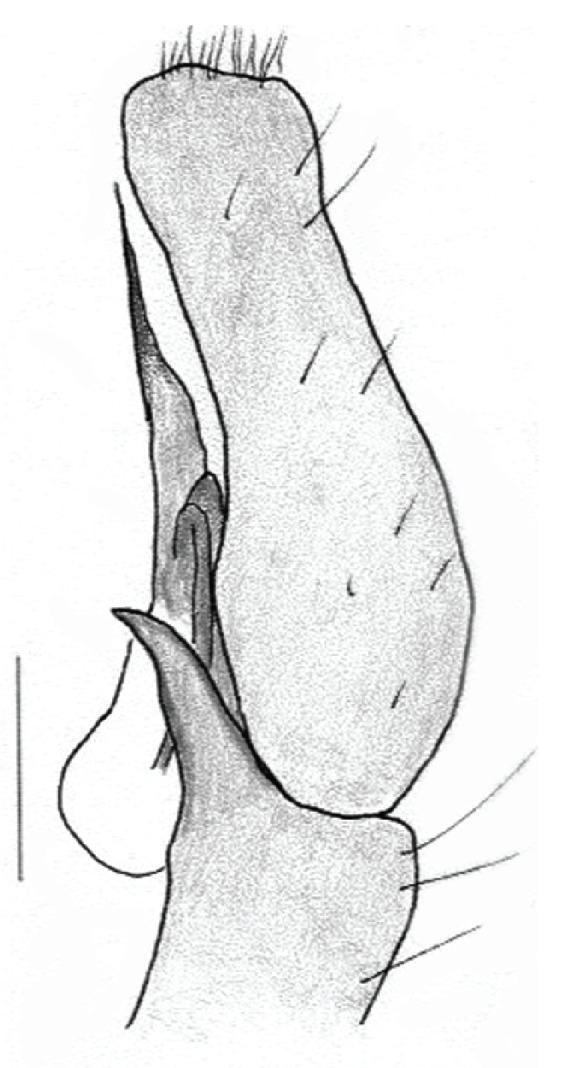
*Chrysillalauta* Thorell, 1887, left male pedipalp, retrolateral view. Scale bar: 0.3 mm

**Figure 1c.**
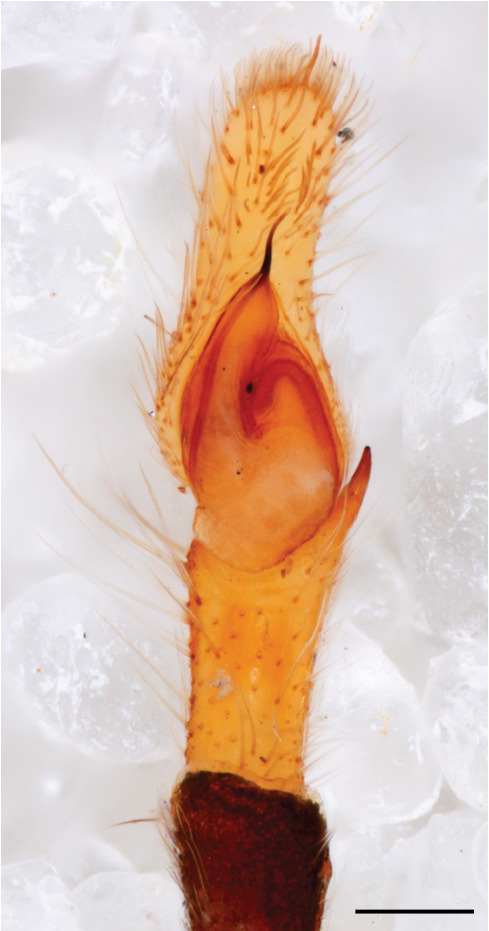
*Chrysillalauta* Thorell, 1887, ventral view, CM 15726, scale bar 0.2 mm

**Figure 1d.**
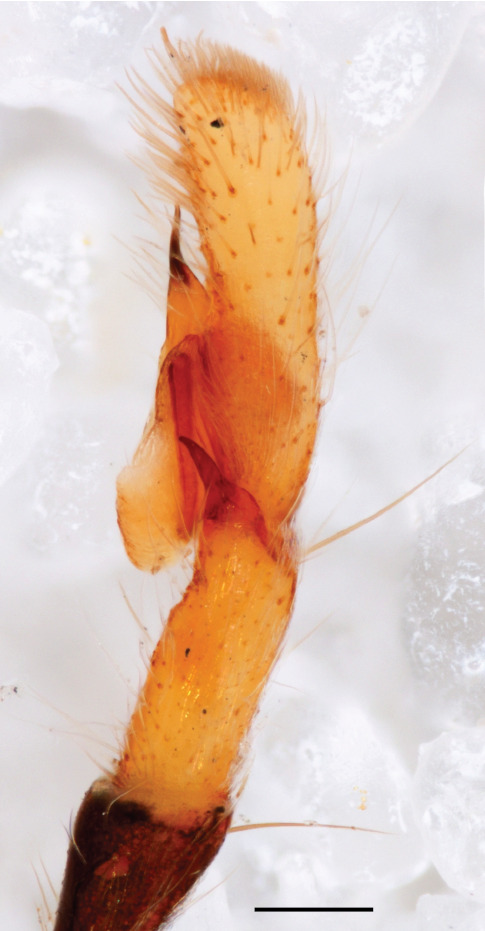
*Chrysillalauta* Thorell, 1887, retrolateral view, CM 15726, scale bar 0.2 mm

**Figure 2a.**
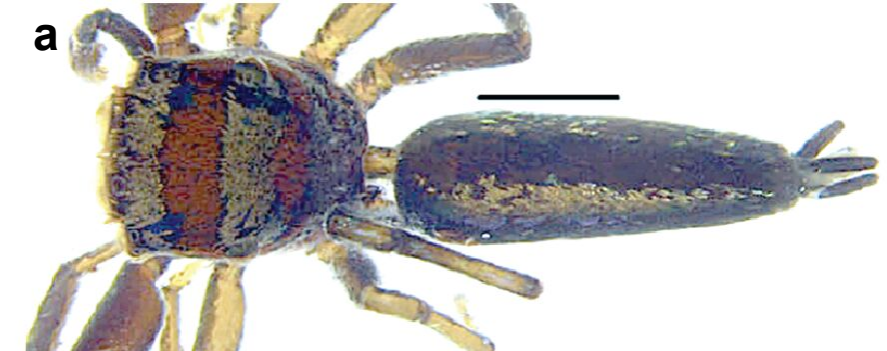
*Chrysillalauta* Thorell, 1887, Kanesharatnam and Benjamin, 2019, fig. 19A, male habitus, dorsal view. Scale bar 1 mm

**Figure 2b.**
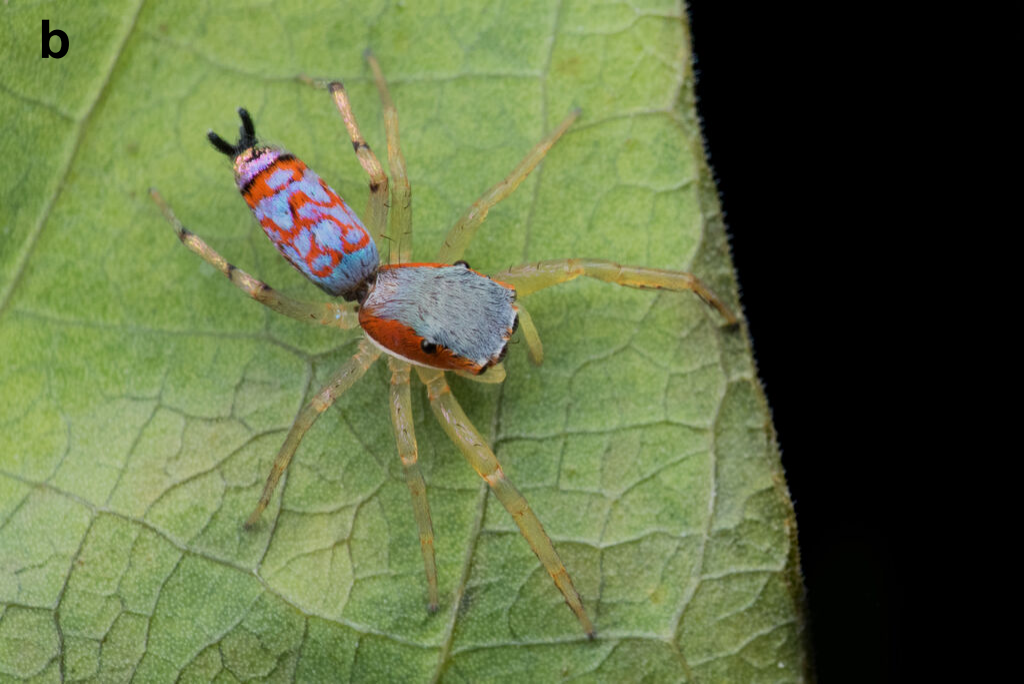
*Chrysillalauta* Thorell, 1887, Koh et al., 2022, p. 347, live female, reproduced with permission, photo credit Paul Y.C. Ng

**Figure 2c.**
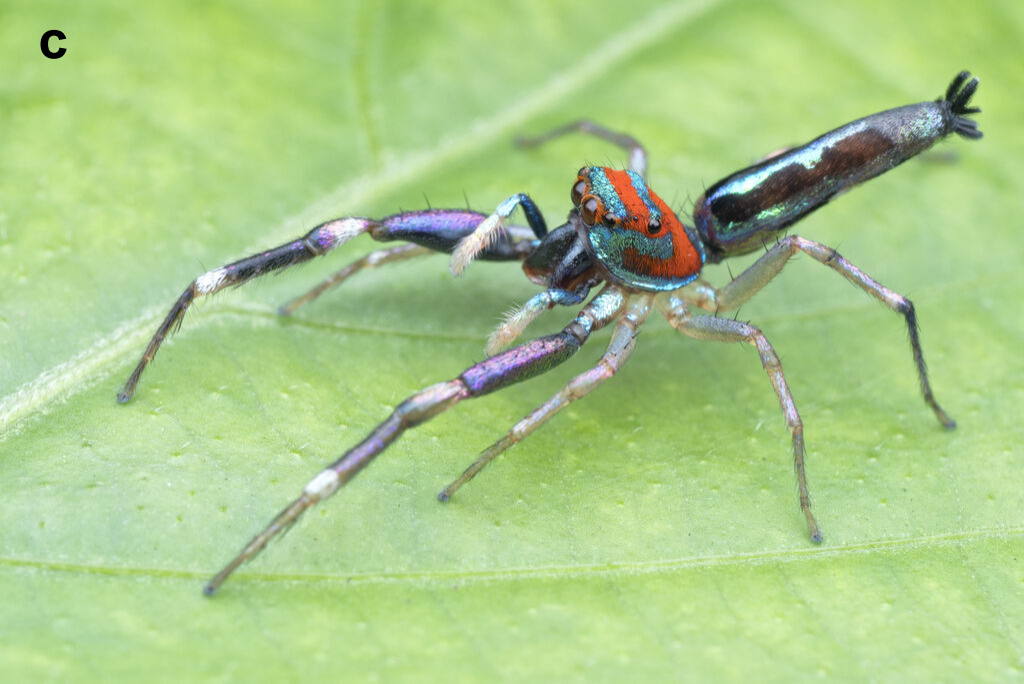
*Chrysillalauta* Thorell, 1887, Koh et al., 2022, p. 347, live male, reproduced with permission, photo credit Melvyn Yeo

**Figure 2d.**
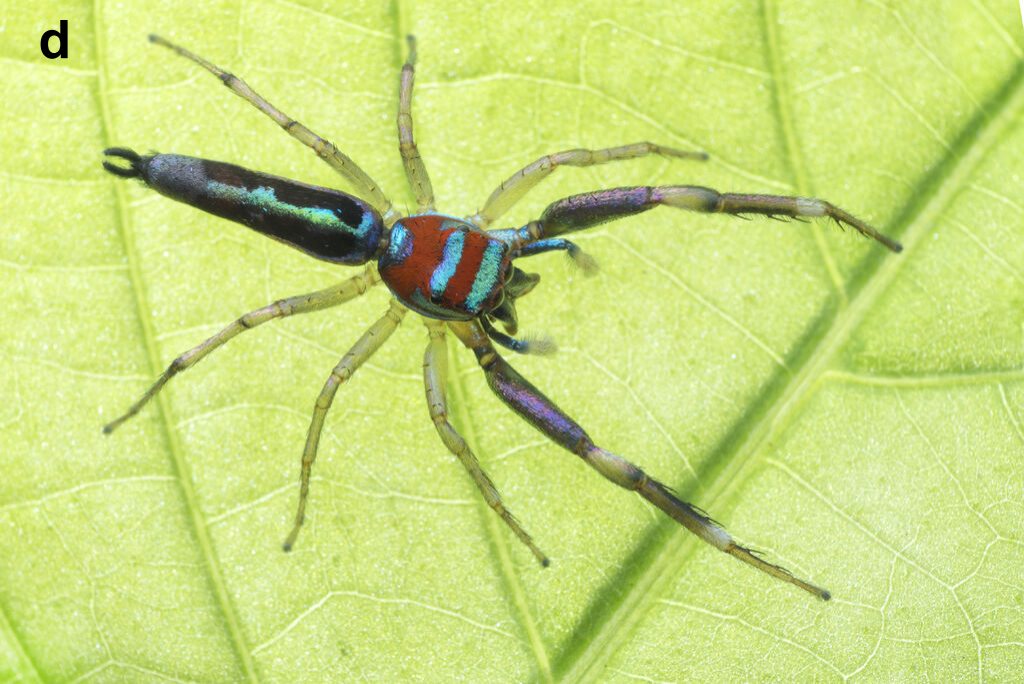
*Chrysillalauta* Thorell, 1887, Koh et al., 2022, p. 347, live male, reproduced with permission, photo credit Melvyn Yeo

**Figure 3a.**
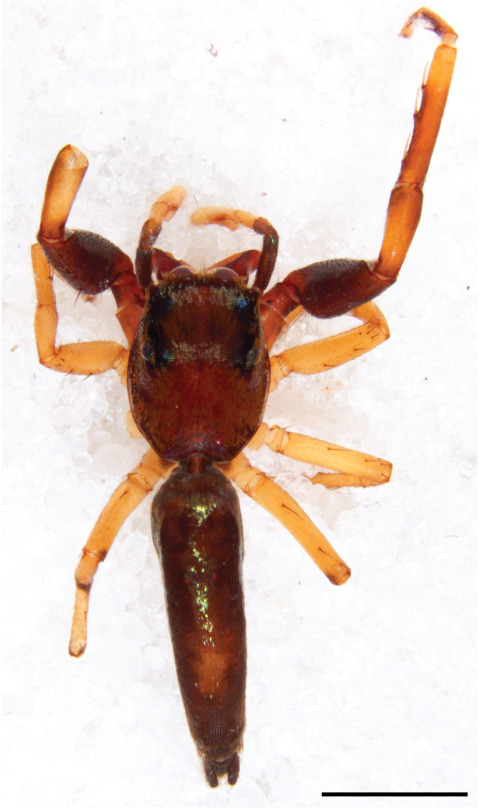
*Chrysillalauta* Thorell, 1887, male habitus, dorsal view, CM 15726, scale bar 2 mm

**Figure 3b.**
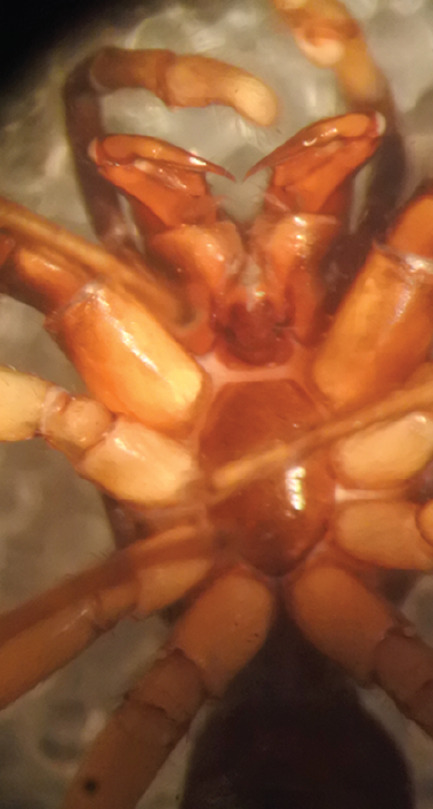
*Chrysillalauta* Thorell, 1887, male prosoma, ventral view, CM 15726

**Figure 3c.**
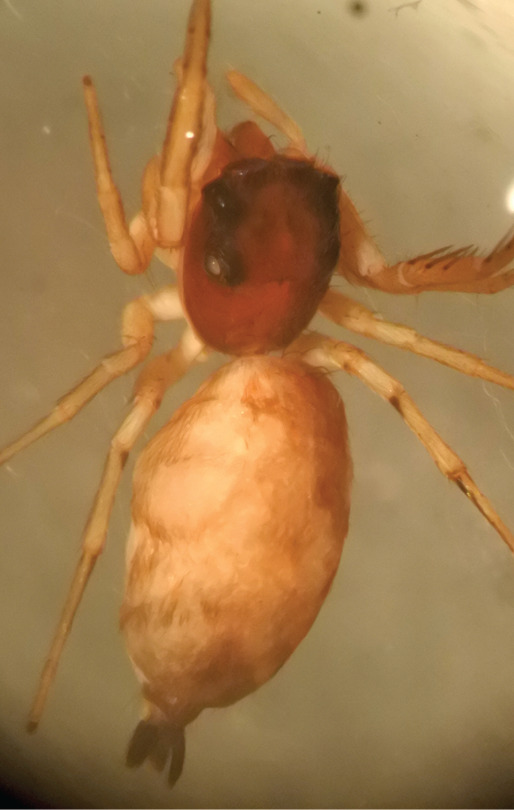
*Chrysillalauta* Thorell, 1887, female habitus, dorsal view, CM19182

**Figure 3d.**
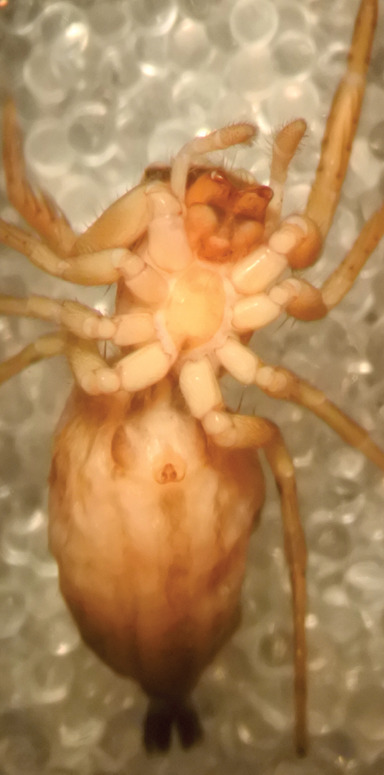
*Chrysillalauta* Thorell, 1887, female habitus, ventral view, CM19182

**Figure 4a.**
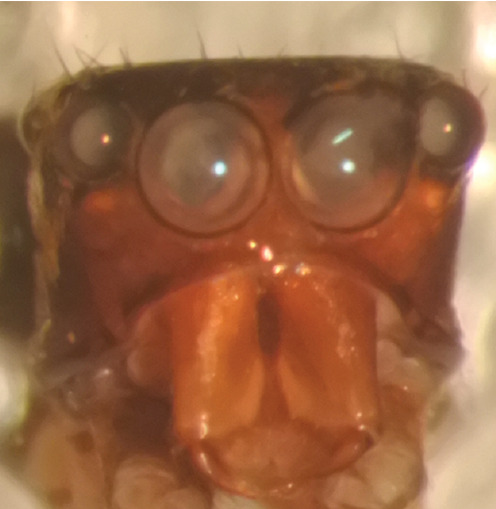
*Chrysillalauta* Thorell, 1887, male prosoma, anterior view, CM 15726

**Figure 4b.**
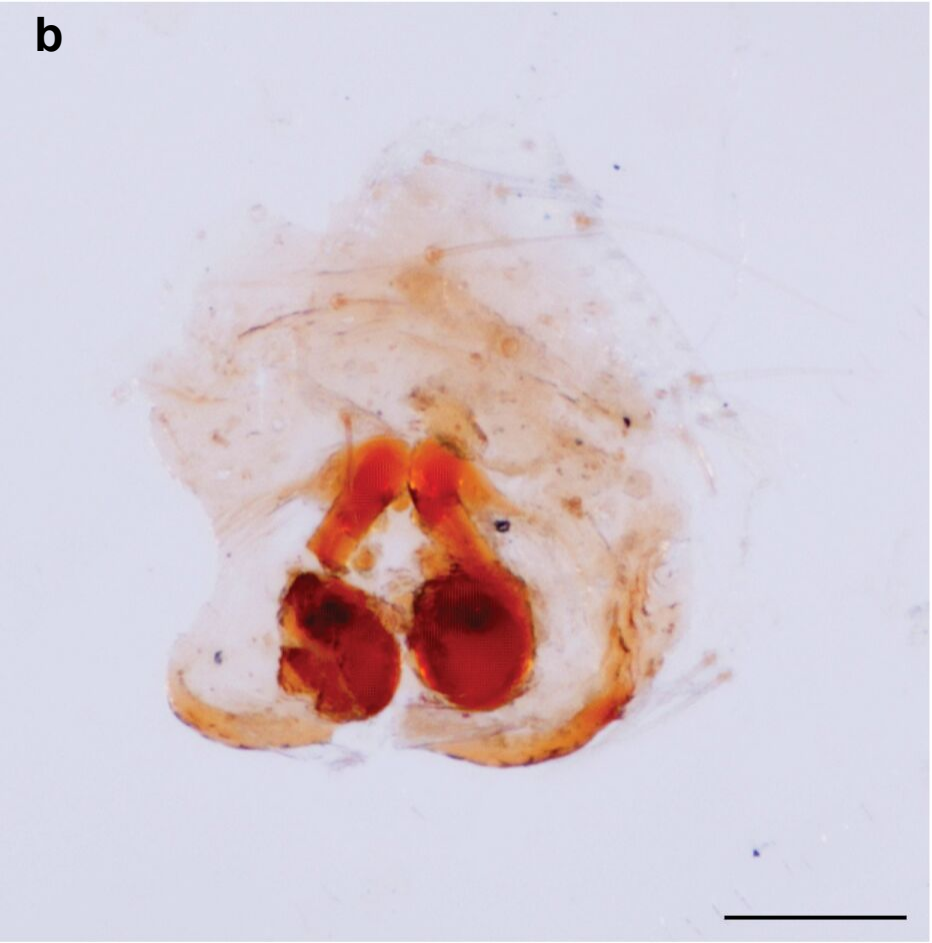
*Chrysillalauta* Thorell, 1887, female vulva, dorsal view, CM19182, scale bar 0.1 mm

**Figure 4c.**
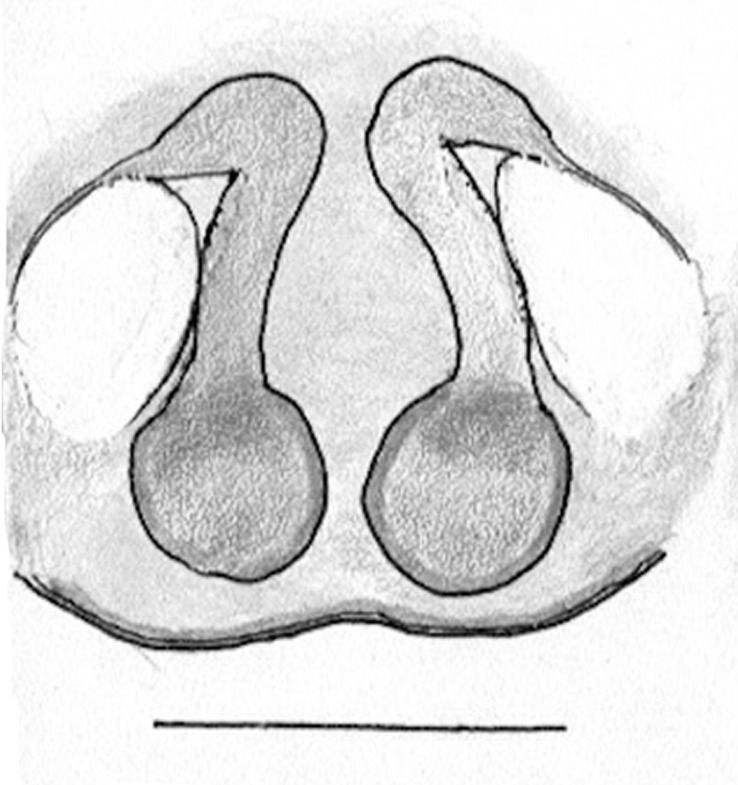
*Chrysillalauta* Thorell, 1887, female epigynum, ventral view, illustration, scale bar 0.2 mm

**Figure 4d.**
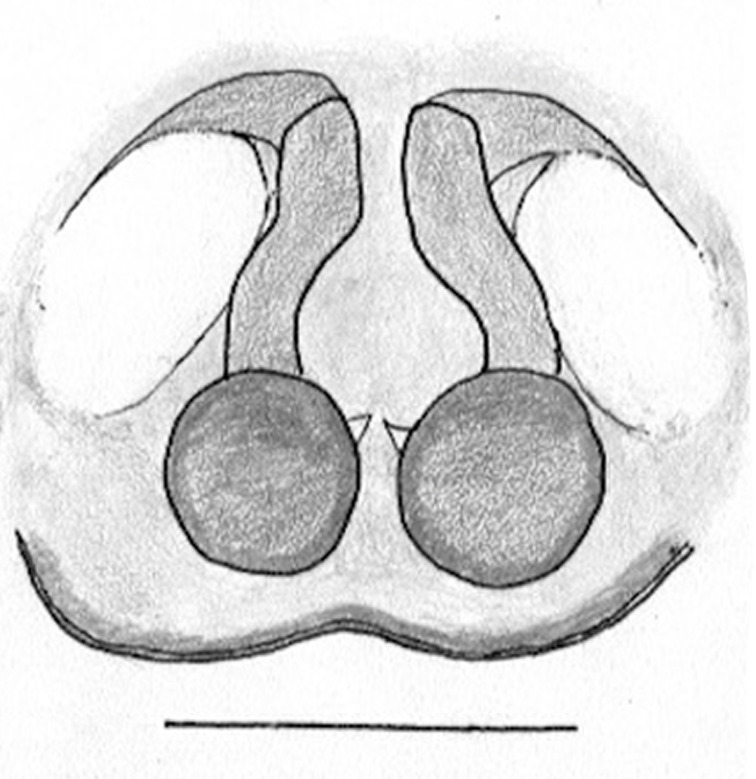
*Chrysillalauta* Thorell, 1887, female epigynum, dorsal view, illustration, scale bar 0.2 mm

**Figure 7a.**
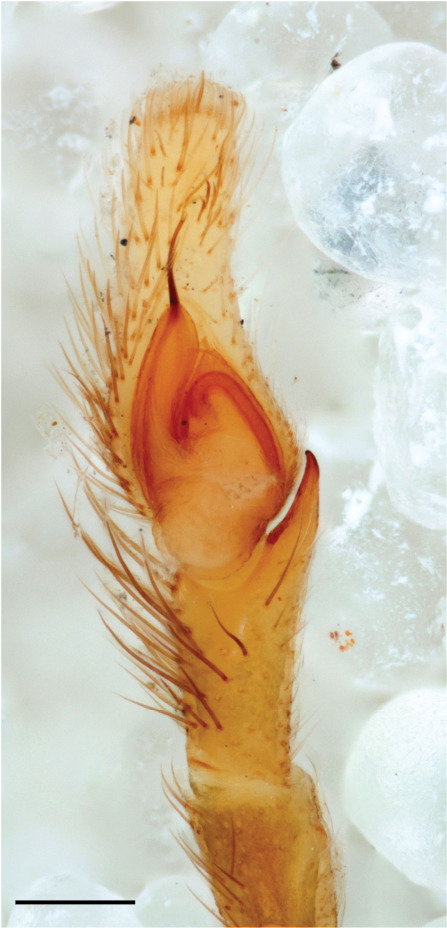
*Chrysillavolupe* (Karsch, 1879), reversed right male pedipalp, ventral view, CM 15916

**Figure 7b.**
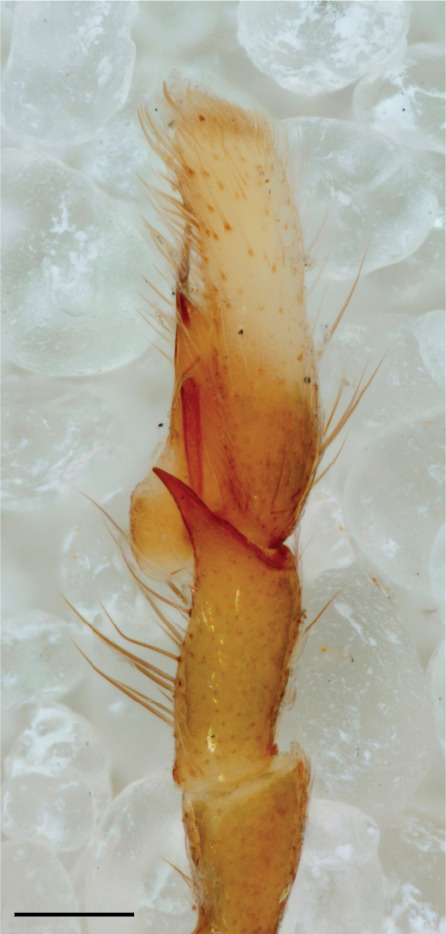
*Chrysillavolupe* (Karsch, 1879), reversed right male pedipalp, retrolateral view, CM 15916

**Figure 8a.**
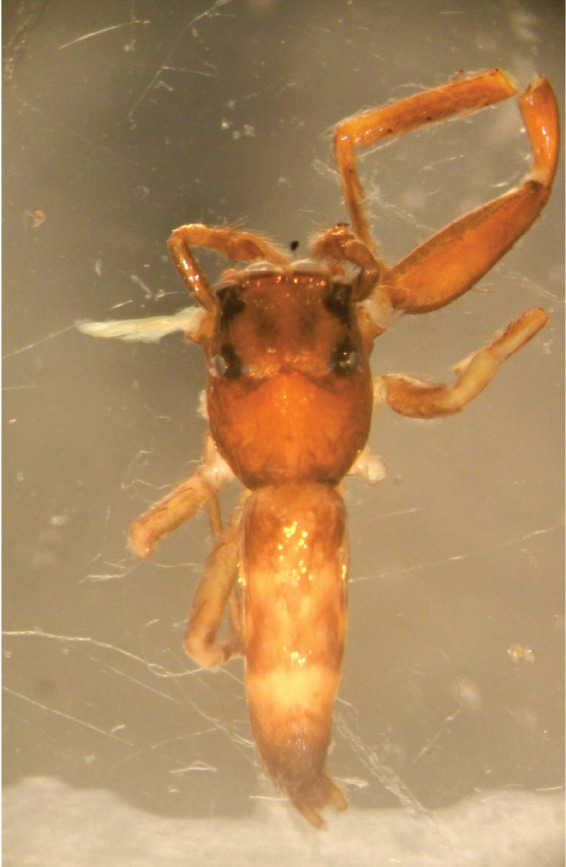
*Chrysillavolupe* (Karsch, 1879), male habitus, dorsal view, CM 15916

**Figure 8b.**
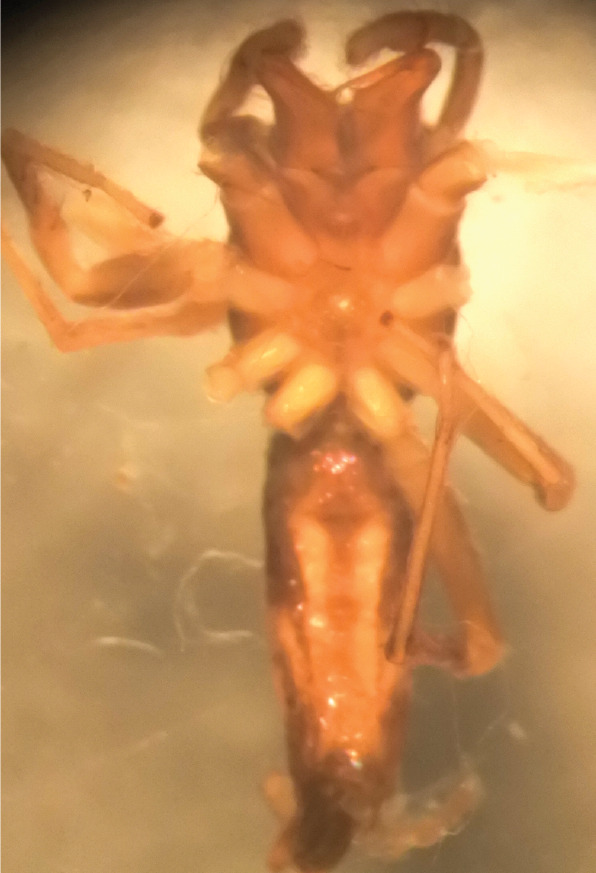
*Chrysillavolupe* (Karsch, 1879), male habitus, ventral view, CM 15916

**Figure 8c.**
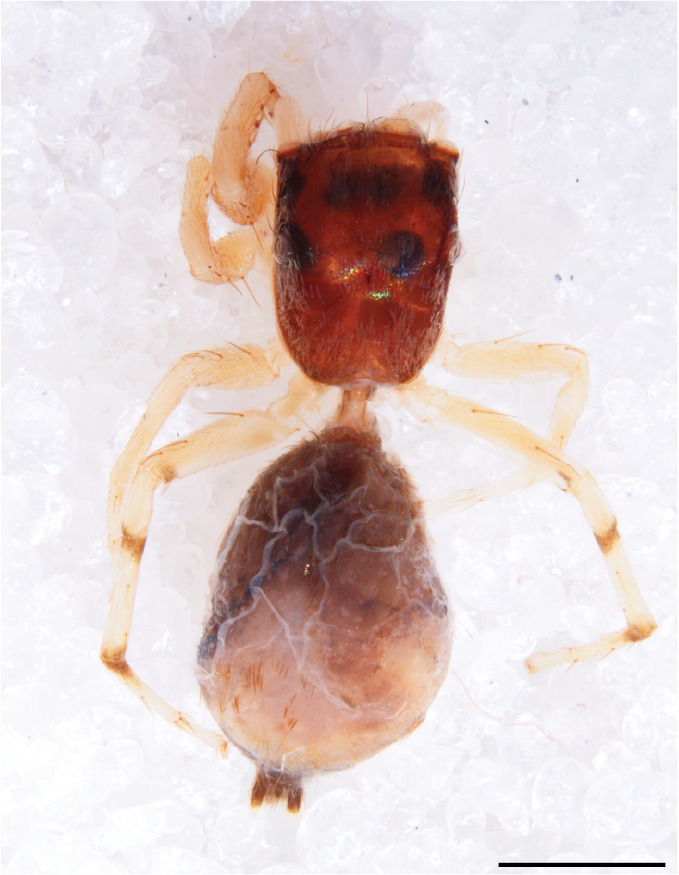
*Chrysillavolupe* (Karsch, 1879), female habitus, dorsal view, RMNH.ARA.18259, scale bar 1 mm

**Figure 8d.**
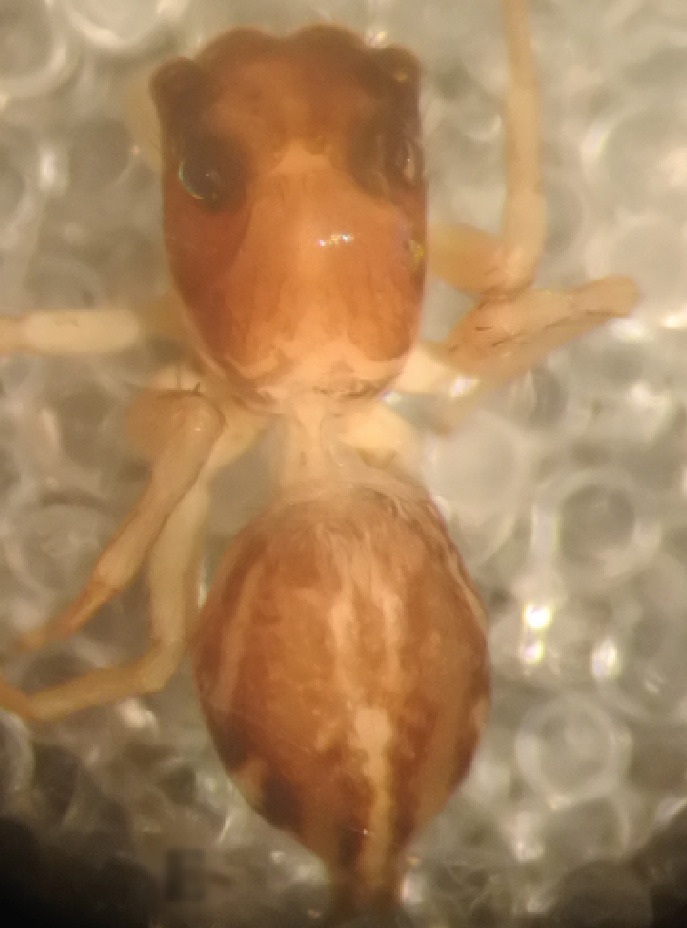
*Chrysilladeelemani* Prószyński & Deeleman-Reinhold, 2010, female habitus, dorsal view, RMNH.ARA.18265

**Figure 9a.**
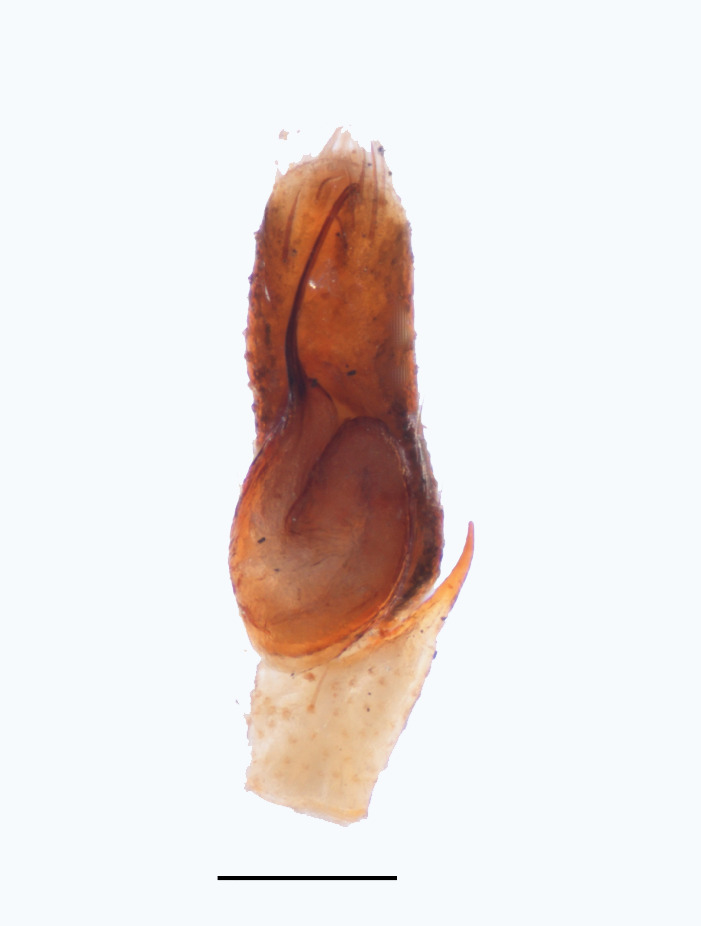
*Chrysilladeelemani* Prószyński & Deeleman-Reinhold, 2010, male pedipalp, ventral view, RMNH.ARA.18264, scale bar 0.2 mm

**Figure 9b.**
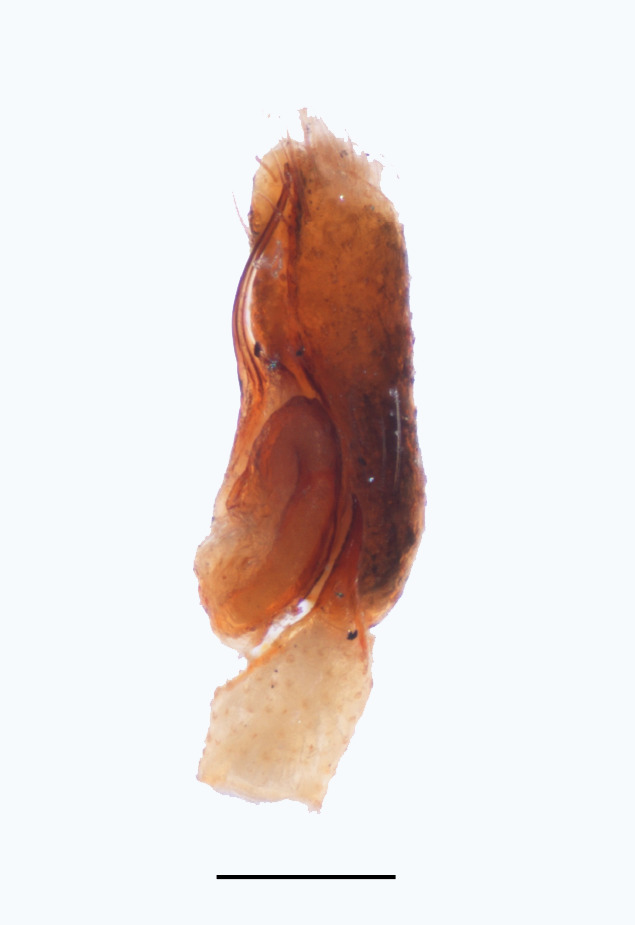
*Chrysilladeelemani* Prószyński & Deeleman-Reinhold, 2010, male pedipalp, retrolateral view, RMNH.ARA.18264, scale bar 0.2 mm

**Figure 9c.**
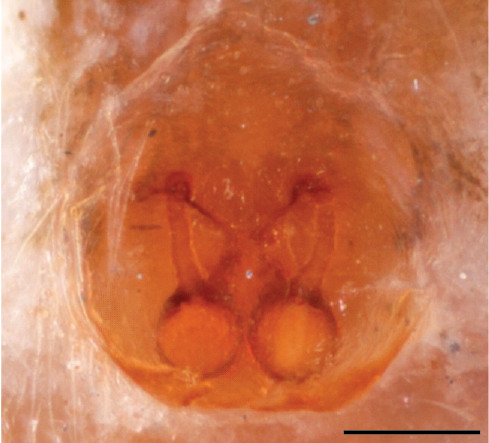
*Chrysilladeelemani* Prószyński & Deeleman-Reinhold, 2010, female epigynum, ventral view, RMNH.ARA.18265, scale bar 0.1 mm

**Figure 9d.**
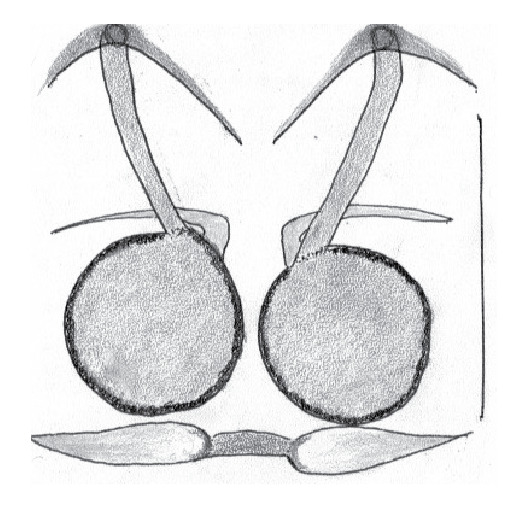
*Chrysilladeelemani* Prószyński & Deeleman-Reinhold, 2010, female vulva, dorsal view, illustration, scale bar 0.2 mm

**Figure 10a.**
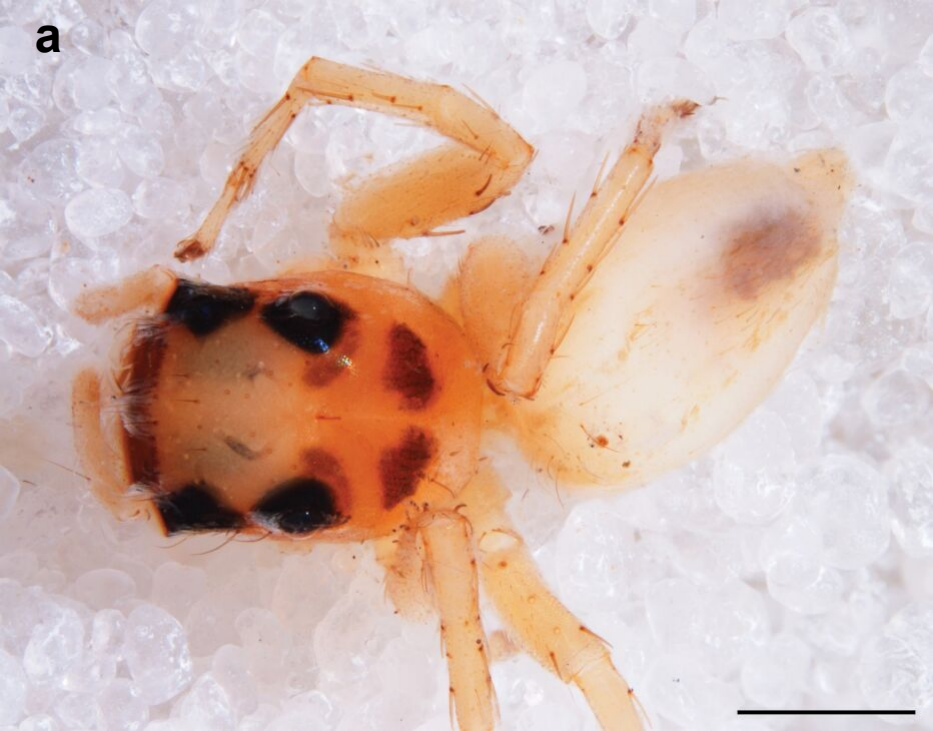
*Phintelloidesjesudasi* (Caleb & Mathai, 2014), female habitus, dorsal view, RMNH.ARA.18258, scale bar 1 mm

**Figure 10b.**
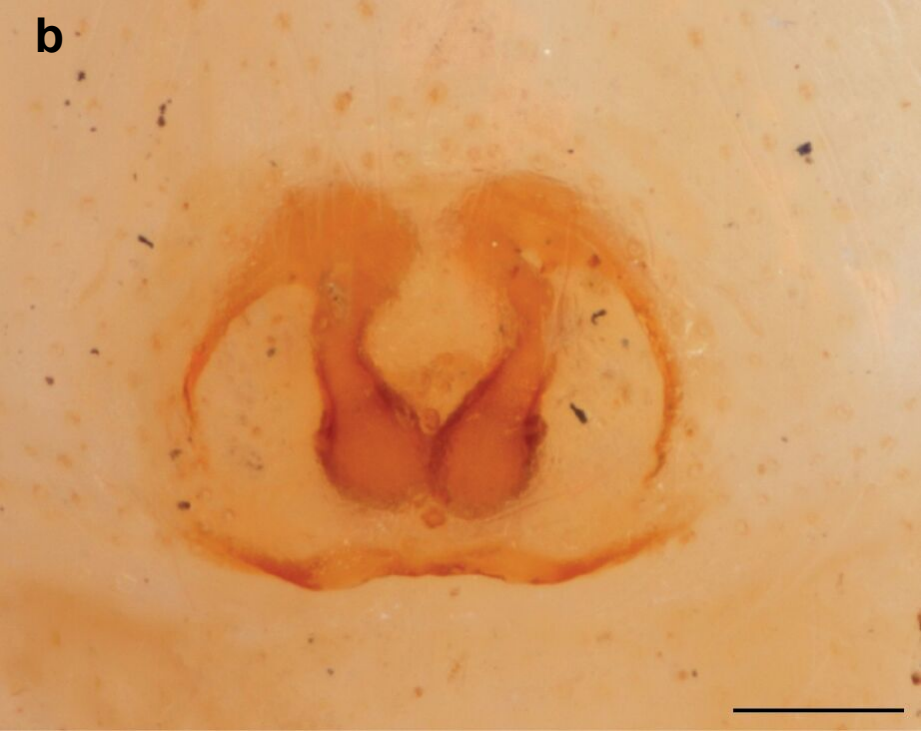
*Phintelloidesjesudasi* (Caleb & Mathai, 2014), female epigynum, ventral view, RMNH.ARA.18258, scale bar 0.1 mm

**Figure 10c.**
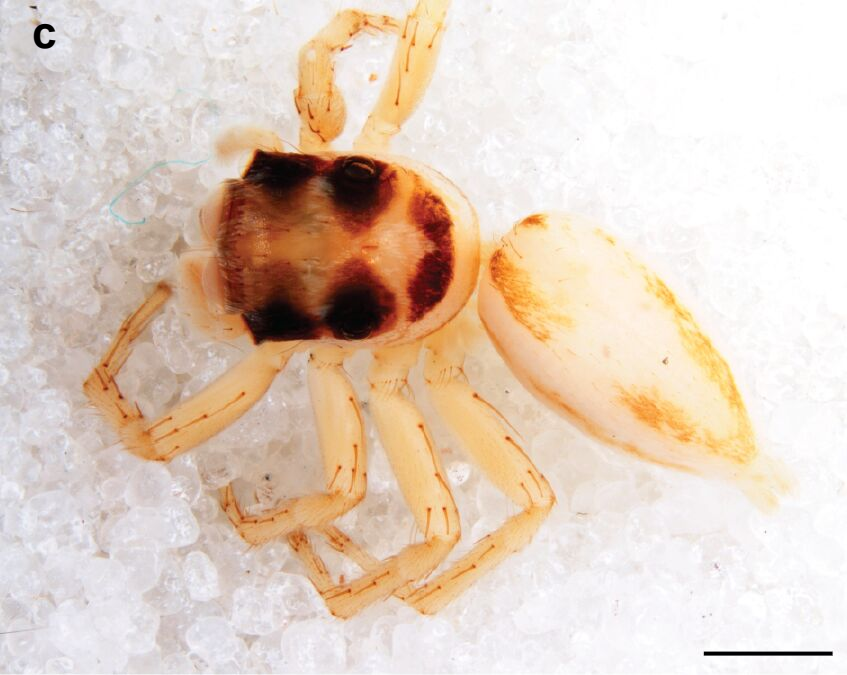
*Phintelloidesflavumi* Kanesharatnam & Benjamin, 2019, female habitus, dorsal view, RMNH.ARA.18250, scale bar 1 mm

**Figure 10d.**
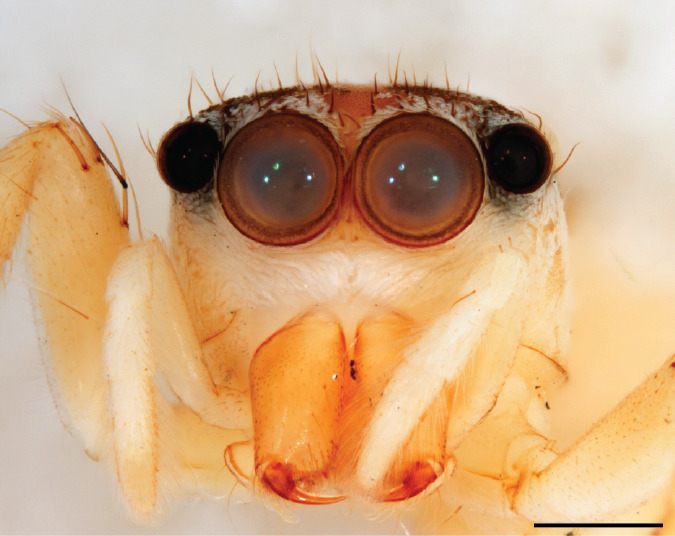
*Phintelloidesflavumi* Kanesharatnam & Benjamin, 2019, female prosoma, anterior view, RMNH.ARA.18250, scale bar 0.5 mm

**Figure 11a.**
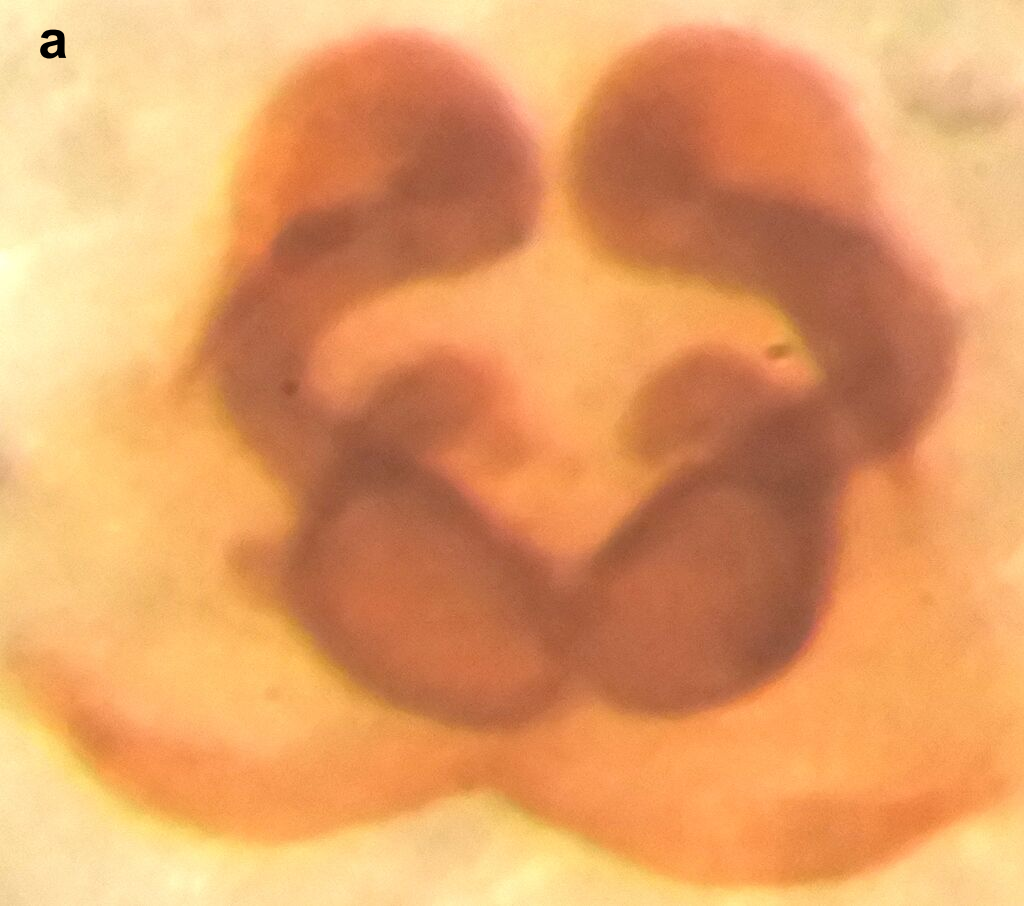
*Phintelloidesflavumi* Kanesharatnam & Benjamin, 2019, female vulva, dorsal view, RMNH.ARA.18250

**Figure 11b.**
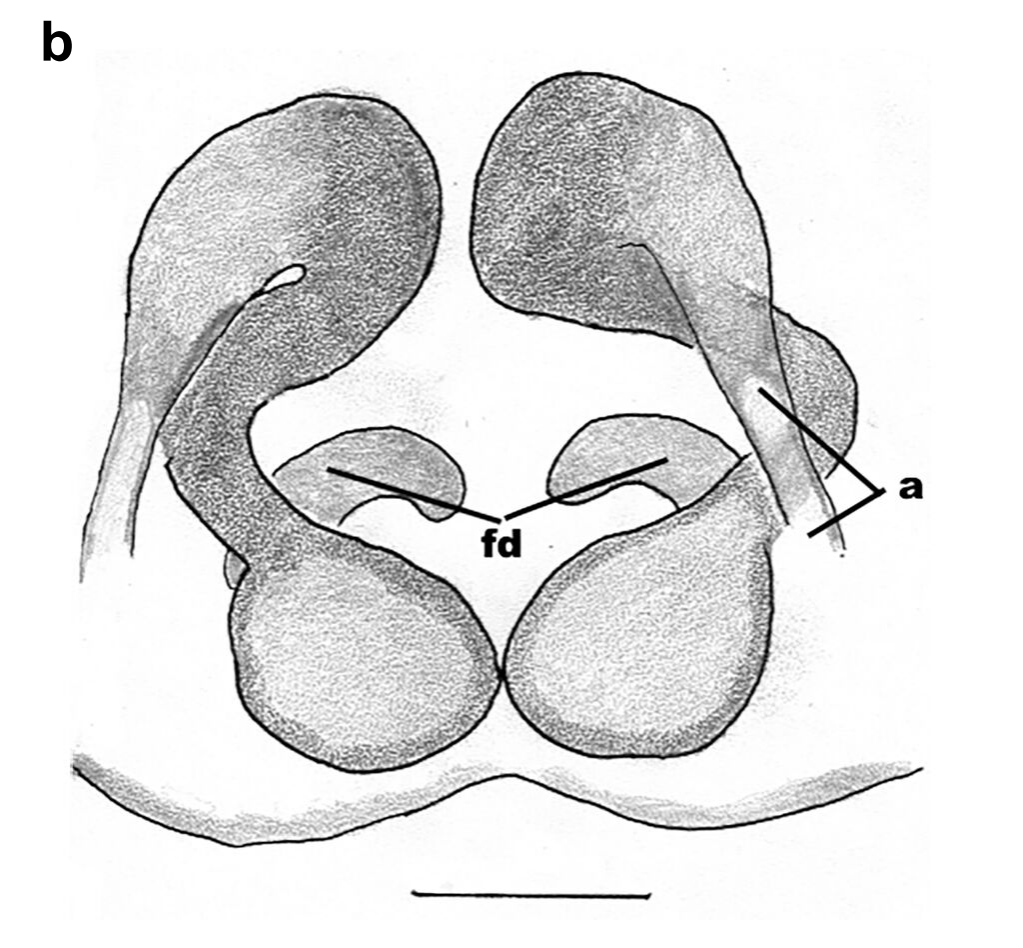
*Phintelloidesflavumi* Kanesharatnam & Benjamin, 2019, female vulva, dorsal view, illustration **a** atrium **fd** ferilization ducts. Scale bar 0.1 mm

**Figure 11c.**
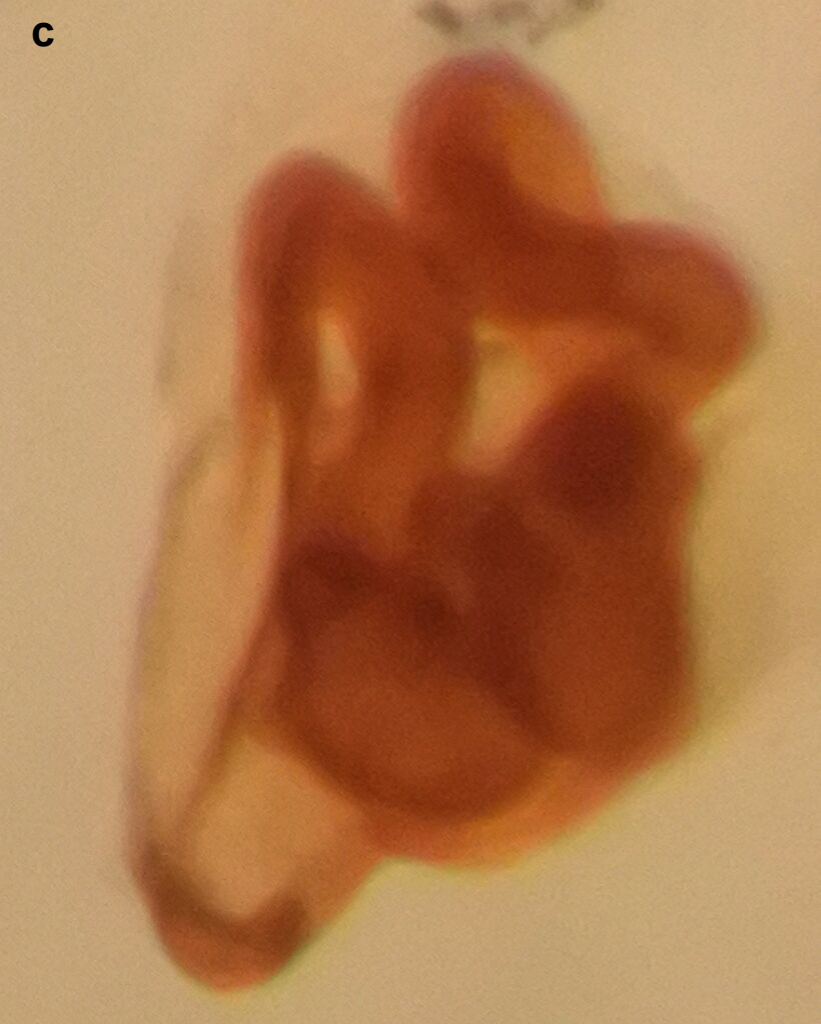
*Phintelloidesflavumi* Kanesharatnam & Benjamin, 2019, female vulva, oblique view, RMNH.ARA.18250

**Figure 11d.**
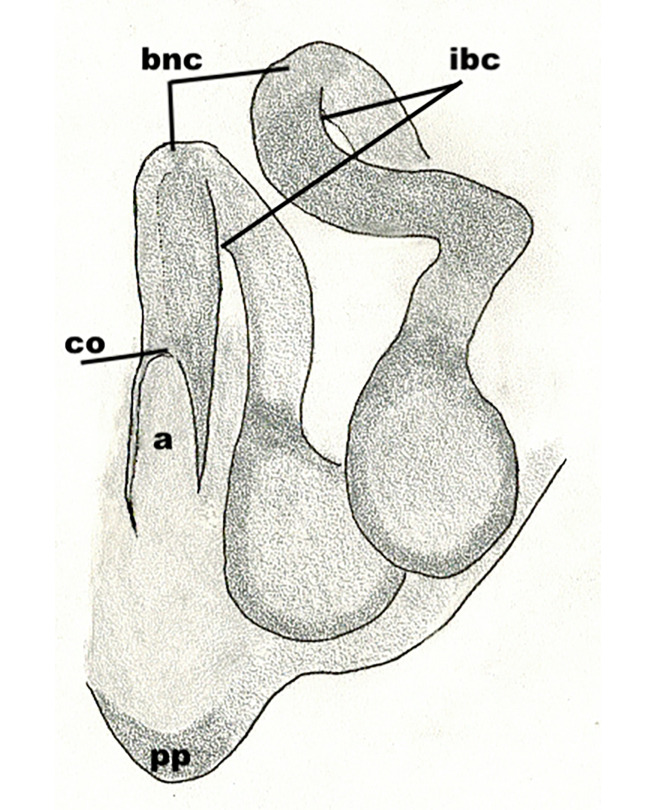
*Phintelloidesflavumi* Kanesharatnam & Benjamin, 2019, female vulva, oblique view, illustration **a** atrium **bnc** bird’s neck curve **co** copulatory opening **ibc** inner bend of bird’s-neck shaped curve **pp** posterior pockets

**Figure 12a.**
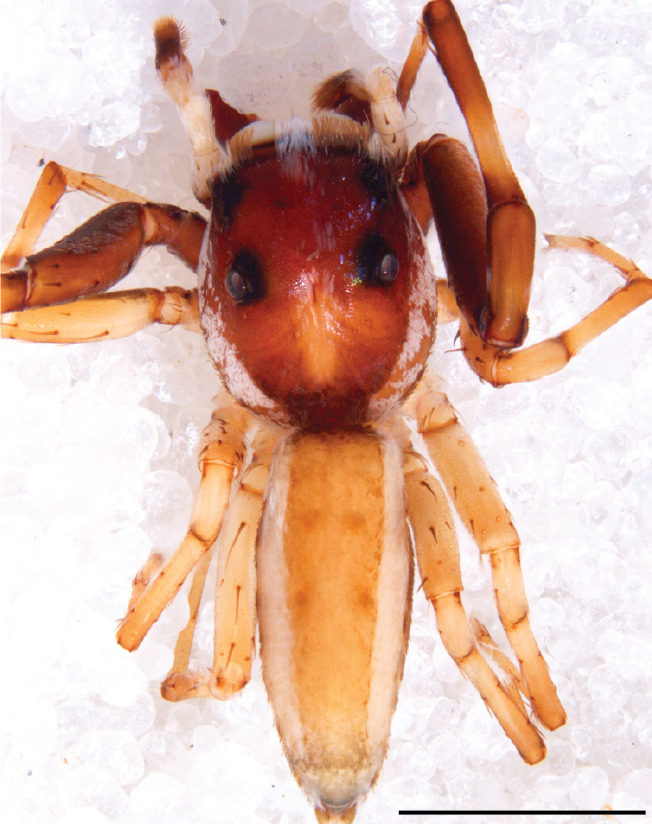
*Phintelloidesscandens* sp. nov., male habitus, dorsal view, RMNH.ARA.18255, scale bar 2 mm

**Figure 12b.**
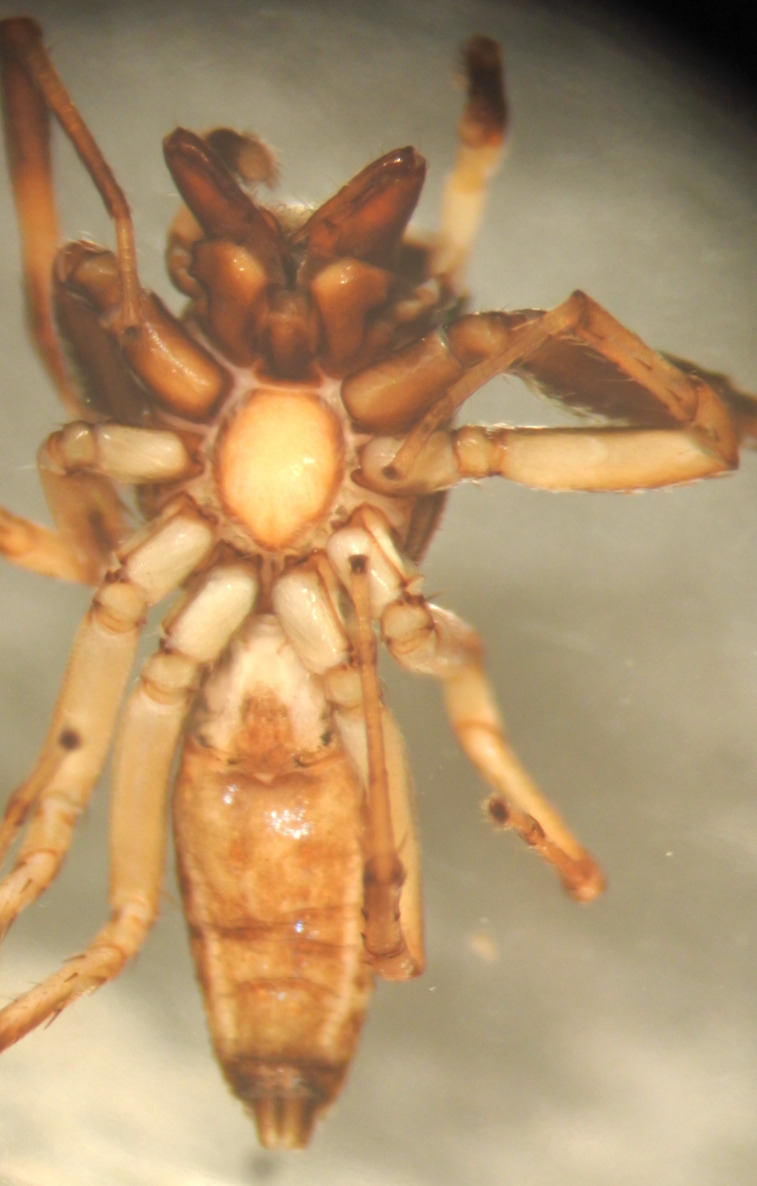
*Phintelloidesscandens* sp. nov., male habitus, ventral view, RMNH.ARA.18255

**Figure 12c.**
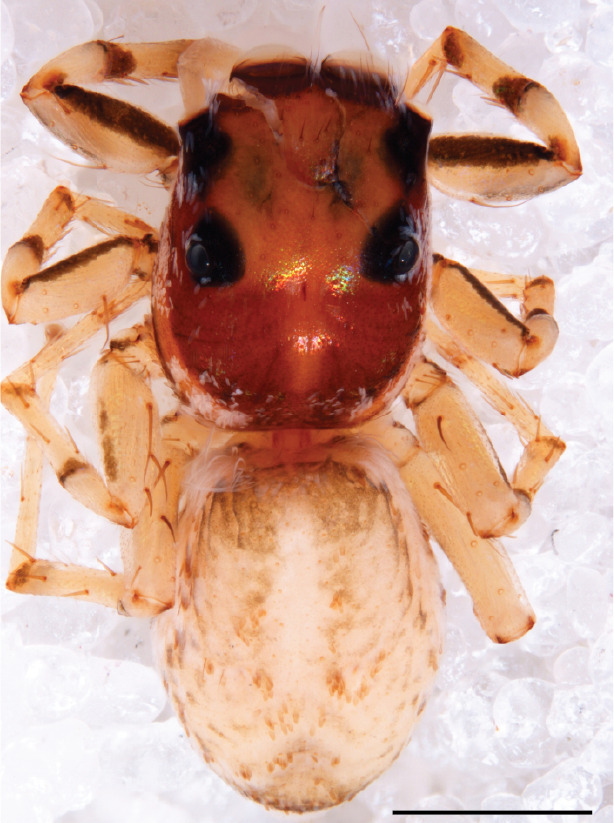
*Phintelloidesscandens* sp. nov., female habitus, dorsal view, RMNH.ARA.18257, scale bar 1 mm

**Figure 12d.**
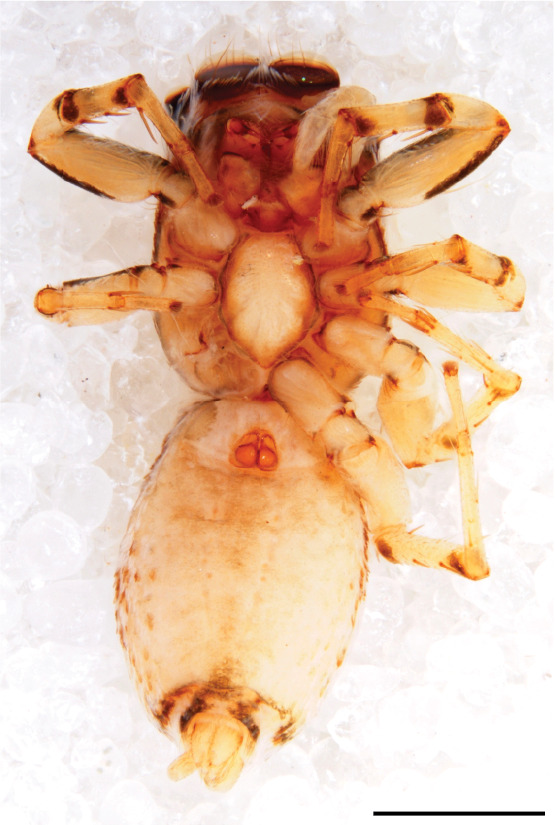
*Phintelloidesscandens* sp. nov., female habitus, ventral view, RMNH.ARA.18257, scale bar 1 mm

**Figure 13a.**
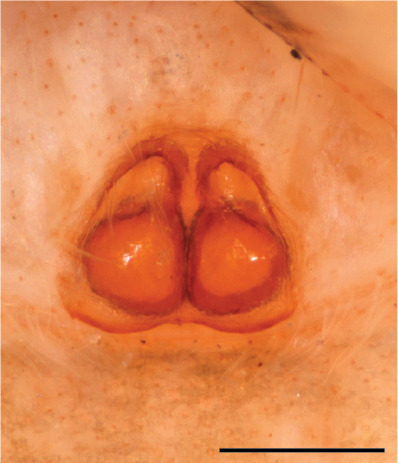
*Phintelloidesscandens* sp. nov., female epigynum, ventral view, RMNH.ARA.18257, scale bars 0.2 mm

**Figure 13b.**
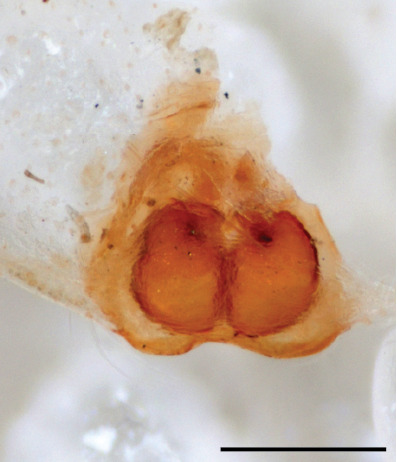
*Phintelloidesscandens* sp. nov., female vulva, dorsal view, RMNH.ARA.18257, scale bars 0.2 mm

**Figure 13c.**
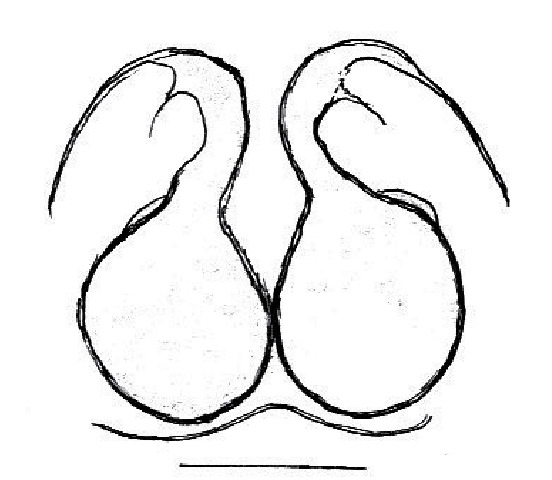
*Phintelloidesscandens* sp. nov., female epigynum, ventral view, illustration, scale bars 0.2 mm

**Figure 13d.**
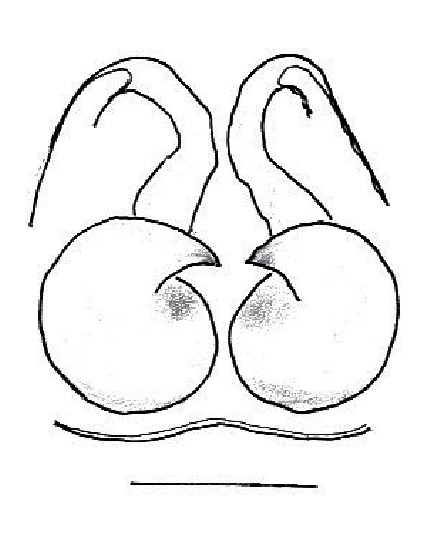
*Phintelloidesscandens* sp. nov., female vulva, dorsal view, illustration, scale bars 0.2 mm

**Figure 14a.**
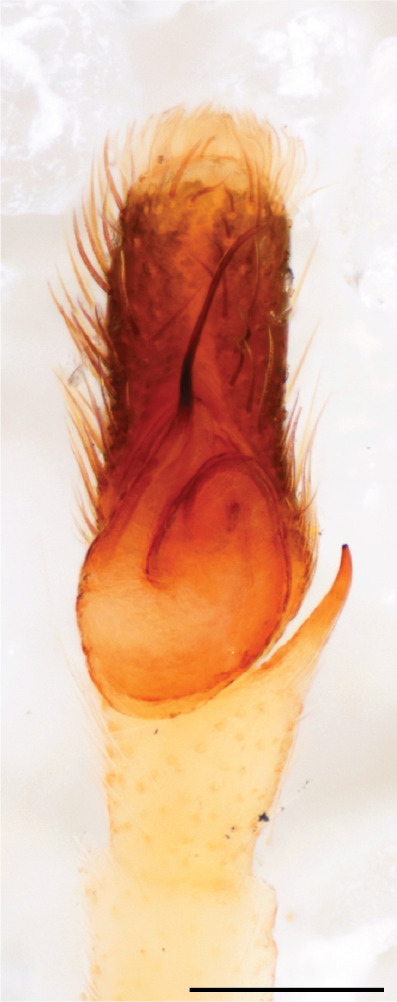
*Phintelloidesscandens* sp. nov., male holotype, pedipalp, ventral view, RMNH.ARA.18251, scale bar 0.2 mm

**Figure 14b.**
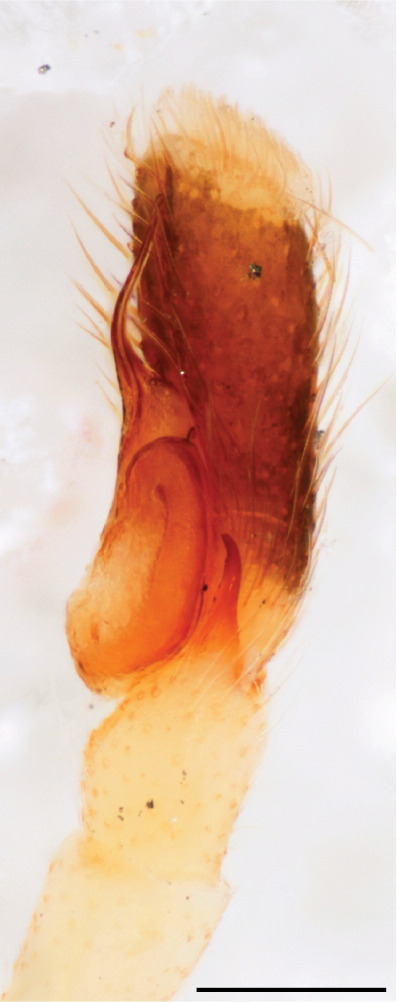
*Phintelloidesscandens* sp. nov., male holotype, pedipalp, retrolateral view, RMNH.ARA.18251, scale bar 0.2 mm

**Figure 14c.**
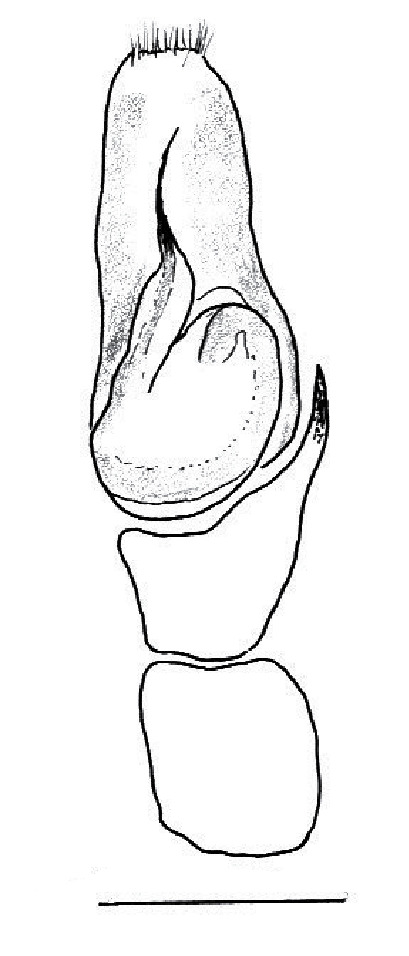
*Phintelloidesscandens* sp. nov., male pedipalp, ventral view, illustration, scale bar 0.2 mm

**Figure 14d.**
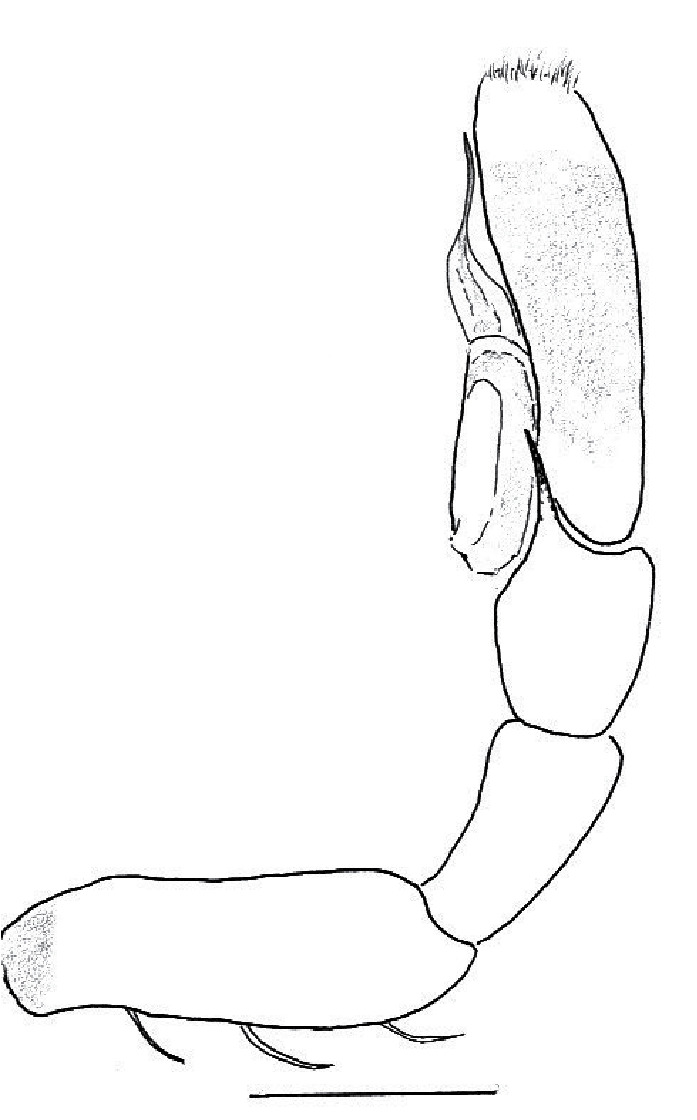
*Phintelloidesscandens* sp. nov., male pedipalp, retrolateral view, illustration, scale bar 0.2 mm

**Figure 14e.**
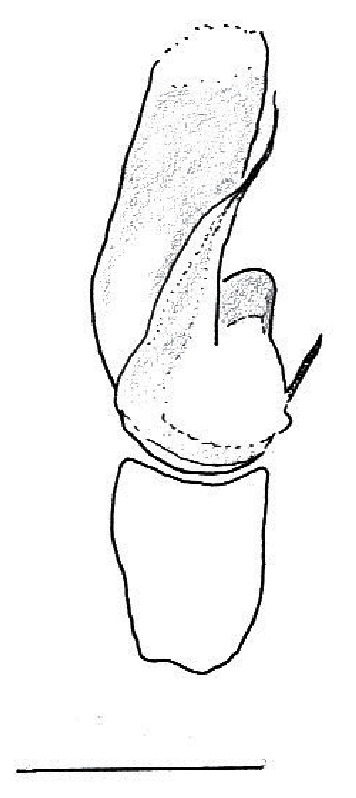
*Phintelloidesscandens* sp. nov., male pedipalp, prolateral view, illustration, scale bar 0.2 mm

**Figure 14f.**
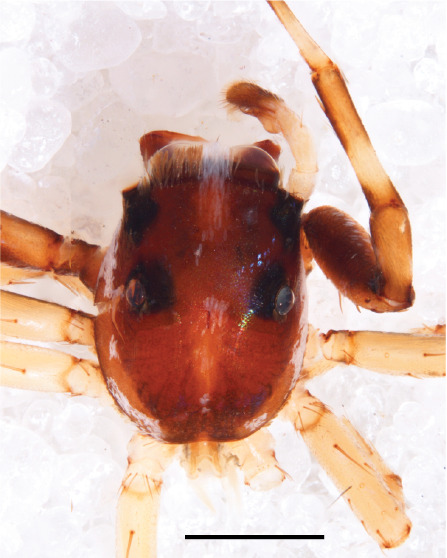
*Phintelloidesscandens* sp. nov., male holotype, prosoma, dorsal view, RMNH.ARA.18251, scale bar 1 mm

**Figure 15a.**
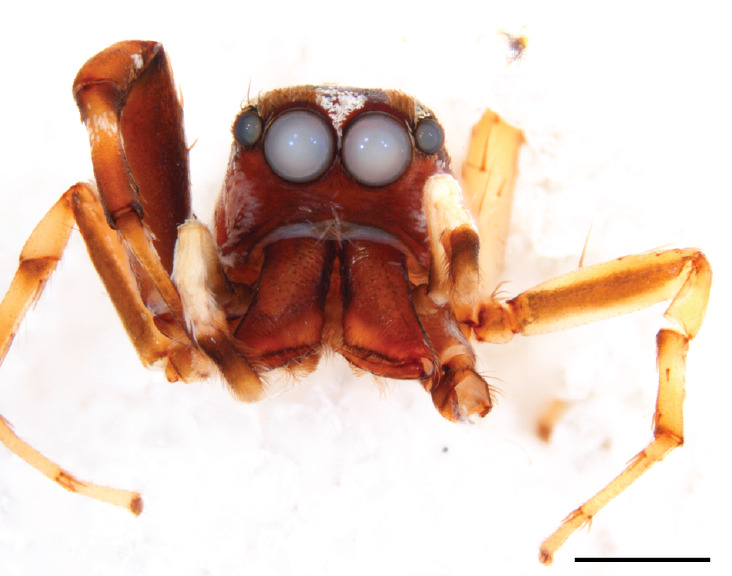
*Phintelloidesscandens* sp. nov., male prosoma, anterior view, RMNH.ARA.18255, scale bar 1 mm

**Figure 15b.**
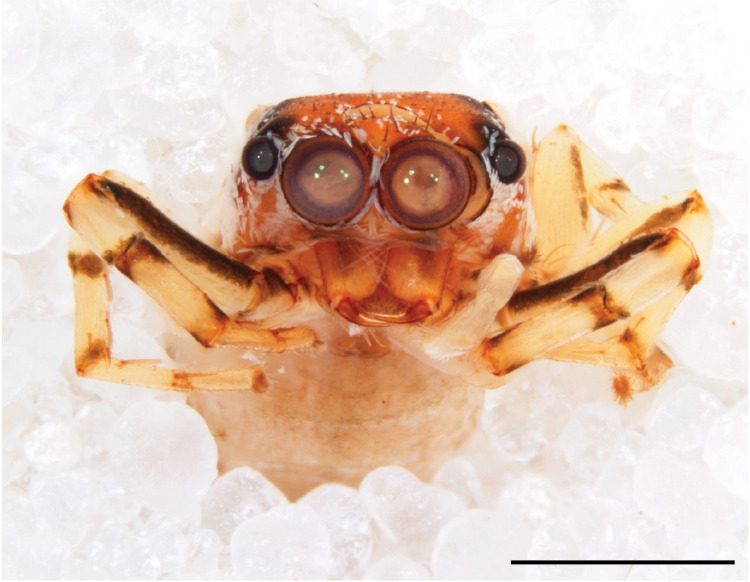
*Phintelloidesscandens* sp. nov., female prosoma, anterior view, RMNH.ARA.18257, scale bar 1 mm

**Figure 15c.**
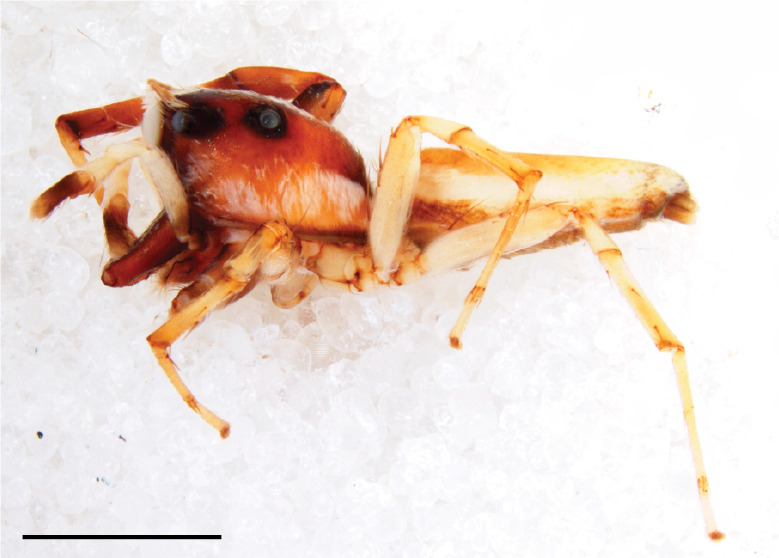
*Phintelloidesscandens* sp. nov., male habitus, lateral view, RMNH.ARA.18255, scale bar 2 mm

**Figure 15d.**
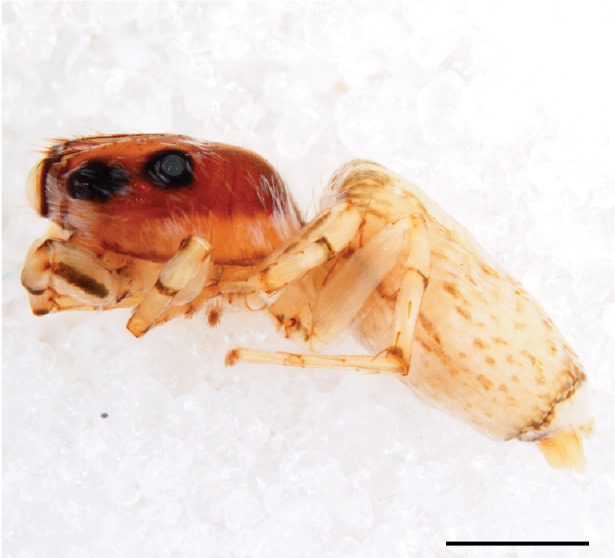
*Phintelloidesscandens* sp. nov., female habitus, lateral view, RMNH.ARA.18257, scale bar 1 mm

**Figure 17a.**
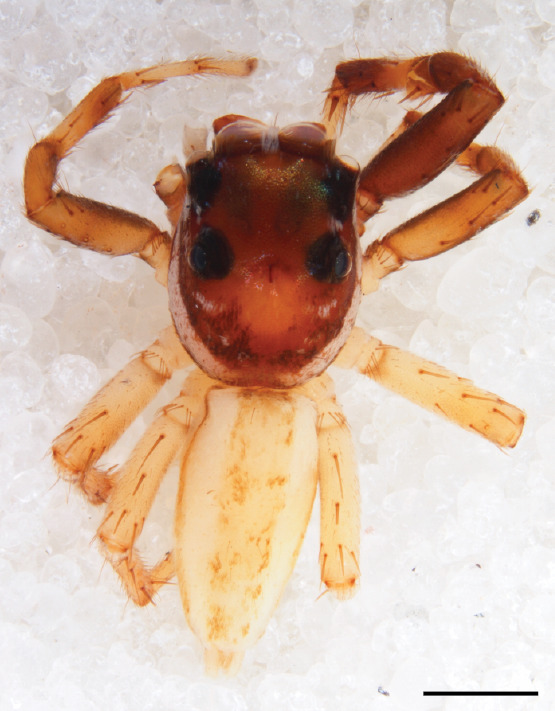
*Phintelloidesversicolor* (C. L. Koch, 1846), male habitus, dorsal view, RMNH.ARA.18261

**Figure 17b.**
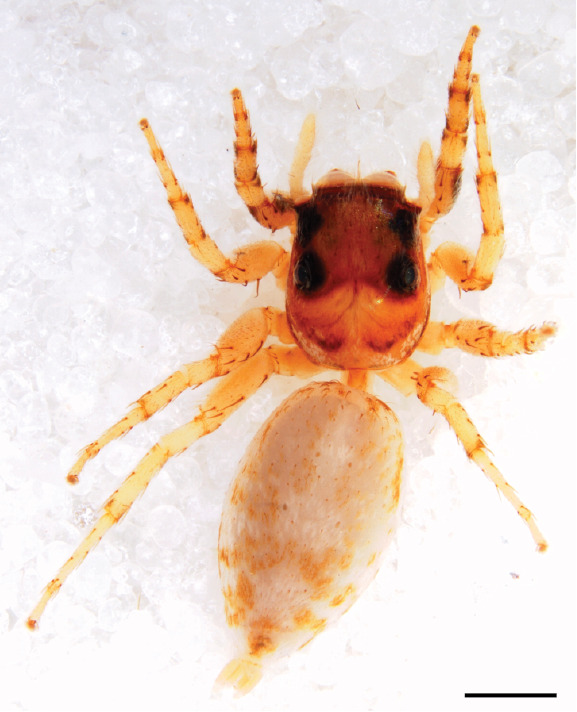
*Phintelloidesversicolor* (C. L. Koch, 1846), female habitus, dorsal view, CM 19264

**Figure 17c.**
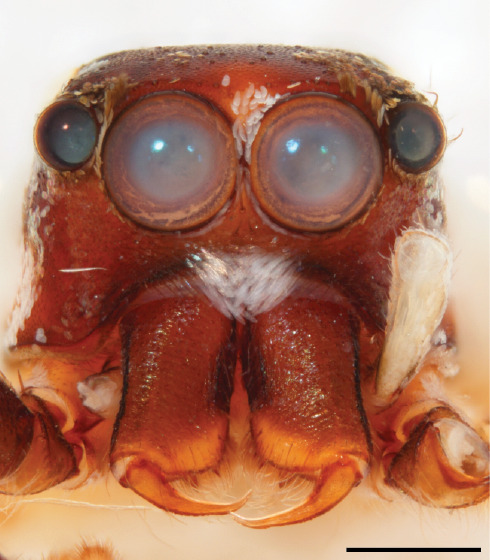
*Phintelloidesversicolor* (C. L. Koch, 1846), male face, RMNH.ARA.18261

**Figure 17d.**
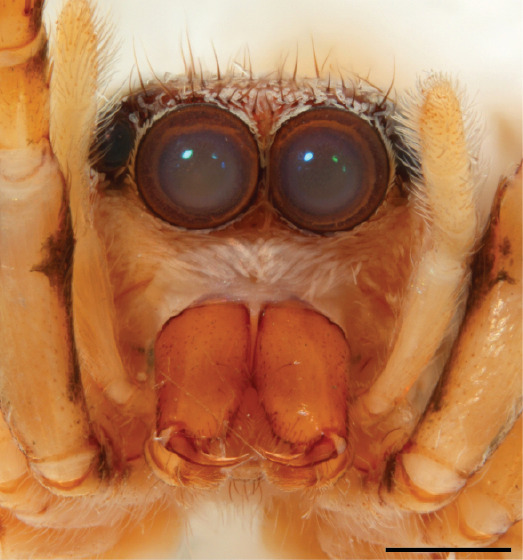
*Phintelloidesversicolor* (C. L. Koch, 1846), female face, CM 19264

**Figure 17e.**
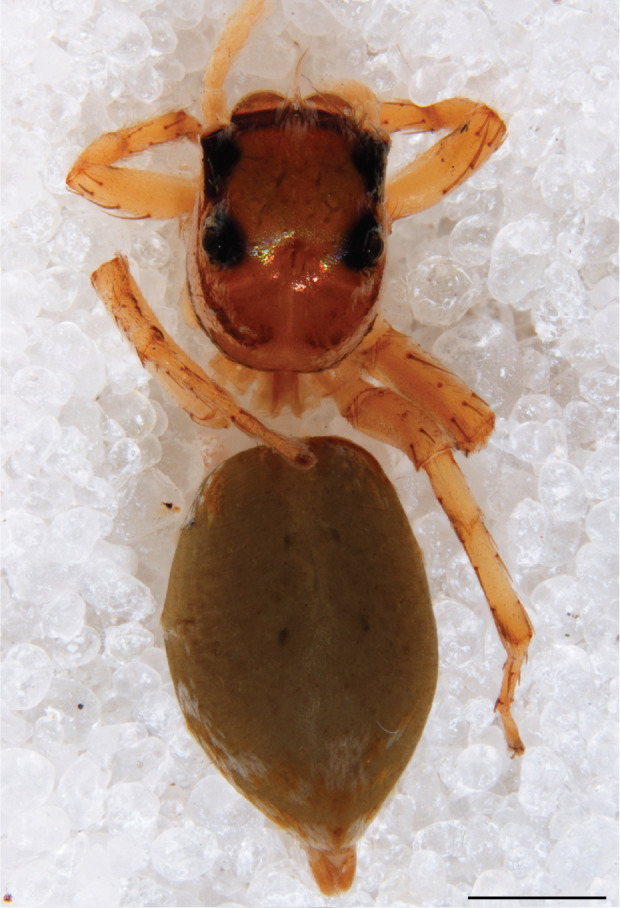
*Phintelloidesmunita* (Bösenberg & Strand, 1906), female habitus, dorsal view, CM 15605

**Figure 17f.**
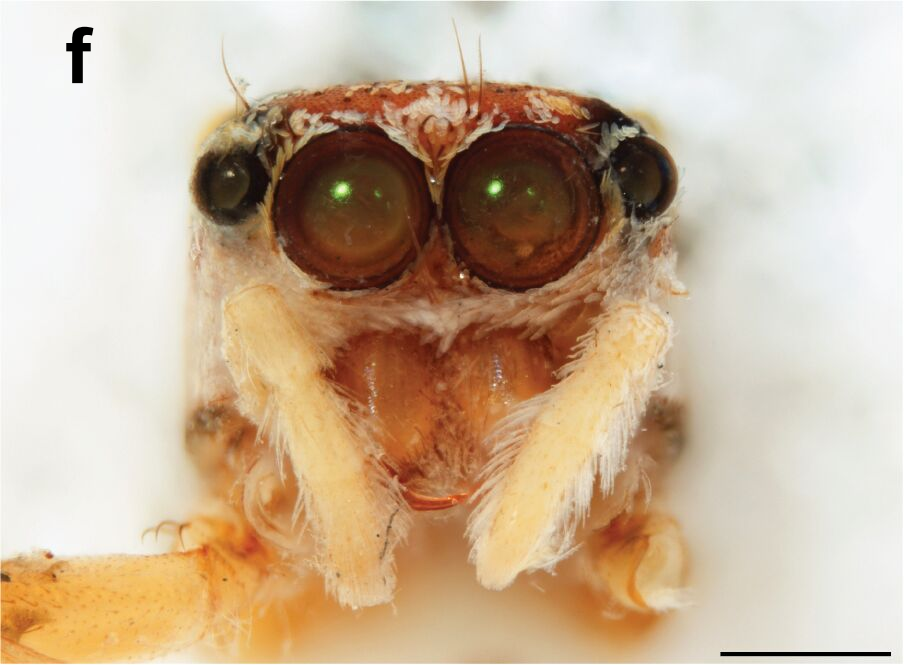
*Phintelloidesmunita* (Bösenberg & Strand, 1906), female face, CM 15605

**Figure 18a.**
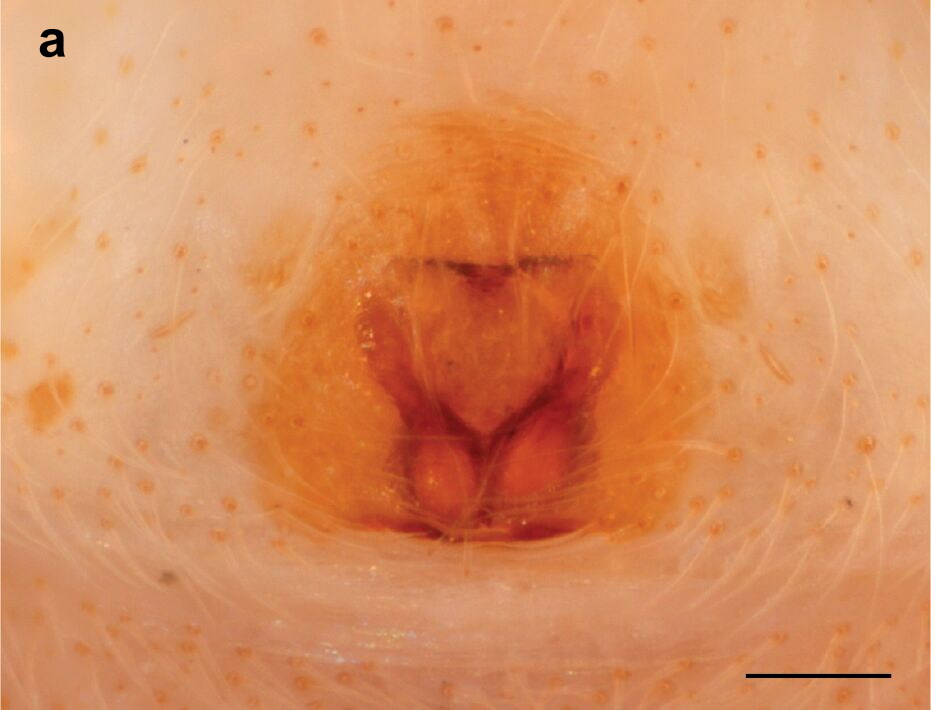
*Phintelloidesversicolor* (C. L. Koch, 1846), female epigynum, ventral view, CM 19264, scale bar 0.1 mm

**Figure 18b.**
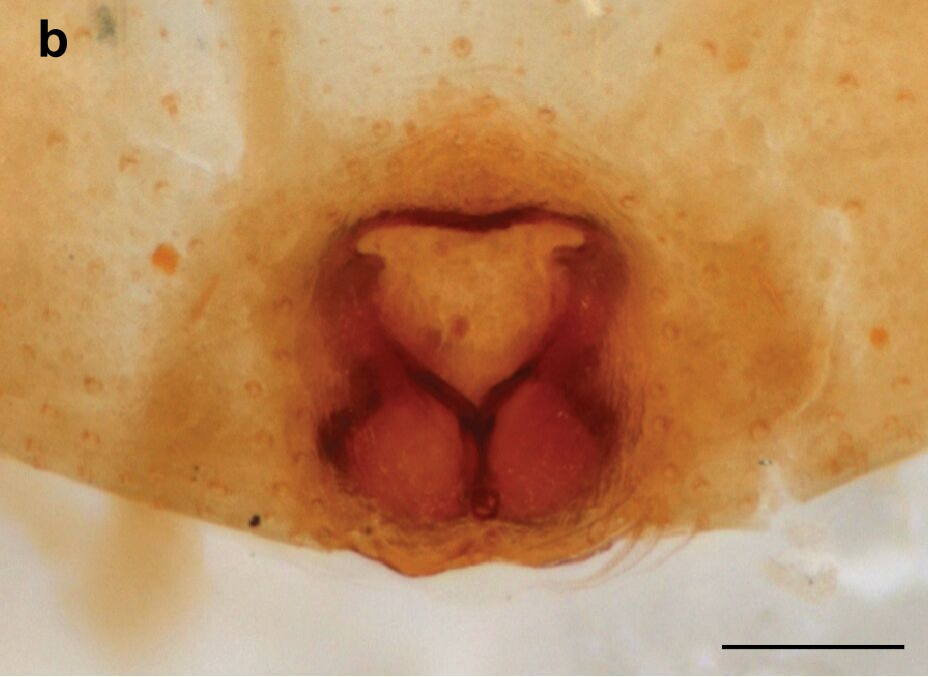
*Phintelloidesmunita* (Bösenberg & Strand, 1906), female epigynum, ventral view, CM 15605, scale bar 0.1 mm

**Figure 18c.**
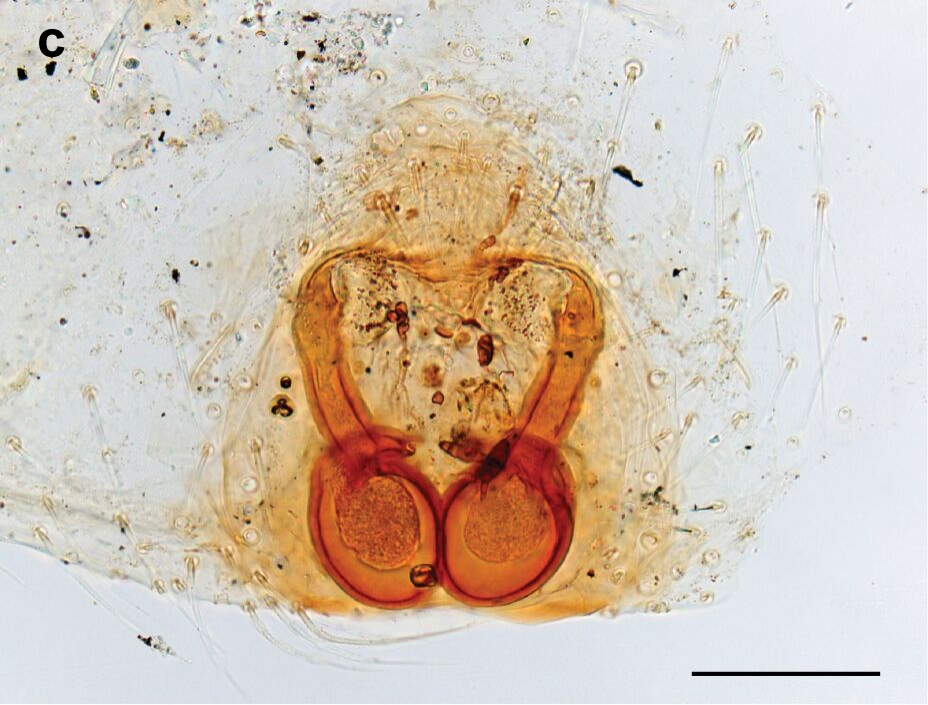
*Phintelloidesversicolor* (C. L. Koch, 1846), female vulva, dorsal view, RMNH.ARA.18260, scale bar 0.1 mm

**Figure 18d.**
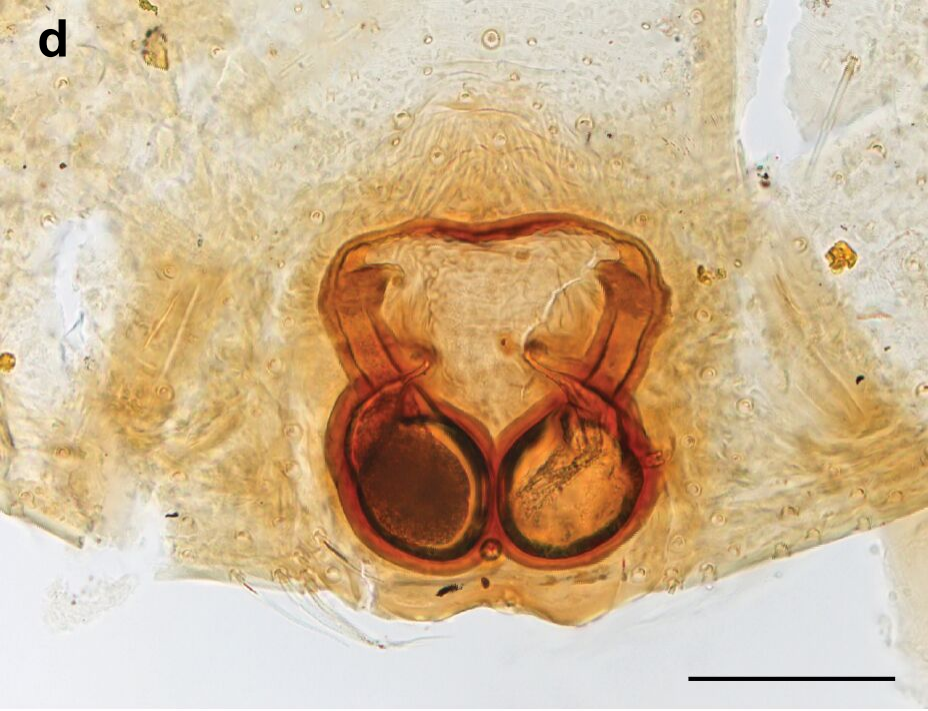
*Phintelloidesmunita* (Bösenberg & Strand, 1906), female vulva, dorsal view, CM 15605, scale bar 0.1 mm

**Figure 18e.**
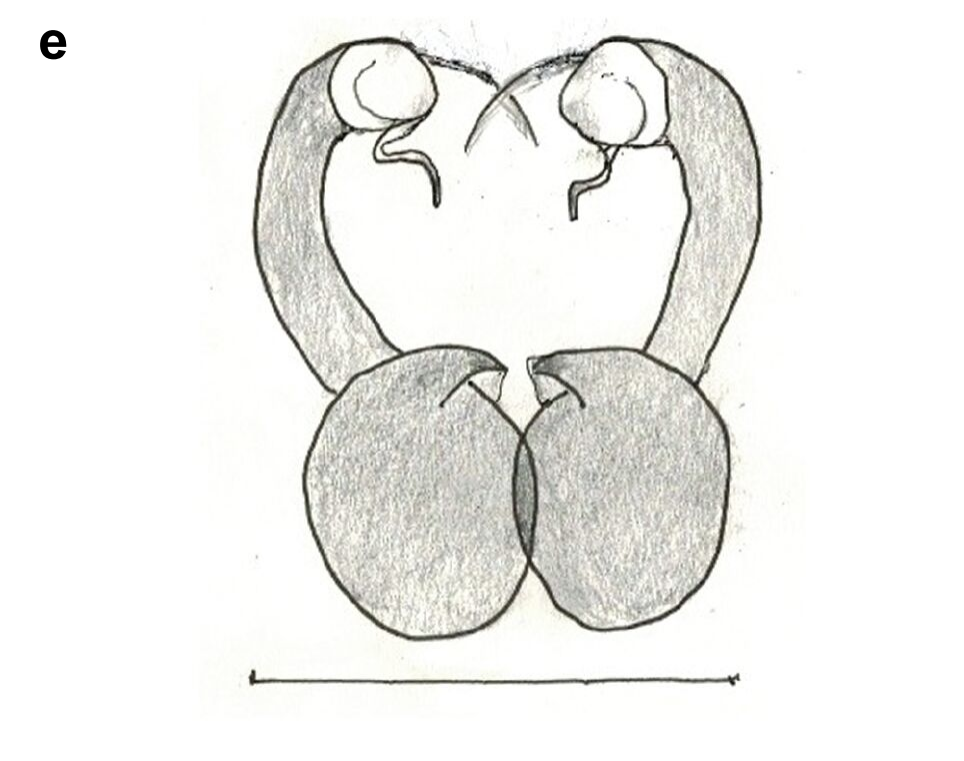
*Phintelloidesversicolor* (C. L. Koch, 1846), female epigynum, dorsal view, illustration, scale bar 0.2 mm

**Figure 18f.**
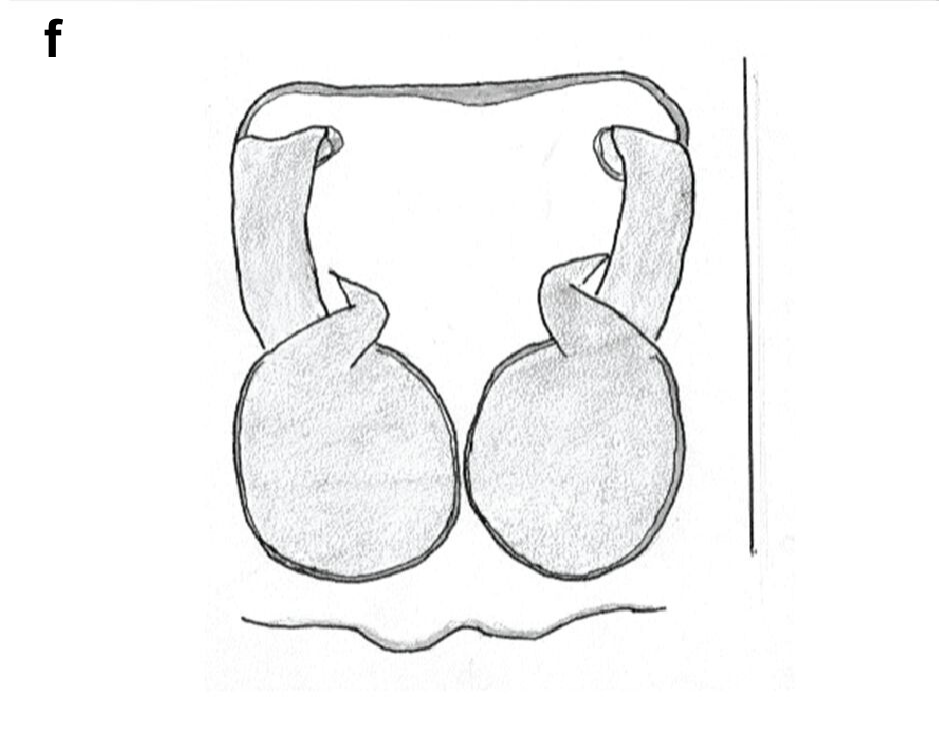
*Phintelloidesmunita* (Bösenberg & Strand, 1906), female epigynum, dorsal view, illustration, scale bar 0.2 mm

**Figure 19a.**
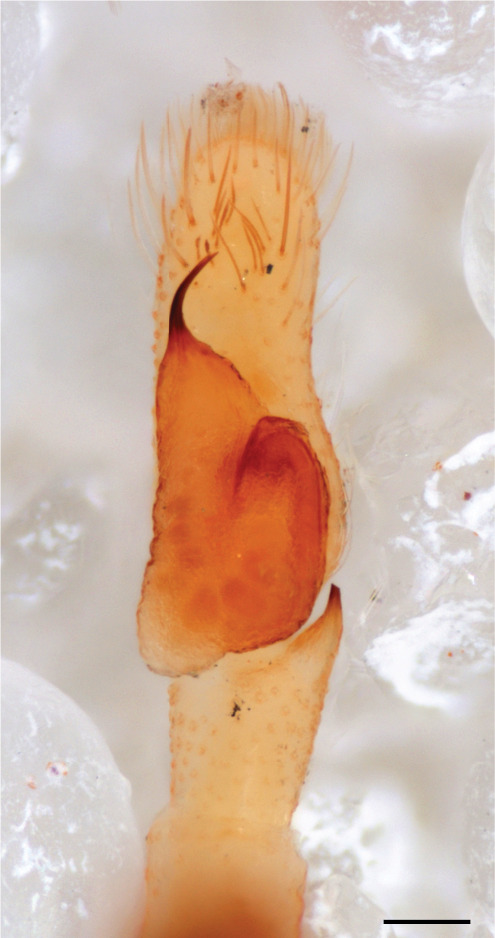
*Phintelloidesversicolor* (C. L. Koch, 1846), male pedipalp, ventral view, RMNH.ARA.18262

**Figure 19b.**
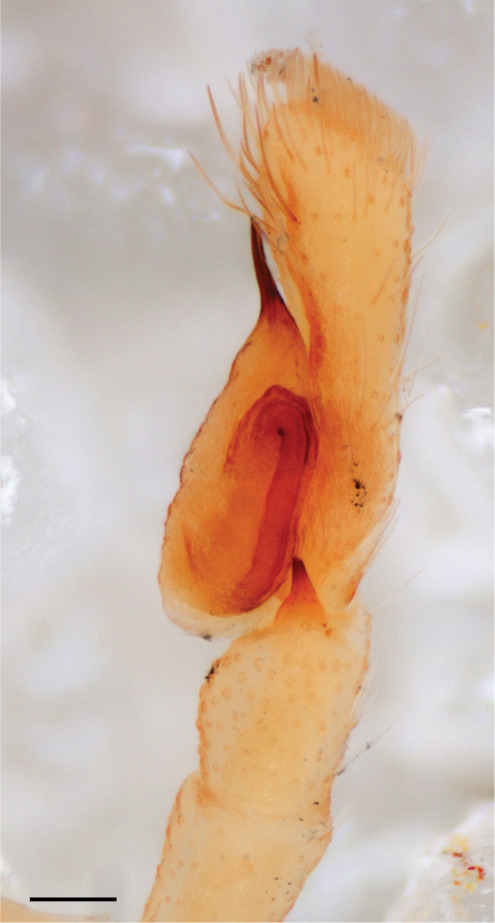
*Phintelloidesversicolor* (C. L. Koch, 1846), male pedipalp, retrolateral view, RMNH.ARA.18262

**Table 1.** Digital specimen DOIs and institutional identifiers for the specimens cited. Recognied species are hyperlinked to their Catalog of Life (https://www.catalogueoflife.org/) record; our new species and the revalidated species *P.minuta* are linked to Zoobank (https://zoobank.org/) records created for them. Institutional identifiers are catalog numbers in the case of the University of Manchester collection (MMUE), and machine readable persistent identifiers (PIDs) in the case of the Naturalis collection (RMNH).

**Species**	**Digital Specimen DOI**	**Institutional Identifier (Material Entity ID)**
*Chrysilla lauta*	https://doi.org/10.3535/G0G-G7D-N5J	MMUE G7572.5441
*Chrysilla lauta*	https://doi.org/10.3535/PER-LNE-HEW	MMUE G7572.6430
*Chrysilla lauta*	https://doi.org/10.3535/HS2-8W8-F23	MMUE G7572.6440
*Chrysilla lauta*	https://doi.org/10.3535/SGZ-EFZ-VRK	CM 19182
*Chrysilla volupe*	https://doi.org/10.3535/67X-9R9-YCM	MMUE 7572.6434
*Chrysilla volupe*	https://doi.org/10.3535/6H9-R1R-330	https://data.biodiversitydata.nl/naturalis/specimen/RMNH.ARA.18249
*Chrysilla volupe*	https://doi.org/10.3535/WL8-0R1-42B	https://data.biodiversitydata.nl/naturalis/specimen/RMNH.ARA.18259
*Chrysilla deelemani*	https://doi.org/10.3535/VYQ-YW1-AGE	https://data.biodiversitydata.nl/naturalis/specimen/RMNH.ARA.18264
*Chrysilla deelemani*	https://doi.org/10.3535/Z2J-WMP-FDH	https://data.biodiversitydata.nl/naturalis/specimen/RMNH.ARA.18265
*Phintelloides flavumi*	https://doi.org/10.3535/B59-03B-FWV	https://data.biodiversitydata.nl/naturalis/specimen/RMNH.ARA.18250
*Phintelloides jesudasi*	https://doi.org/10.3535/SVV-BR5-KGE	https://data.biodiversitydata.nl/naturalis/specimen/RMNH.ARA.18258
*Phintelloides scandens*, sp. nov.	https://doi.org/10.3535/5SG-PLB-MHT	https://data.biodiversitydata.nl/naturalis/specimen/RMNH.ARA.18251
*Phintelloides scandens*, sp. nov.	https://doi.org/10.3535/85R-G3E-4M0	https://data.biodiversitydata.nl/naturalis/specimen/RMNH.ARA.18252
*Phintelloides scandens*, sp. nov.		https://data.biodiversitydata.nl/naturalis/specimen/RMNH.ARA.18253
*Phintelloides scandens*, sp. nov.		https://data.biodiversitydata.nl/naturalis/specimen/RMNH.ARA.18254
*Phintelloides scandens*, sp. nov.		https://data.biodiversitydata.nl/naturalis/specimen/RMNH.ARA.18255
*Phintelloides scandens*, sp. nov.		https://data.biodiversitydata.nl/naturalis/specimen/RMNH.ARA.18256
*Phintelloides scandens*, sp. nov.		https://data.biodiversitydata.nl/naturalis/specimen/RMNH.ARA.18257
*Phintelloides versicolor*	https://doi.org/10.3535/C69-M7K-VWC	CM 21848
*Phintelloides versicolor*	https://doi.org/10.3535/3NW-1BX-8BK	MMUE G7572.6413
*Phintelloides versicolor*	https://doi.org/10.3535/M42-Z4P-DRD	https://data.biodiversitydata.nl/naturalis/specimen/RMNH.ARA.18260
*Phintelloides versicolor*	https://doi.org/10.3535/5MR-J6N-26M	https://data.biodiversitydata.nl/naturalis/specimen/RMNH.ARA.18261
*Phintelloides versicolor*	https://doi.org/10.3535/Q6C-91C-BS5	https://data.biodiversitydata.nl/naturalis/specimen/RMNH.ARA.18262
*Phintelloides munita*, revalidated	https://doi.org/10.3535/MDR-6FG-49E	MMUE G7572.6412

**Table 2.** Wikidata identifiers for collectors and other human agents cited in the specimen data.

**Name string**	**Full Name**	**Wikidata QID**
F. Murphy	Frances Murphy	Q22111840
J. A. Murphy	John A. Murphy	Q22113060
P. R. Deeleman	Paul Robert Deeleman	Q60057036
C. L. Deeleman	Christa Deeleman-Reinhold	Q2964921
A. Floren	Andreas Floren	Q23068668
P. Schwendinger	Peter Schwendinger	Q7174897
W. Corley	Wendy Corley	Q125189589
S. Djojosudharmo	Suharto Djojosudharmo	Q125189757

## References

[B11301734] Addink W., Theocharides S., Islam S. (2023). A novel part in the Swiss Army Knife for linking biodiversity data: The digital specimen identifier service. Biodiversity Information Science and Standards.

[B11471977] Agosti D., Egloff W. (2009). Taxonomic information exchange and copyright: the Plazi approach. BMC Research Notes.

[B11566214] Arzuza Buelvas D. (2018). The Murphy spider collection at the Manchester Museum: a valuable research resource for arachnologists. Journal of Natural Science Collections.

[B11297518] Barrion A. T., Barrion-Dupo A. L. A., Catindig J. L. A., Villareal M. O., Cai D., Yuan Q. H., Heong K. L. (2013). New species of spiders (Araneae) from Hainan Island, China. UPLB Museum Publications in Natural History.

[B11244130] Bohdanowicz A., Prószyński J. (1987). Systematic studies on East Palaearctic Salticidae (Araneae), IV. Salticidae of Japan. Annales Zoologici, Warszawa.

[B11246511] Bösenberg W., Strand E. (1906). Japanische Spinnen. Abhandlungen der Senckenbergischen Naturforschenden Gesellschaft.

[B11243974] Brignoli P. M. (1985). On the correct dates of publication of the arachnid taxa described in some works by C. W. Hahn and C. L. Koch (Arachnida). Bulletin of the British Arachnological Society.

[B11211309] Caleb J. T.D., Sanap R. V., Patel K. G., Sudhin P. P., Nafin K. S., Sudhikumar A. V. (2018). First description of the female of *Chrysillavolupe* (Karsch, 1879) (Araneae: Salticidae: Chrysillini) from India, with notes on the species’ distribution and life history. Arthropoda Selecta.

[B11243851] Caleb J. T.D. (2020). Spider (Arachnida: Araneae) fauna of the scrub jungle in the Madras Christian College campus, Chennai, India. Journal of Threatened Taxa.

[B11236770] Caleb J. T. D., Mathai M. T. (2014). Description of some interesting jumping spiders (Araneae: Salticidae) from South India. Journal of Entomology and Zoology Studies.

[B11236779] Caleb J. T. D. (2016). New data on the jumping spiders (Araneae: Salticidae) from India. Arthropoda Selecta.

[B11297538] Chen K. - M., Lin T. - Y., Ueng Y. - T. (2021). Three new species and six newly recorded species of jumping spiders (Araneae: Salticidae) in Taiwan. Natural Resources.

[B11294339] Chen X. E, Gao J. C. (1990). The Sichuan farmland spiders in China.

[B11294373] Chen Z. F., Zhang Z. H. (1991). Fauna of Zhejiang: Araneida.

[B11294294] Chikuni Y. (1989). Pictorial encyclopedia of spiders in Japan.

[B11294516] Cho J. H., Kim J. P. (2002). A revisional study of family Salticidae Blackwall, 1841 (Arachnida, Araneae) from Korea. Korean Arachnology.

[B11211204] Deeleman-Reinhold C. L., Miller J. A., Floren A. (2016). *Depreissiadecipiens*, an enigmatic canopy spider from Borneo revisited (Araneae, Salticidae), with remarks on the distribution and diversity of canopy spiders in Sabah, Borneo. ZooKeys.

[B11301672] Fawcett Susan, Agosti Donat, Cole Selina R., Wright David F. (2022). Digital accessible knowledge: Mobilizing legacy data and the future of taxonomic publishing. Bulletin of the Society of Systematic Biologists.

[B11297497] Feng Z. Q. (1990). Spiders of China in colour.

[B11211179] Floren A., Deeleman-Reinhold C. L. (2005). Diversity of arboreal spiders in primary and disturbed tropical forests. Journal of Arachnology.

[B11395737] Hardisty Alex R., Ellwood Elizabeth R., Nelson Gil, Zimkus Breda, Buschbom Jutta, Addink Wouter, Rabeler Richard K., Bates John, Bentley Andrew, Jos&eacute A. B.Fortes, Hansen Sara, Macklin James A., Mast Austin R., Miller Joseph T., Monfils Anna K., Paul Deborah L., Wallis Elycia, Webster Michael (2022). Digital Extended Specimens: Enabling an Extensible Network of Biodiversity Data Records as Integrated Digital Objects on the Internet. BioScience.

[B11296646] Hu J. L. (1984). The Chinese spiders collected from the fields and the forests.

[B11294474] Hu J. L. (2001). Spiders in Qinghai-Tibet Plateau of China.

[B11211291] Kanesharatnam N., Benjamin S. P. (2019). Multilocus genetic and morphological phylogenetic analysis reveals a radiation of shiny South Asian jumping spiders (Araneae, Salticidae). ZooKeys.

[B11236739] Karsch F. (1879). Arachnologische Beiträge. Zeitschrift für die Gesammten Naturwissenschaften.

[B11294673] Kim S. T, Lee S. Y. (2014). Arthropoda: Arachnida: Araneae: Clubionidae, Corinnidae, Salticidae, Segestriidae. Spiders.. Invertebrate Fauna of Korea.

[B11243944] Koch C. L. (1846). Die Arachniden.

[B11243965] Koch C. L. (1848). Die Arachniden.

[B11297922] Koh J. K.H. (1989). A Guide to Common Singapore Spiders.

[B11211230] Koh J. K.H., Ming L. T. (2013). Spiders of Brunei Darussalam. Biodiversity in the heart of Borneo.

[B11211188] Koh J. K.H., Bay N. (2019). Borneo Spiders, A Photographic Field Guide.

[B11211265] Koh J. K.H., Court D. J., Ang C. S.P., Ng P. Y.C. (2022). A Photographic Guide to Singapore Spiders.

[B11405441] Kozub D., Shapoval J., Yatsenko S., Starikh V., Dobarskyi A. (2000). Helicon Focus.

[B11296576] Lee C. L. (1966). Spiders of Formosa.

[B11297547] Lin Y. J., Wu L. B., Cai D. C., Li S. Q., Barrion A. T., Heong K. L. (2023). Review of 43 spider species from Hainan Island, China (Arachnida, Araneae). *Zootaxa*.

[B11297488] Maddison W. (1987). *Marchena* and other jumping spiders with an apparent leg-carapace stridulatory mechanism (Araneae: Salticidae: Heliophaninae and Thiodinae). Bulletin of the British Arachnological Society.

[B11294411] Maddison W. P. (1996). *Pelegrina* Franganillo and other jumping spiders formerly placed in the genus *Metaphidippus* (Araneae: Salticidae). Bulletin of the Museum of Comparative Zoology.

[B11211221] Maddison W. P. (2015). A phylogenetic classification of jumping spiders (Araneae: Salticidae). Journal of Arachnology.

[B11236797] Magar K. T., Shrestha B. R., Gurung T. B., Bahadur R., Lamichhane B. R., Hill D. E., Thapa A. (2020). New records of jumping spiders (Araneae: Salticidae) from Nepal. Peckhamia.

[B11294279] Matsumoto S., Nishikawa Y., Ono H. (1989). Arachnological Papers Presented to Takeo Yaginuma on the Occasion of his Retirement.

[B11211196] Metzner H. (1996-2020). Jumping spiders of the world (Arachnida, Araneae, Salticidae).

[B11301681] Miller Jeremy, Agosti Donat, Penev Lyubomir, Sautter Guido, Georgiev Teodor, Catapano Terry, Patterson David, King David, Pereira Serrano, Vos Rutger, Sierra Soraya (2015). Integrating and visualizing primary data from prospective and legacy taxonomic literature. Biodiversity Data Journal.

[B11294490] Namkung J. (2002). The spiders of Korea.

[B11294596] Namkung J. (2003). The Spiders of Korea.

[B11294612] Ono H., Ikeda H., Kono R., Ono H. (2009). The spiders of Japan with keys to the families and genera and illustrations of the species.

[B11301661] Penev L., Erwin T., Miller J., Chavan V., Moritz T., Griswold C. (2009). Publication and dissemination of datasets in taxonomy: ZooKeys working example. ZooKeys.

[B11301706] Penev Lyubomir, Koureas Dimitrios, Groom Quentin, Lanfear Jerry, Agosti Donat, Casino Ana, Miller Joe, Arvanitidis Christos, Cochrane Guy, Hobern Donald, Banki Olaf, Addink Wouter, Kõljalg Urmas, Copas Kyle, Mergen Patricia, Güntsch Anton, Benichou Laurence, Lopez Jose Benito Gonzalez, Ruch Patrick, Martin Corinne S., Barov Boris, Demirova Iliyana, Hristova Kristina (2022). Biodiversity Community Integrated Knowledge Library (BiCIKL). Research Ideas and Outcomes.

[B11294381] Peng X. J., Xie L. P., Xiao X. Q., Yin C. M. (1993). Salticids in China (Arachnida: Araneae).

[B11231575] Peng X. J. (2020). Fauna Sinica, Invertebrata 53, Arachnida: Araneae: Salticidae.

[B11244053] Prószyński J. (1973). Systematic studies on east Palaearctic Salticidae, II. Redescriptions of Japanese Salticidae of the Zoological Museum in Berlin. Annales Zoologici, Warszawa.

[B11297913] Prószyński J. (1976). Studium systematyczno-zoogeograflczne nad rodziną Salticidae (Aranei) Regionów Palearktycznego i Nearktycznego. Wyższa Szkola Pedagogiczna Siedlcach.

[B11244121] Prószyński J. (1978). Distributional patterns of the Palaearctic Salticidae (Araneae). Symposia of the Zoological Society of London.

[B11231500] Prószyński J. (1983). Position of genus *Phintella* (Araneae: Salticidae). Acta Arachnologica.

[B11294192] Prószyński J. (1983). Redescriptions of *Phintellatypica* and *Telamoniabifurcilinea* (Araneae: Salticidae). Acta Arachnologica.

[B11211273] Prószyński J. (1984). Atlas rysunków diagnostycznych mniej znanych Salticidae (Araneae). Zeszyty Naukowe Wyższej Szkoły Rolniczo-Pedagogicznej w Siedlcach.

[B11236761] Prószyński J. (1985). On *Siler*, *Silerella*, *Cyllobelus* and *Natta* (Araneae, Salticidae). *Annales Zoologici, Warszawa*.

[B11244153] Prószyński J. (1987). Atlas rysunków diagnostycznych mniej znanych Salticidae 2.

[B11231548] Prószyński J., Deeleman-Reinhold C. L. (2010). Description of some Salticidae (Araneae) from the Malay Archipelago. I. Salticidae of the Lesser Sunda Islands, with comments on related species. *Arthropoda Selecta*.

[B11211282] Prószyński J., Deeleman-Reinhold C. L. (2012). Description of some Salticidae (Aranei) from the Malay archipelago. II. Salticidae of Java and Sumatra, with comments on related species. Arthropoda Selecta.

[B11211248] Prószyński J. (2016). Monograph of Salticidae (Araneae) of the World 1995-2015. Part II. Global Species Database of Salticidae (Araneae). Version October 30th, 2016.

[B11294682] Prószyński J. (2017). Pragmatic classification of the world's Salticidae (Araneae). Ecologica Montenegrina.

[B11231557] Prószyński J. (2018). Review of the genus *Hasarius* (Araneae: Salticidae) - a taxonomic fiasco. Ecologica Montenegrina.

[B11231482] Roewer C. F. (1955). Katalog der Araneae von 1758 bis 1940, bzw. 1954. 2. Band, Abt. a (Lycosaeformia, Dionycha [excl. Salticiformia]). 2. Band, Abt. b (Salticiformia, Cribellata) (Synonyma-Verzeichnis, Gesamtindex).

[B11296530] Saitō S. (1959). The Spider Book Illustrated in Colours.

[B11246747] Schenkel E. (1963). Ostasiatische Spinnen aus dem Muséum d'Histoire naturelle de Paris. Mémoires du Muséum National d'Histoire Naturelle de Paris (A, Zool.).

[B11231188] Simon E. (1901). Histoire naturelle des araignées.

[B11231509] Simon E. (1903). Etudes arachnologiques. 33e Mémoire. LIII. Arachnides recueillis à Phuc-Son (Annam) par M. H. Fruhstorfer (nov-dec. 1899). Annales de la Société Entomologique de France.

[B11244035] Simon E. (1903). Etudes arachnologiques. 34e Mémoire. LIV. Arachnides recueillis à Sumatra par M. J. Bouchard. Annales de la Société Entomologique de France.

[B11246775] Song D. X. (1982). Some new records and synonyms of Chinese spiders. Zoological Research.

[B11297462] Song D. X. (1987). Spiders from agricultural regions of China (Arachnida: Araneae).

[B11231527] Song D. X., Chai J. Y., Qian Y. W. (1991). Animal Science Research.

[B11294420] Song D. X., Chen J., Zhu M. S., Yang X. K. (1997). Insects of the Three Gorge Reservoir area of Yangtze River.

[B11231540] Song D. X., Zhu M. S., Chen J. (1999). The spiders of China.

[B11246571] Strand E. (1907). Vorläufige Diagnosen süd- und ostasiatischer Clubioniden, Ageleniden, Pisauriden, Lycosiden, Oxyopiden und Salticiden. Zoologischer Anzeiger.

[B11211256] Szüts T. (2004). A revision of the genus *Bristowia*(Araneae: Salticidae). Folia Entomologica Hungarica.

[B11228809] Thorell T. (1887). Viaggio di L. Fea in Birmania e regioni vicine. II. Primo saggio sui ragni birmani. Annali del Museo Civico di Storia Naturale di Genova.

[B11243983] Thorell T. (1891). Spindlar från Nikobarerna och andra delar af södra Asien. Kongliga Svenska Vetenskaps-Akademiens Handlingar.

[B11236788] Thumar R. H., Dholakia A. H. (2018). First record of *Chrysillavolupe* Karsch, 1879 (Araneae: Salticidae) in agroecosystem of Navsari at Gujarat, India. Research Hub – International Multidisciplinary Research Journal.

[B11566177] van Dorp K. (2020). A life of spiders: Christa Deeleman and her collection. Nieuwsbrief Spined.

[B11243952] Walckenaer C. A., Walckenaer C. A., Gervais P. (1847). Histoire naturelles des Insects. Aptères.

[B11230938] Wang Lu-Yu, Zhang Zhi-Sheng (2012). A new species of *Chrysilla*Thorell, 1887 from China (Araneae: Salticidae). Zootaxa.

[B11294039] Wesołowska W. (1981). Salticidae (Aranei) from North Korea, China and Mongolia. Annales Zoologici, Warszawa.

[B11294174] Wesołowska W. (1981). Redescriptions of the E. Schenkel's East Asiatic Salticidae (Aranei). Annales Zoologici, Warszawa.

[B11243992] Workman T., Workman M. E. (1894). *Malaysian spider*.

[B11211213] Catalog World Spider (2024). World Spider Catalog. Version 25.0.

[B11297084] Yaginuma T. (1955). Revision of scientific names of Japanese spiders. Atypus.

[B11246629] Yaginuma T. (1960). Spiders of Japan in colour.

[B11246756] Yaginuma T. (1971). Spiders of Japan in colour.

[B11246765] Yaginuma T. (1977). A list of Japanese spiders (revised in 1977). Acta Arachnologica.

[B11294201] Yaginuma T. (1986). Spiders of Japan in color.

[B11211238] Yamasaki T., Yamaguchi M., Phung L. T. H., Huang P. S., Tso I. M. (2018). Redescription of *Chrysillalauta* Thorell 1887 (Araneae: Salticidae) based on the comparison with the holotype, and DNA barcoding. Acta Arachnologica.

[B11296584] Yin C. M., Wang J. F. (1979). A classification of the jumping spiders (Araneae, Salticidae) collected from the agricultural fields and other habitats. Journal of Hunan Teachers College (nat. Sci. Ed.).

[B11294653] Yin C. M., Peng X. J., Yan H. M., Bao Y. H., Xu X., Tang G., Zhou Q. S., Liu P. (2012). Fauna Hunan: Araneae in Hunan, China.

[B11211300] Żabka M. (1985). Systematic and zoogeographic study on the family Salticidae (Araneae) from Viet-Nam. Annales Zoologici, Warszawa.

[B11231623] Żabka M. (1988). Salticidae (Araneae) of Oriental, Australian and Pacific regions, III. Annales Zoologici, Warszawa.

[B11294389] Zhao J. Z. (1993). Spiders in the cotton fields in China.

[B11499330] Zhao J. Z. (1995). Natural enemies of cotton pests in China.

[B11294636] Zhu M. S., Zhang B. S. (2011). Spider fauna of Henan: Arachnida: Araneae.

